# Myeloarchitectonic maps of the human cerebral cortex registered to surface and sections of a standard atlas brain

**DOI:** 10.1515/tnsci-2022-0325

**Published:** 2023-12-26

**Authors:** Juergen K. Mai, Milan Majtanik

**Affiliations:** Department of Neuroanatomy, Heinrich Heine University Duesseldorf, Duesseldorf D-40225, Germany; Department of Informatics, Heinrich Heine University Duesseldorf, Duesseldorf D-40225, Germany; MRX-Brain GmbH Duesseldorf, Duesseldorf D-40225, Germany

**Keywords:** Vogt, myelin, microscopic sections, multi-level-stripe technology, cortical stripe representation

## Abstract

C. and O. Vogt had set up a research program with the aim of establishing a detailed cartography of the medullary fiber distribution of the human brain. As part of this program, around 200 cortical fields were differentiated based on their myeloarchitectural characteristics and mapped with regard to their exact location in the isocortex. The typical features were graphically documented and classified by a sophisticated linguistic coding. Their results have only recently received adequate attention and applications. The reasons for the revival of this spectrum of their research include interest in the myeloarchitecture of the cortex as a differentiating feature of the cortex architecture and function, as well as the importance for advanced imaging methodologies, particularly tractography and molecular imaging. Here, we describe our approach to exploit the original work of the Vogts and their co-workers to construct a myeloarchitectonic map that is referenced to the Atlas of the Human Brain (AHB) in standard space. We developed a semi-automatic pipeline for processing and integrating the various original maps into a single coherent map. To optimize the precision of the registration between the published maps and the AHB, we augmented the maps with topographic landmarks of the brains that were originally analyzed. Registration of all maps into the AHB opened several possibilities. First, for the majority of the fields, multiple maps from different authors are available, which allows for sophisticated statistical integration, for example, unification with a label-fusion technique. Second, each field in the myeloarchitectonic surface map can be visualized on the myelin-stained cross-section of the AHB at the best possible correspondence. The features of each field can be correlated with the fiber-stained cross-sections in the AHB and with the extensive published materials from the Vogt school and, if necessary, corrected. Third, mapping to the AHB allows the relationship between fiber characteristics of the cortex and the subcortex to be examined. Fourth, the cytoarchitectonic maps from Brodmann and von Economo and Koskinas, which are also registered to the AHB, can be compared. This option allows the study of the correspondence between cyto- and myeloarchitecture in each field. Finally, by using our “stripe” technology – where any other feature registered to the same space can be directly compared owing to the linear and parallel representation of the correlated cortex segments – this map becomes part of a multidimensional co-registration platform.

## 1 Introduction

Cytoarchitecture has played a prominent role in cortical parcellation, while myeloarchitecture has not received equal recognition. The disregard to implement differences in the myeloarchitectonic features in the systematic analysis of the human cortex is surprising given the many advantages of this criterion: it was recognized early on that this method is simple and quick to carry out and produces clear results even at low magnification [1]. In addition, myeloarchitecture offers the opportunity to study the relationship between the organization of the cortex and subcortex and thus the relationship of individual cortical fields to incoming and outgoing fibers. This interdependence becomes particularly interesting for current *in vivo* magnetic resonance imaging (MRI) analyses where a myeloarchitectonic map provides an indispensable resource for tractography because variability in myelin characteristics can be “captured” through myelin profiling [2].

The neglect of myeloarchitecture is also surprising because the results of its analysis were considered to be of great value for the neuropathological assessment of pathological processes involving the cortex (“Pathomyeloarchitecture”) [3,4]. Brodmann [5] wrote in 1909, p. 272: “I openly confess, that the myeloarchitecture is more liable to produce practically useable results for many questions of human brain pathology than the cytoarchitecture.”

Following several pioneering reports pertaining to the territorial variations of the human cortex mostly by cytological criteria, Flechsig [6,7] provided an authoritative analysis of the myelogenetic differentiation (“myelogenetische Differenzierung”) of the cortex. He followed the chronological sequence of the process of medullation (myelinization) and recognized 36 fields depending on the order of the appearance of the medullary substance. He distinguished primordial, intermediate, and terminal zones and related them to extra- and intracortical (projection and association) systems. Ahead of his time, he suspected that the myelin profile has predictive power for later life.

The identification of different fields on the basis of myelination properties, i.e., of fiber characteristics in the adult brain goes back to Campbell [8,9]. Based on the study of alternating cell- and fiber-stained serial brain sections, he presented in his two books an extremely comprehensive, detailed, and consistent cortical map with a “composite representation of the distribution of the various areas” that included differences in nerve fiber concentration. He characterized about 20 different fields in the human brain [10].

In a different approach Smith [11] used the contrasts by the naked eye to distinguish about 50 areas based solely on variation in the brightness of the horizontal striations, the stripes of Baillarger, in the fresh brain, on the basis of thickness, texture, and coloration.

**Figure 1:**
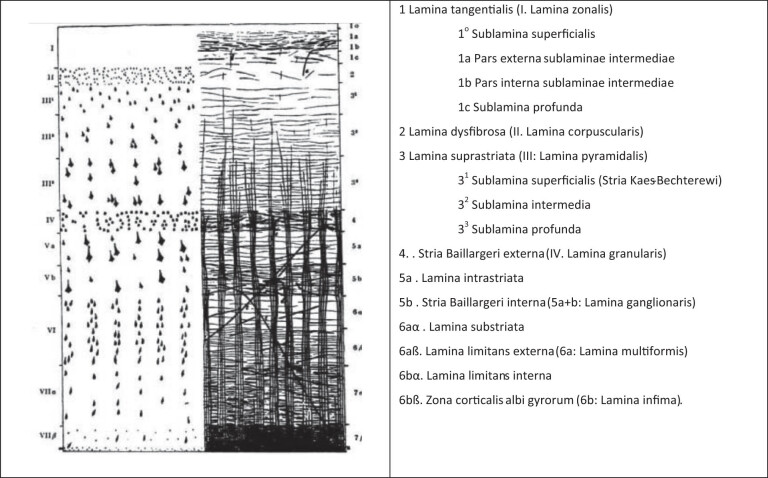
O. Vogt’s basic diagram of the cyto- and myeloarchitecture of the human isocortex [15]. Modifications of this schema characterize the different types of cortex (see Appendix 2). A comparison of both panels shows the remarkable fact that both show a close correspondence. It should be emphasized that the outer (II) of the two granular layers is particularly poor in fibers, while the inner (IV) corresponds to an accumulation of horizontal fibers, the *stria externa*. It is also noticeable that the outer horizontal (Baillarger) stripe (4) corresponds to a granular layer (IV), while the inner stripe lies in the inner sublayer of the V, which contains large pyramidal cells. Right: Vogt’s [12] terminology of the cytoarchitectonic layers (denoted with Roman numerals) and of the myeloarchitectonic layers (denoted with Arabic numerals).

A highly systematically organized cortex parcellation scheme was created by Vogt [12]. He redefined and refined Campbells’ cortical areas and added to them a very detailed and systematic parceling system. His scheme was founded on structural criteria describing the organization of the myelinated fibers. The basic variables consist of radial (longitudinal) and transverse fibers in the human neocortex in different positions, densities, and contrasts (Figure 1). His iconic figure draws attention to the fact that the laminar pattern is largely related to the cytoarchitectonic pattern. This basic concept was widely accepted and supplemented by the numerous scholars at the Vogts Institute who worked on the myeloarchitectonic analysis of the cortex in the following decades (the “Vogt-Vogt School,” “V–V school,” [13,14]).

Modifications of the scheme of the elementary fiber organization were mainly due to variations in the number, contrast, and density of the transverse and radial myelin fibers. The fact that regional myeloarchitectonic differences such as those in the Rolandic area were related to functional specificity clearly suggested that the histological differentiation of field patterns must generally correspond to functional specificity.

The differences in the local manifestations of the organization of the myelinated fibers and their relationship to the corresponding cytoarchitecture led the Vogts and their colleagues to differentiate about 185 myeloarchitectural fields over several decades [12,15–19] (Supplementary Materials 1–4). (We distinguish between the myeloarchitectonic “field” and the cytoarchitectural “area”). The peculiarities have been described using very sophisticated terminology that is difficult to understand [14,20–23] (Appendix 2). The descriptions of many fields are accompanied by numerous detailed diagrams highlighting the fine-grained myeloarchitecture. This wealth of information about the myeloarchitecture of the isocortex had not been integrated into a unified map of a complete hemisphere, as exists for the cytoarchitecture of the human cortex.

Criticism was particularly directed at the question of the accuracy and consistency of the sprawling subdivisions of the maps as well as the lack of objectification of field boundaries. In this context, it was important that Braitenberg [20,24] and Hopf [25–29] adapted photometry as an objective method to determine myelination patterns. Using this method, Hopf was able to quantify laminar differences and to describe several trends in fiber distribution, such as the decrease in myelin content from primary areas outwards to the poles and paralimbic cortex (which corresponds to the process of myelination from the central area toward the poles) [30]. However, this method was used exemplarily for delimited fields, not for demarcation, i.e., for the systematic definition of boundaries between neighboring individual fields. A consistent map of larger lobe areas was not developed.

In addition to the questioned objectivity of the method, there were arguments regarding the constancy and specificity of the delineation of the fields. They were concerned with the small number of brains examined, the consequences of inter-individual variability, and the influence of deteriorating postmortem changes in the histological specimen [31]. The inaccuracies in the characterization and limited quantification of the morphological features have several critics leading to question the implications of personal impartiality and the validity and constancy of myeloarchitectonic parcellation, or even to question the results of the assessment altogether [32,33]; for response see: [34,35]. Finally, there is the fact that the parcellation had only limited influence on the interpretation of pathological conditions and, more generally, the neurological thinking [10, p. 453]. It seems that efforts to further develop myeloarchitectonic cartography of the human brain have been abandoned and relevant questions have been delegated to animal studies that promised statistically significant results.

Renewed interest has been sparked by the demonstration of local differences in myelin content across cortical layers by improved methods [36] that can even be determined by MRI in the living human brain [37–39]. The detailed material developed by the Vogts and their numerous colleagues can therefore be an important resource for interpreting the unequal distribution of myelin in the cortex determined by MRI. Since no complete map of the cortex existed until now, but only maps of individual lobes or only delimited regions, it was a great advance that Nieuwenhuys et al. [14,21–23] managed to transfer various published maps into a probabilistic template. They integrated the various parcellations from the V–V school, including Lungwitz, Strasburger, Gerhardt, Sanides, and Hopf [26,27,40–47] into the same template (Colin) brain. Their important congregated map offered, for the first time, a three-dimensional representation of a complete hemisphere with individual, separate fields that can be registered to other brains.

The important full brain map of Nieuwenhuys et al., however, has shortcomings. Most important is the fact that the field boundaries on the maps were transferred to correlating areas of the template using a “graphical averaging process.” However, transferring the maps derived from individual brains directly to a reference brain, regardless of the underlying anatomy, is likely to result in a high degree of mispositioning, which can result in the misrepresentation of numerous fields. Registering the mosaic of individual cortical fields onto probabilistic anatomy results in topographical and histological uncertainties that can hardly be compensated for by this registration method (especially since two-thirds of the cortex, hidden in the sulcus folds, are ignored). Accordingly, the boundaries of the new map may not adequately respect the individual sulco-gyral conditions of the brains from which the maps were originally created.

Even if an acceptable agreement was achieved, important resources of the V–V school were not used, namely the existent data about the histology of each field, including the graphical representations of the cortical field structure. To exploit this wealth of information, it is necessary to project the maps onto a brain, which makes it possible to compare the myeloarchitectonic data with the corresponding features on corresponding sections.

We have taken two steps to achieve this goal. First, we considered the brains that were actually used to create the maps. This point is important because, according to most researchers, cerebral sulci contain important clues to boundary formation [5,43,44,48]. Second, we registered the results, which led to an overall map of the isocortex, to a brain whose cross-sections are available in an atlas format and therefore allow for a comparison of the myeloarchitecture of the (transformed) areas. This fact is all the more important since various authors used this brain (A58) for their studies (see Appendix 1).

To circumvent some of the above ambiguities and to achieve better agreement between the source and target maps, geometrical constrains are required to ensure that homologous areas are aligned on the maps. We therefore registered the published maps to the photographs of the brains from which these maps were derived and used the sulco-gyral anatomy as constrains for orienting and transferring the field maps.

After the existing individual maps had been adapted to the situation of the surface photos with the highest degree of accuracy, all corrected maps were registered (individually) to the Atlas of the Human Brain (AHB) that resides in the MNI/ICBM 2009b space [49]. The mapping of the myeloarchitectonic field maps in the AHB enables the different fields to be delineated on the serial sections in this atlas. With this step we have achieved two prerequisites for a substantial review and use of the results of the V–V school: First, the myelin-stained sections contained in the AHB can be directly compared with the complex variations in fiber organization defined for a given field. Second, the wealth of published illustrations and photographs depicting the histological variations of the myeloarchitectural fields can be used for the first time for verification on consistent atlas material. Furthermore, the representation of the myeloarchitectonic fields on the atlas cross sections allows the study of their correspondence with the incoming and outgoing fibers and may have relevance to tractography.

Finally, we developed a new way to represent the myeloarchitectural cortex areas along with other architectonic feature maps, which we describe as stripe representation [50]. By stretching the convoluted cortex band into a straight line, we enable the identification of multiple cortical parameters within topographically specified areas that are directly related to the position in the atlas sections. The linear representation of different features along the stripes then enables a multi-layered analysis that includes different architectural components. We believe that this representation provides the greatest possible accuracy and broadest applicability of the myeloarchitectural map at this time.

## 2 Materials and methods

### 2.1 Materials

Almost all of the brains that were used for the parcellations by the Vogts and collaborators were collected and investigated at their institute. The only notable exception was brain MB59 from the Kleist-collection that was studied by Hopf [46,47] for his elaborate segmentation of the temporal lobe. A list of the brains used for cortical partitioning is provided in Appendix 1.

The brains of the Vogt collection were documented using photographs of formalin-fixed brains before embedding in paraffin. Almost all photographs also show a metric scale, labelled gyri, and the course of the sulci. We used copies of these photographs, documented in the Vogt Collection Brain Inventory [51], to register the various authors myeloarchitectonic field maps. The reason for this approach is to correlate the published field boundaries with the actual surface topography to achieve an exact correspondence between brain morphology and brain map. For the topographically correct registration of many defined fields, knowledge of the gyro-sulcal organization is essential. The correlation with the sulci, but also with slight depressions in the cortical surface, is relevant because there is widespread agreement that field boundaries are often related to these features [52,53].

Some published maps are to varying degrees idealized and therefore difficult to align. A discrepancy between surface anatomy and the map is particularly evident in the maps by Braitenberg, Hopf, and Hopf and Vitzthum [20,46,47,54,55]). Registering such maps directly to a reference map or a template necessarily results in significant misalignment resulting in poor agreement between the histological and topographical features of the brain being evaluated and the mapped territory on the template. For an accurate transfer based on the exact gyral and sulcal properties, we used maps from other authors dealing with the corresponding fields. In the case of Hopf, we used maps from Beck [56], which show very good agreement. In the case of the pre-occipital region analyzed by Lungwitz [40], we created a 3D reconstruction based on the sections and registered this volume in the AHB template. Accordingly, we are confident that the topography depicted in the maps matches the surface representation of the photographs of the analyzed brains. We use anatomical landmarks, e.g., of the sulcal pattern, to improve registration quality.

There were additional possibilities to improve the accuracy of the agreement between the reported locations of the fields in the brains of origin and the result of our registration. First, we included maps from Vogts’ collaborators who performed cytoarchitectonic parcellation using the myeloarchitectonic parcellation scheme and corresponding terminology, implying a high degree of agreement between their cytoarchitectonic parcellation and the myeloarchitectonic parcellation. In fact, several authors concluded that cytoarchitectonic areas often coincide with those of the myeloarchitectural fields described as cyto-myeloarchitectonic coincidence [5]. Second, we used diagrams showing the field boundaries on cross sections [20,40,41]. Third, in a few cases, we were able to use photographs of cross-sections of the pertinent brains, which showed the intrasulcal position and therefore improved the accuracy of the field maps in the areas within the sulci [51]. Finally, several authors had analyzed brain A58, shown in the AHB, which is used as a template for registration in this study (Table A1). To integrate the heterogeneous materials and features, we developed a map processing workflow that allows aggregation of cortical maps in a template brain with high accuracy and multiple correction steps.

### 2.2 Methods

#### 2.2.1 Utilization of the different maps and organization of their decisive parameters

Almost all parts of the isocortex were analyzed by the Vogts and their colleagues. However, there was neither a uniform numbering system nor an unambiguous terminology for all defined fields (see Supplementary Materials 1–4). Vogt and many of his collaborators used a system that describes the relevant structural features in each field (e.g., *Regio unistriata euradiata tenuifibrosis frontalis*). Lungwitz [40] used alliterations of field features (e.g., eld, edpc; Supplementary Material 3); Hopf, on the other hand, described the fields based on the topography of the region (e.g., *Regio temporopolaris* with subregions and areas; Supplementary Material 4).

To systematically list the different fields, Nieuwenhuys [14] summarized all fields of the four main lobes in serial order. For reasons of continuity, we have adopted their tabular presentation. Unfortunately, this numbering system does not have space for the *Subregio occipitalis* (BA 18) and *Subregio striata* (BA 17) which also show myeloarchitectonic differences [57]. It also does not cover allocortical regions and many transition areas.

The creation of the myeloarchitectural map and its registration to the AHB template brain enabled the use of the unique verbal and pictorial material contained in the original reports. This material includes in addition to the characterizations of each field precise drawings or photographs of the areas examined. In Supplementary Materials 1–4, we have summarized descriptions from various authors of the V–V school and provide references to existing image material. We have relied entirely on original works. We did not intend to comment or discuss these entries in the context of modern advances in the organization and function of each area.

### 2.3 Processing of each published map into the unified reconstructed full brain map

**Figure 2:**
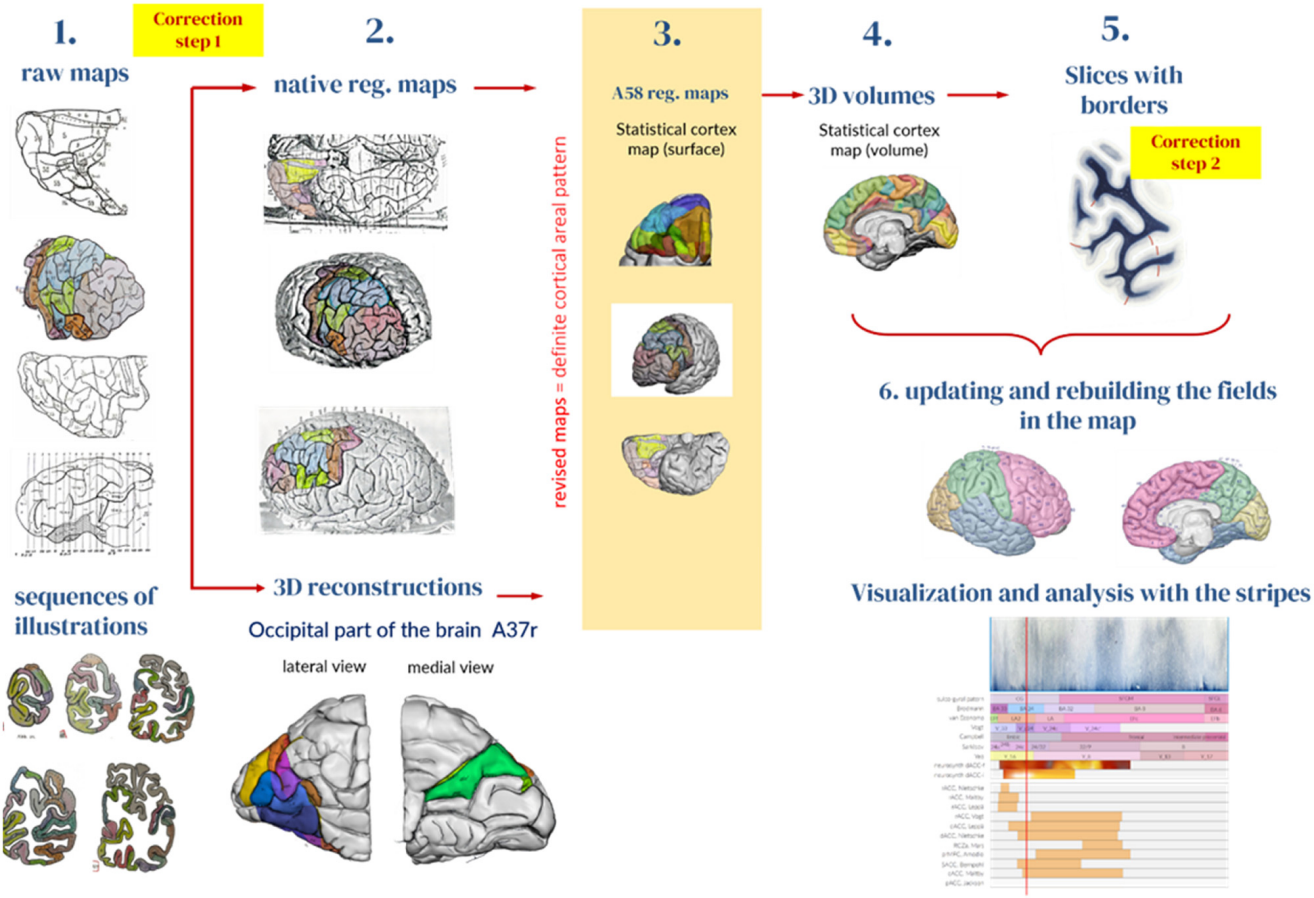
Processing steps for the construction of the composite myeloarchitectonic map. Stage 1: The maps from the literature were aggregated into a library along with the references and viewing angles of the original brain. Stage 2: *Correction step 1* aligns the topographical information with the brain surface photos either through 2D registration from the surface photos or through 3D reconstruction from the sections. The raw maps were 2D registered to the photos of the native brains (upper image row). In addition, where available (e.g., the occipital cortex of Lungwitz [40]) a 3D reconstruction of the native brain is created from the published serial sections (bottom image). Stage 3: Registration of amended maps and volumes to the AHB reference (A58). Stage 4: Projection of the fields from the registered surface maps onto the 3D reconstruction, propagating the field boundaries into the sulci and injecting the fields into the segmented cortical volume. Stage 5: Projection of the field boundaries onto the microscopic myelin-stained sections of the A58 brain and manual correction of the borders according to the local myelin structure. Stage 6: Updating and rebuilding the myelin map based on the changes created by *correction step 2*. Finally, the maps and cortical fields were visualized and analyzed using the stripe technique.

We designed a semi-automatic pipeline for efficient and accurate processing of historical myeloarchitectonic maps to incorporate them into a unified map in the AHB. The pipeline comprises six stages, each with specific functions and operations aimed at transforming the raw input maps into a single map. The proposed processing pipeline serves as a valuable framework for aggregating historic maps into a template brain (Figure 2).

#### 2.3.1 Stage 1

In the initial stage of the processing pipeline, raw maps are subjected to preprocessing operations to ensure its quality, consistency, and compatibility with subsequent analysis steps. We stored the preprocessed maps from the Vogts and collaborators (V–V and associates) together with the available photographs of the original brains [51] in a database library.

#### 2.3.2 Stage 2

In this step, we use our 2D-constrained fuzzy registration method to bring the stored maps back onto the native brain surfaces (see Appendix 4). This step entails the first map adjustment during the process of aggregation into a common space (*correction step 1*). The maps acquire explicit physiological and topographical representation that enables higher precision in the next steps of the pipeline. We employed geometrical constrains to ensure that the raw map and the image of the brain are perfectly aligned. We used selected sulci and gyri marked in the maps and in the brain images as the constrains.

In addition to using the surface anatomy of the analyzed brains, we have ensured topographically accurate registration by using sequences of illustrations that depict the distinguished fields on cross-sections provided by Lungwitz [40] for 3D reconstruction (Figure 2, Appendix 5). For the 3D reconstruction of the volume, we employed our geometric shape-constrained 3D reconstruction algorithm for serial sections [49] (see Appendix 6). This algorithm creates a specific 3D reconstruction such that it corresponds to the photograph of the source brain. A single geometric shape constrain defines a homologous structure in the source brain and in the slices that will be perfectly matched during the nonlinear part of the reconstruction process. The geometric constrains can be defined as a set of one -, two-, or three-dimensional structures (1D, 2D, or 3D constrains). Finally, some authors used the brain of the Vogt collection which is presented in the Atlas of the Human Brain, based on the brain of a 24-year-old male (A58 brain) [49]. This instance gave us the opportunity to merge those maps directly to this AHB.

#### 2.3.3 Stage 3

The revised maps are then transferred to the AHB template brain [49]. It allows for comparison of any 2D or 3D mapping data with high-resolution microscopic cell- and myelin-stained brain sections.

The transfer of the maps to a reference brain is a multi-stage procedure for the surface- and also for the volume- representations. For the surface representation, we performed the procedure that included 2D registration of the augmented maps to the surface of the A58 (see Section 2.4 and Appendix 4).

#### 2.3.4 Stage 4

From the surface representation of the maps, a volumetric segmentation map is created with the CARET software [58].

#### 2.3.5 Stage 5

In this step, the area boundaries are projected onto the slices by cutting the AHB surfaces overlaid with triangular representations of these maps. The surface cut is represented as a thick 2D ribbon and is registered to the micro-section. The borders between the fields were projected onto the microsections as lines/paths that can be studied and edited. The boundaries can be reviewed by an expert on the microscopic slices, accounting for the topography as described in the original reports, for the relationship of the borders with regard to the depth of the sulci, and for the differences in the myeloarchitecture of the fiber-stained sections represented in the ABH (Figure 2, *correction step 2*). Borderlines can be moved, added, or removed from the slice.

#### 2.3.6 Stage 6

After correcting the field borders, the delineations were projected back to the 3D surface representation by inverse registration fields generated by the surface-cut registration to the microscopic sections.

### 2.4 Registration of the various revised myeloarchitectonic field maps to the AHB template

The transfer of the semi-diagrammatic representations of the various myeloarchitectonic fields (V–V maps) from the revised published maps to a reference brain is a multi-stage procedure that is part of stage 3 of the pipeline (Figure 2, Appendices 4 and 7) for the surface and also for the volume representations. For the surface representation, we performed the following multi-component process:

- 2D registration of the augmented maps to the surface of the AHB at the appropriate viewing angle. We transfer each map to the template brain and later resolve the conflicts. During this process, we take into account the individual anatomy of the previously examined brains and create geometrical constrains that will be matched between the source and target brains. The geometrical constrains consist of defined topographical and structural reference points and lines (using the surface photographs of the brains which were used for the original reports) which serve as orientation points for the registration. These “enhanced” maps were then transferred/registered to the AHB template. This required numerous adjustments and correction routines.
- Projection of the 2D surface maps and their field boundaries onto the 3D surface of the AHB.
- In the next step, label fusion (LF) of the maps is carried out for multiple input maps. LF is a process used to combine several different labels from individual maps of the same entity (an anatomical area) into a single discrete label that can be potentially more accurate with respect to the true borders of the area. The LF generates a core of an area that is present in the majority of the maps. This core is then expanded until it abuts other area cores or the expansion is not unequivocal. The expansion speed is scaled by the concordance measure (Wallace index *W*_max_, see Appendix 3) of the area at the expansion front. Higher concordance allows for faster expansion of the front. After the LF the map is then manually redrawn.
- The areas of the maps and their boundaries are projected onto the 3D surface of the AHB. The areas and their boundaries are propagated along the curvature gradient of the surface until they reach the sulcus bottom or cross other boundaries.
- Finally, boundary conflicts and area corrections are resolved by a semi-automatic field fusion technique or manually by editing the surface in the CARET software [58].

For the registration of the 3D reconstructed volumes, we use our high-resolution constrained volumetric registration (Appendix 6) that ensures corresponding structures in the brains are almost perfectly matched.

### 2.5 Depiction and modification of the myeloarchitectonic fields on the (serial) sections of the AHB (correction step 2)

**Figure 3:**
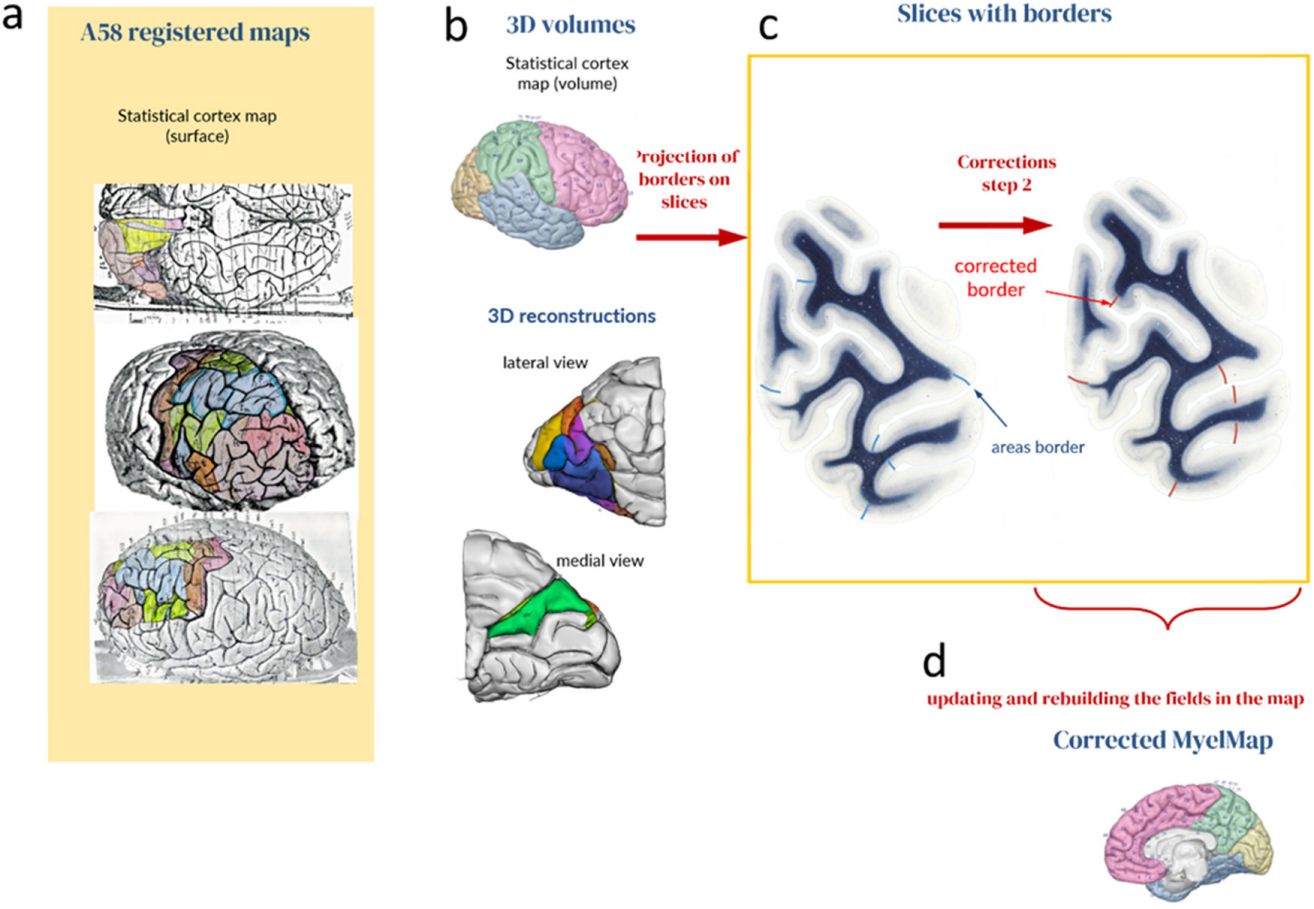
*Correction step 2*. The amended maps (a) are registered (b) to the AHB template (A58). (c) The borders of the myeloarchitectonic fields are projected from the 3D volume on the microscopic myelin-stained sections of the AHB (blue lines) and revised and modified by an expert (red lines). The myelin map is finally updated by the new borders and rebuilt in 3D (d).

Transferring the myeloarchitectonic fields (V–V maps) to the AHB allows visualization of the cortical area pattern in cross-sections (Figure 3). The amended maps (Figure 3a) are registered (Figure 3b) to the AHB template. To transfer the cortical parcellations onto the 2D myelin-stained sections, we created a coronal section through the 3D-AHB surface representation at the corresponding position. A linear contour representation of the field parcellation was created from the intersection of the surface with the coronal section plane and transferred to the histological section (Figure 3c, blue lines). This first projection of the field boundaries represents only an approximation at this stage and requires confirmation or modifications depending on a more detailed analysis of the myelin structure of the cortical ribbon. As part of an expert review, the boundaries can be modified according to the local myelin distribution. The final set of the boundaries (Figure 3c, red lines) is then projected back onto the surface and the myelin fields are updated and rebuilt into the final myelin map (Figure 3d).

### 2.6 Multidimensional cortex representation: Correlation of the myeloarchitectonic field map with the cytoarchitectonic area maps by means of the “stripe-technology”

We developed a unique query method to analyze and visualize the data and the associated database called “Cortical Stripe Representation” [49,59].

**Figure 4:**
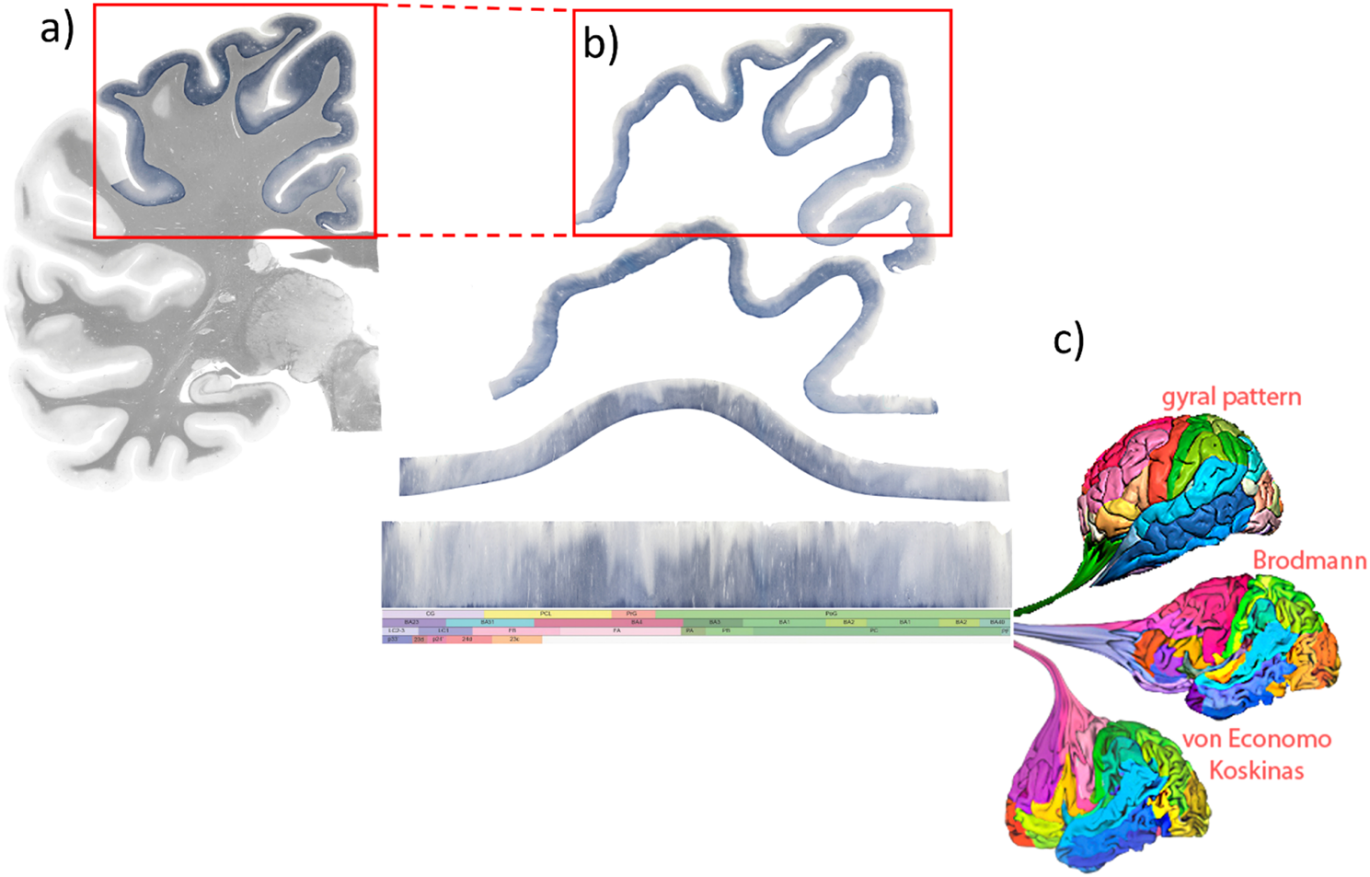
Linear representation of the cortex as “stripe“ by unfolding and flattening its variegated band. (a) A part of the cortex in the fiber-stained section is highlighted. (b) The cartoon depicts the successive deconvolution of the cortex area shown in (a) into a linear band, the “stripe.” The stripe is defined as a linear band of “unfolded” cortex representing features associated within the corresponding AHB region. The segmented and colored bands below the cortex stripe represent the sequence of the sulcal-gyral organization and functional area delineations reported by different authors at the same location. (c) The cartoon illustrates the “transfer” of information from the 3D maps to the linear cortex stripe, brought in correspondence to the AHB. The cortex ribbons derived from the different cortex maps, symbolized by the different colors on the right-sided brains, become linearly stretched and arranged in a topographically consistent manner. The stripes allow different views of the cortex to be displayed.

For this representation, the cortical myeloarchitectonic ribbons are unfolded and linearly represented as “stripes,” consistent with the interpretation of previous authors as we perceive their work (Figure 4). Since each position within these stripes has a precise relationship to the coordinates of the MRI space, delineations of cortical areas that come from other research classes can be registered.

Representing the cortical fields as stripes allows a direct comparison of the different views attributed to the homologous cortical region. Due to the linear representation of the *x*/*y* coordinates in each section (*z* coordinate), the number of synoptically presented results is not limited and can be supplemented by ROIs documented in standard space (MRI space).

This topographical precision provides the opportunity to represent not only the myeloarchitectonic characteristics of each atlas section. All other maps and features located in the same topographic location can be aligned to this stripe and compared to existing entries. The advantage of this synoptic representation is that different views of cortical parcellation are immediately apparent and different interpretations of cortical areas or inconsistencies between interpretations become clear.


**Ethical approval:** The research related to human use has been complied with all the relevant national regulations, institutional policies, and in accordance with the tenets of the Helsinki Declaration, and has been approved by the authors’ institutional review board or equivalent committee.

## 3 Results

We have compiled a large series of field maps from the Vogts and their scholars (Appendix 1) and have used this resource to create a unified map registered in the AHB. Supplementary Materials 1–4 provide insight into the data used. The final myeloarchitectonic map is the result of semi-automated processing of the maps using our six-stage processing pipeline. To improve the accuracy of the registration, we exploited topographic landmarks from the surface photographs of the brains originally analyzed. Using the AHB as a template enabled visualization of the myeloarchitectural fields on cross sections. This display option not only allows the fields to be perceived in the most likely correct position but also allows the architectural organization of each cortical field, which is usually described in detail in the reports, to be compared with the myeloarchitecture of the atlas brain.

### 3.1 Example of the processing of the orbitofrontal cortex and concordance analysis results

**Figure 5:**
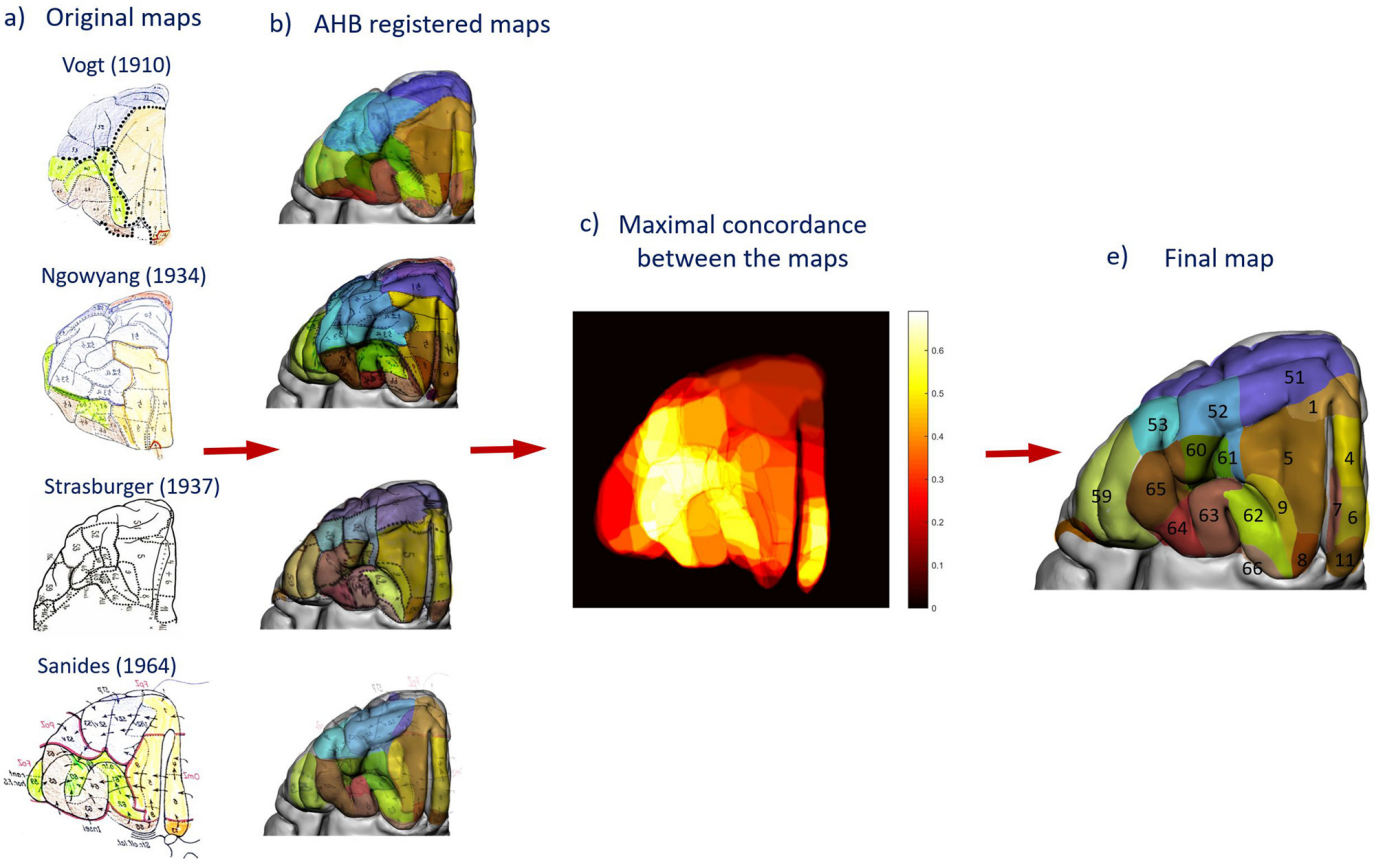
Construction of the orbitofrontal map. (a) The four original maps used for the reconstruction of the orbitofrontal map and for the concordance analysis. The raw maps are first registered to the corresponding original brains and then with stage 3 of the pipeline to the template brain AHB. As geometrical constrains, we use eight anatomical structures: frontomarginal sulcus (fms), lateral fissure horizonal part (lf-h), olfactory sulcus (olfs), straight gyrus (SG), posteromedial olfactory lobule (PMOL), anterior orbital gyrus (AORG), lateral orbital gyrus (LORG), and posterior orbital gyrus (PORG). (b) Four maps, after processing stage 3 of the pipeline, registered to the AHB. (c) The 2D concordance map of the four maps. The concordance between the maps is color-coded. Bright colors (white) indicate fields of high areal concordance, and dark colors indicate locations of low areal concordance between the maps. (d) The final reconstructed orbitofrontal map.

Since a detailed description of the entire brain processing would go beyond the scope of a single work we illustrate in Figure 5 the process of unifying and reconstructing the historical raw maps onto the AHB template using the orbitofrontal cortex as an example. The orbitofrontal cortex was divided by Vogt [15] into 21 myeloarchitectural fields [1,3–9,11,13,49–51,58–65]. The fields were assorted respecting the definitions of [41,45,54,60]. The raw maps were first registered to the corresponding original brains. We used eight anatomical structures as geometric constraints: frontomarginal sulcus, horizontal part of the lateral fissure, olfactory sulcus, straight gyrus, posteromedial olfactory lobule, anterior orbital gyrus, lateral orbital gyrus, and posterior orbital gyrus. The assembled map was then registered to the AHB using stage 3 of the pipeline in the template brain (Figure 5b).

To assess the correspondence between the maps we computed a 2D map of the adjusted maximal Wallace index *W*_max_ (Appendix 3). The area of maximal concordance between the four maps is shown by the light colors in Figure 5c. At these locations the cores of the areas are expanded with low speed and the borders are carefully redrawn and compared to selected microscopic sections of the AHB (Figure 3c). Averaging of the maps was performed using the LF algorithm [61]. Area 7 was missing from the maps of Strasburger [41] and Sanides [45] and was mandated as a core area input before the LF processing began. The final map (Figure 5d) is integrated into the 3D model map and can be further used in the “stripe analysis.”

### 3.2 The 3D reconstruction of the full brain myeloarchitecture map

For our reconstruction of the complete myeloarchitectural map of the brain, we used the published surface maps and one map derived from the volumetric reconstruction of the occipital cortex.

**Figure 6:**
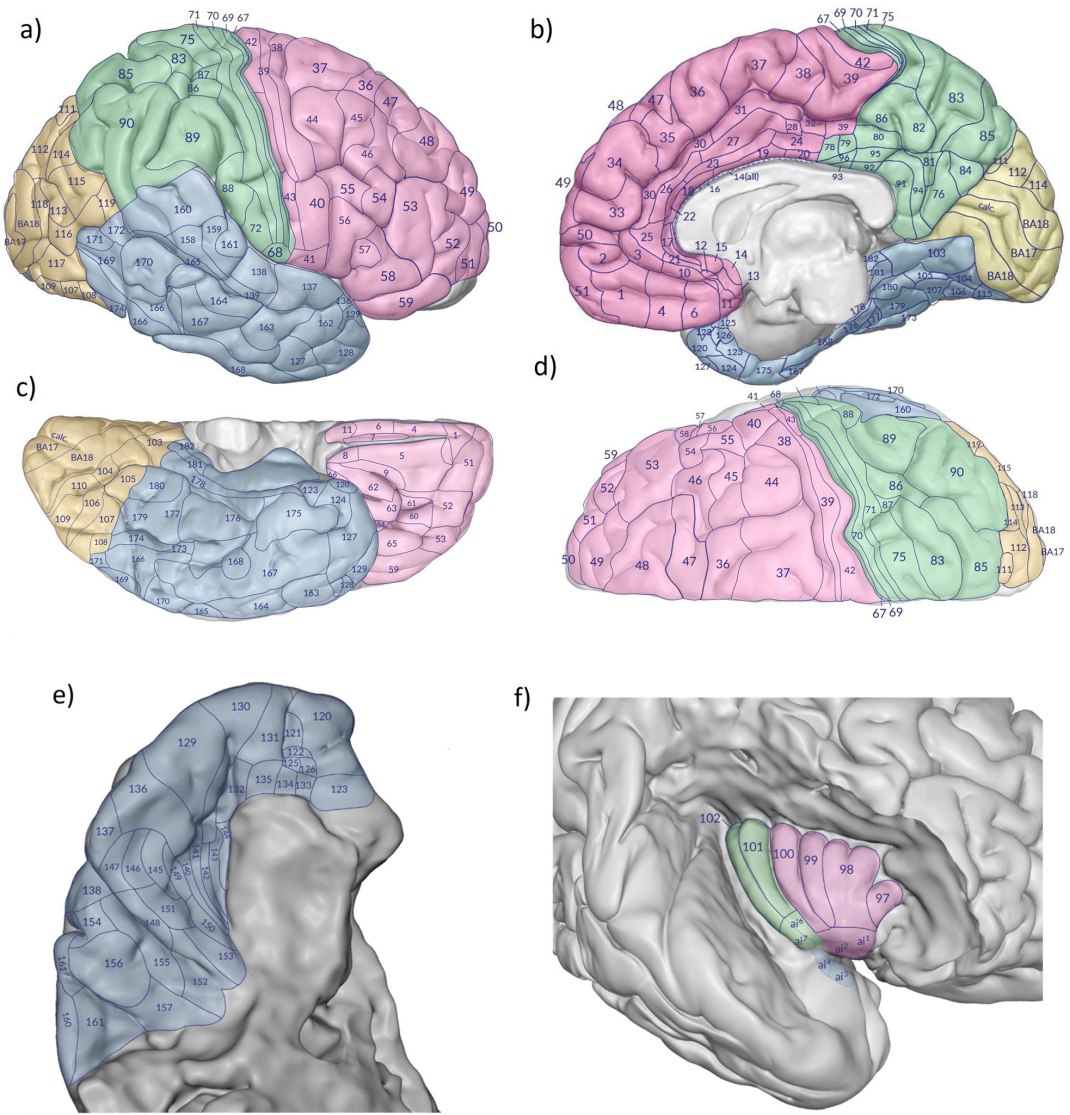
The mosaic of cortical myeloarchitectonic fields derived from the Vogt–Vogt school onto the AHB. Lateral (a), medial (b), basal (c), superior (d), supratemporal (e), and insular (f) views. This map contains 184 fields. Several researchers, however, went much further and described significantly more fields by dividing them into numerous subfields. It is therefore all the more important to precisely transfer the areas and limit the individual fields to the reference brain.

It is important to understand that the input maps are derived from many different brains and are not comparable without being standardized to a selected template. As a template, we use the brain A58 which forms the basis for the AHB [49]. The high-resolution microscopic cell- and fiber-stained brain sections in the Atlas enable comparison with any 2D or 3D mapping data. The final maps produced by the pipeline are presented in four canonical views of the AHB: lateral, medial, basal, and superior (Figure 6). The frontal, temporal, parietal, and occipital lobes are represented by different colors.

Our unified myeloarchitectural map is not yet an optimal solution to the problem of multiple maps for one lobe. One of the difficulties faced was to achieve continuous correspondence between the lobar boundary zones of the different maps. To ensure continuity in the lobar border zones, we inserted additional constraining geometric structures (gyri and sulci) at these borders and their corresponding structures in the AHB.

### 3.3 Registration to the AHB template brain. The relation to A58 allows for the representation of the myeloarchitectonic fields on cross-sections

**Figure 7:**
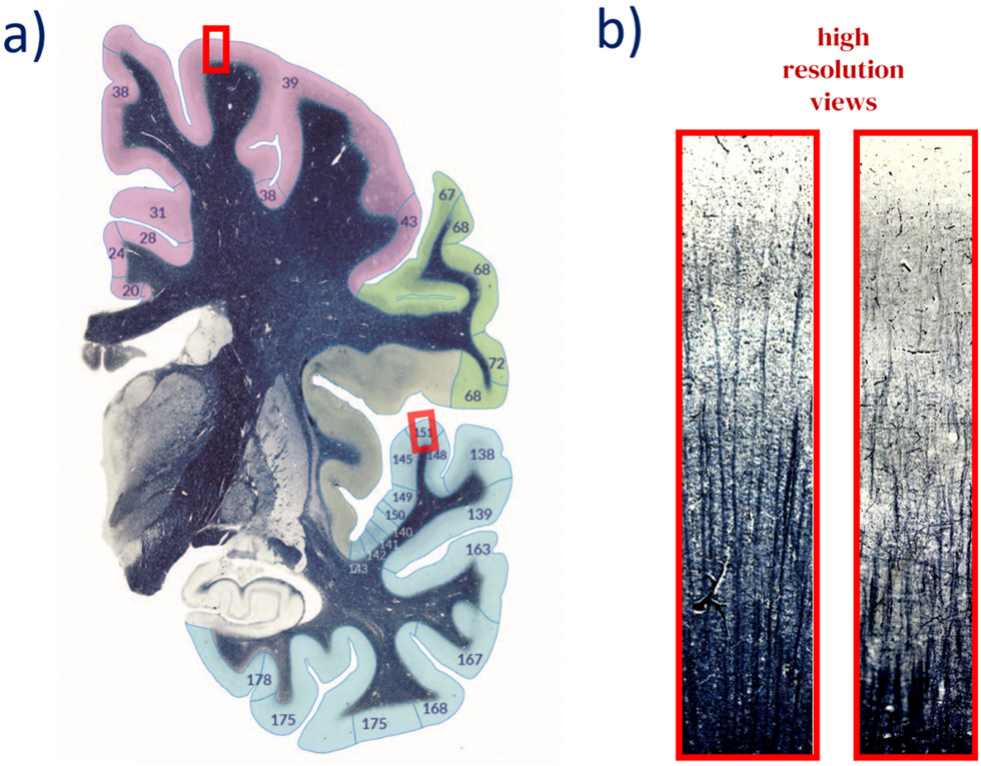
(a) Section of a plate of the AHB with myeloarchitectonic parcellation of the cortex. The delineations of each field registered to the AHB can then be confronted with graphical illustrations showing the territorial variation of the radial and tangential fibers and their verbal definitions of each field. In this figure, the two fields of the cortex are demarcated. (b) Enlarged section of the cortex profiles demarcated in (a). The myeloarchitectonic field in the frontal cortex (field 39) is characterized as follows (see Supplementary Material 1): Regio unistriata euradiata grossofibrosa frontalis; Divisio dives; Subregio propeastriata; Area propeastriata. Field characteristics: Trizonal, with larger numbers of thick singular fibers. Layer (L) 1a + b darker than in field (F)-38. L-4-6 much richer in fibers than in F-38. L-2 and L-5a poorly demarcated (relatively rich in fibers). F-39a is a relatively fiber-poor part of F-39, but it contains more fibers than F-38. A part of this field (39I) exhibits fewer basic fibers and thinner single fibers. It is unistriate, belongs to the fiber-rich category, euradiate, L4 = 5. The right-sided field (F-151) is characterized as Isocortex temporalis, Regio temporalis transversa (ttr), Subregio prima caudomedialis ttr.1cm., Area anterior ttr.1cm.a is usually on the caudal half of Ttr1, but it can also extend orally to the border of the 1st and 2nd third of Ttr1. The darkening of the Ttr1 fields reaches a maximum here. pru: prpeunistriate, i.d: internodensior-very poor in fibers; Ef: singular fibers are prominent, rrr: very dense radiary fibers.

By registering the field maps on the AHB template, the overlay can be used to display the fields not only on the brain surface but also on the cross sections of the AHB. This allows the cortical fields hidden in the sulci and fissures of the brain to be made visible. For the first time, individual myeloarchitectonic fields in all cortical sections can be directly compared with numerous detailed drawings, photographs, and detailed cortical field descriptions that accompany the original reports (Figure 7). Furthermore, each cortex field delimited in the cross-sections of the atlas also shows the subcortical structures.

### 3.4 Representation of different views of cortex parcellation on cortex “stripes”

**Figure 8:**
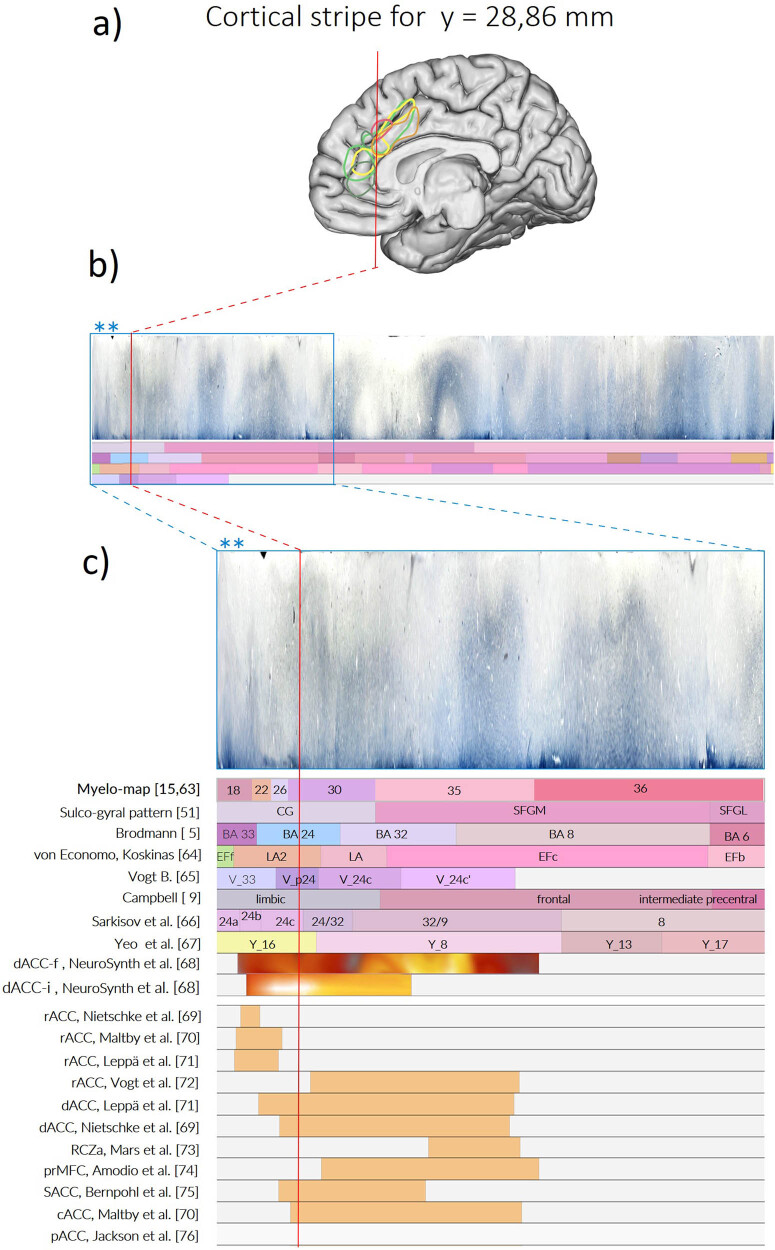
The stripe visualization and analysis of ROI in the anterior cingulate cortex (ACC). (a) The AHB4 with registered ACC activations from 13 studies. The red vertical line shows the coronal position of the analyzed stripes. (b) Stripe segments corresponding to the analyzed locations in a). A stripe segment consists of flat cortex representation (top) and associated parcellations. Here we show the canonical AHB4 stripe depicting four parcellations: sulco-gyral pattern, Brodmann map, and interpretations by von Economo and Koskinas and Vogt. (c) The segmented and partly colored parcellation bands below the cortex stripe represent the sequence of the myeloarchitectonic map, sulco-gyral organization, and functional area delineations reported at the same location by different authors. To provide an estimate of the relation between the stripe size and the histology the stripe segment depicted in (b) the small segment indicated by asterisks is shown at different magnification (boxed area with red line**). Image modified from [50].

Previously, we described our methodology for representing the cortex as a linear band in which all features occurring in the same space can be simultaneously shown [50] (Figure 8). Areal cortex maps from Brodmann [5] and von Economo and Koskinas [63] are shown in the AHB [50] that resides in the ICBM/MRI 2009b space. Using this “stripe” technology, we also registered the results of our myeloarchitectural studies.

The representation of linear cortex stripes aligns the different myeloarchitectonic fields and cytoarchitectonic areas defined by the individual authors to the common statistical coordinates of the AHB in the MRI space. This means that the defined fields can be directly compared with any other parcellation. Two applications are obvious: first, to determine the extent to which the cyto- and myeloarchitectonically segmented areas share identical fields, as has been reported (see above) and, second, to compare the different maps of the same regions published by different authors.

This parcellation strategy is not restricted to larger cortex regions. It allows to bring arbitrary *x*/*y*/*z* coordinates or any region of interest (ROI) to be brought into registry with existing entries and to correlate these with the cyto- and myeloarchitecture of the specified regions. This allows registration of results from diverse sources consistent with the correlated microanatomy of the stained sections (Figure 8). Due to the linear representation of *x*/*y* in each section (*z*-coordinate), the number of synoptically presented findings is not limited and can be supplemented by ROIs documented in standard (MRI-) space.

## 4 Discussion

The regional organization of the human cortex has been documented by many researchers using comprehensive 2D diagrams and ample details. We consider it worthwhile to bring their interpretations of the local variations into a common framework that allows the comparison of the structural features of the topological maps to be made without personal bias. With this goal, we have focused in the past on classical cytoarchitectonic maps from Brodmann [5] and von Economo and Koskinas [63] and specifically registered these in the AHB-MRI (3D-“Atlas of the Human Brain“). Consistent with these established cytoarchitectonic maps of the brain, we now add a myeloarchitectonic map of the human cortex. The relevance of such a map was already clearly foreseen by Brodmann who wrote in 19091Brodmann (1909, p. 3) “Der Markfaserbau der Hirnrinde ist eben beim Menschen vielfach …. feiner differenziert als die Zelltektonik, so dass es mit seiner Hilfe gelingt, größere einheitliche cytoarchitektonische Bezirke wieder in kleinere Felder von spezifischer Faserstruktur zu zerlegen.”: “The medullary fiber structure of the cerebral cortex is manifold in humans …. more finely differentiated than cell tectonics, so that with its help it is possible to break down larger, uniform cytoarchitectonic areas into smaller fields with a specific fiber structure.”

Nevertheless, myeloarchitectonic maps of the human cortex played only a minor role in the past. One reason for the negligence of myeloarchitectonic studies of the human cortex may have been the difficulty of creating a unified myeloarchitectural map covering the entire brain. Obviously, not all lobes of the brain in the Vogt collection were equally suitable for analysis as the staining result may be inconsistent across the brain. For example, Vogt’s [62] basic parietal lobe map was derived from three different hemispheres [A18R, A20L,R]. While almost all brains used for analysis over a period of several decades came from the Vogt Institute collection, no single brain has been analyzed consistently, nor has a complete brain map – such as Brodmann’s from the same institute – ever been completed. Instead, the analysis of the different lobes was mostly carried out on different brains (also different hemispheres) (Appendix 1). Hopf, later head of the Vogt Institute, used a brain from the Kleist collection for his very detailed studies [46,47].

Another reason could be that the intensity and contrast of the staining as well as the differentiability of the stained processes in the cortical profile have a major influence on the reliability of the parcellation. In fact, the extraction and categorization of distinguishing features of the nearly 200 fields is very difficult and the demarcation between the fields is correspondingly controversial and difficult to objectify.

Only recently have the myeloarchitectonic studies of the Vogts and their collaborators received adequate attention and application in modern research. The revival of this spectrum of their research is triggered by the interest in cortical myeloarchitecture as a characteristic feature of cortical architecture and function [76]. Moreover, advanced imaging methods, particularly tractography and molecular imaging, benefit from field- and layer-specific fiber organization related to the macro- and micro-organization of the cortex. The development of routines that allow visualization of myelin in the *in vivo* brain has initiated a revival of “myelotectonics” [20]. Indeed, in addition, myelin plays an important role in plasticity and pathology as various metrics and markers of myelin histology correlate with developmental, myelogenetic events and pathological processes. Today, the existence of profound spatial differences in the white matter properties of the cortex has been confirmed using newer imaging techniques that are sensitive to determining myelin content and distribution in the cortex and show a likely relation with global brain networks [36,77].

Since the V–V school has never produced a consistent map that covers the entire hemisphere, our model is based on several maps published over a period of about half a century by the Vogts and their scholars using a wide range of brains [13,14] (Appendix 1). Most maps show narrowly defined fields, the sophisticated definitions of which are supplemented by additional materials. In many studies, these fields were divided into further subfields that were not taken into account in our study [40,56]. On the other hand, fields were also defined more broadly and histological trends and similarities were pointed out [20,25,44]. Some maps were prepared in strict accordance with the brain surface [55] while other authors published quite idealized maps [25].

The timing was therefore right when Nieuwenhuys et al. [21] created a unified myeloarchitectonic map based on the publications of the V–V school. They registered the maps to a template brain (in standard space). We are reserved about this map because it only uses the surface features shown in the V–V maps for registration and does not take into account the subtle anatomy with almost 200 myeloarchitectural fields. The map offers little topographical certainty regarding the correct transfer to the chosen statistical brain. The differences in cortical pattern can hardly be compensated for by “graphical averaging” methods of boundary lines, especially because two-thirds of the cortex buried in the sulcal folds are ignored.

We improved the accuracy of our reconstruction by not simply putting individual elements together, but by adjusting the maps to the actual conditions based on the photographs of the brains originally evaluated. We used the information from those maps where we found a close correspondence to the surface anatomy of the photographs of the brains analyzed. This strategy is supported by observations that the boundaries between the myeloarchitectonic fields can often be associated with the sulci or even shallow depressions [44]. The transformation that places more emphasis on matching topographic landmarks increases the likelihood of correct positioning of the myeloarchitectonic fields and allowed us to improve registration.

We sought to control and minimize the inaccuracy associated with the registration between different brains and overcome the challenge of histologically correct parcellation of cortex. First, we linked the published maps to the brains from which they were developed and created a topologically corrected map taking individual anatomy into account. We transferred this overall map to the AHB (A58) using anchor points and landmarks. Since most authors emphasized that the field boundaries coincide with sulci, furrows, or even dimples [53,78] the actual surface image of the brain being evaluated played a prominent role for placing the field maps. Second, we provided the cross-sectional anatomy with the segmented fields and associated histology. Comparing the myeloarchitectonic stratification reported in the original publications with the corresponding architectural fields represented in the A58 brain helps to determine the accuracy of the registration. This aspect is facilitated by the fact that there are both explicit descriptions highlighting the typifying features and graphical representations of the field characteristics of the cortex involved. Some of these resources are available in abridged form in the Supplementary Materials. Checking the concordance between the authors’ characterization of each field and the myeloarchitectonic features presented in the atlas provides a first step toward verifiability and suitability for further study. Third, the cortical stripe representation of the cortex enables the multidimensional co-registration of alternating myeloarchitectonic maps and features from different sources mapped to the same field.

The measures described are certainly not sufficient to ensure the greatest possible agreement between the regional field registration and the template. For this reason, we have provided lists that describe the successive fields in detail, mention key myeloarchitectural features and differences from neighboring fields, and, most importantly, provide references to pictorial representations of the main features of individual fields. We anticipate that the ability to directly compare these images with sectional anatomy presented in an atlas will illustrate the precision achieved for each myeloarchitectonic field.

We did not make any special effort to test the correspondence between the positions of individual fields in the respective analyzed (historical) brains and our parcellation in the statistical brain, nor the differences from the map of Nieuwenhuys et al. [21], as revisions can be done much more precisely by comparing the detailed definitions of the field properties with the myeloarchitectonic features of the correlated cortical region in both hemispheres of the same brain (the right hemisphere in the AHB; the left hemisphere in the MRX-brain) [79].

A more effective and powerful way to test the validity of our parceling is using our Multi-Level Stripe technology (Figure 8). This technology makes it possible to synoptically bring together different cortex maps in the same space in order to specify the local peculiarities in as much detail as possible and make them comparable. The correlation between the cytoarchitectural maps of Brodmann and of von Economo and Koskinas, already mapped in our atlas, and the new myeloarchitectonic map will show not only the coincidences but also spatial differences and thus evaluate the consistency of our parcellation. This comparison is important because several authors have pointed out the high concordance between cytoarchitectural and myeloarchitectural fields. Some authors even used definitions of myeloarchitectonic fields to describe the features of the corresponding cytoarchitecture [52,60,80–83] (Table A1). We believe that the availability of structural and histological data combined with newer imaging techniques may provide additional information about the organization of underlying networks and potentially the localization of behavioral or cognitive functions. With this strategy, we aim for a multilayered organization of multiple features at a specific location.

When using multi-level stripe technology, current studies can of course also be taken into account. However, since the current study focuses on registering the historical maps of the V–V school, it was not our aim to discuss such maps with reference to recent developments regarding the myeloarchitectonic parcellation of the human cortex. Accordingly, we did not consider the results of alternative approaches [84,85] since our concept is based on the best possible anatomical match between the V–V school maps and the statistical brain. We also did not address the outstanding work of Sewards [86]. He used Hopf’s maps of the human temporal lobe to combine them with modern concepts of cortical functionality. In our opinion, his contribution outlines the perspective of how these maps and accompanying materials should be used in the future.

### 4.1 Perspective

In addition to the development of an overall myeloarchitectonic map – taking into account the individual anatomy and the registration on a statistical brain (A58) – we have achieved three conceptually important aspects: first, the representation of the myeloarchitectural fields on cross-sections of the atlas brain, second, the relationship between the original characterization of each field and the detailed characteristics of the correlate in the cross-sections of the AHB, and third, the availability of the map for multidimensional co-registration (multilevel stripe representation) of the cortex.

### 4.2 Registration of various (individual) cortex maps to the AHB and comparison with MRI-myelo-map

The mapping of each topographically determined feature in the cortex to the AHB space can be directly compared to the myeloarchitectonic features attributed to the same location. Thanks to stripe technology, the results of such evaluations can be read directly thanks to the linear and parallel display of the stripe segments.

### 4.3 Correlation of the myeloarchitectonic stripe pattern with results from tractography

The organization of myeloarchitectonic fields reflects the diversified connectivity of functional networks. Field maps related to their detailed subcortical anatomy, included in the AHB, can serve as a useful resource for interpreting connectivity maps. Fields can be used as “seed” and “end” regions for tractography [87].

### 4.4 Correlation of the myeloarchitectonic stripe pattern with results from myelin imaging

The use of multimodal and laminar MRI has demonstrated the feasibility of identifying differences in myelin content in cortical areas of the living human brain. Areas that were previously parcellated in postmortem brains can now be specifically targeted and examined using MRI. Exemplary are the findings of Glasser et al. [88] who used an automated method based on a supervised machine learning classifier and delineated 180 areas per hemisphere bounded by changes in cortical topography, architecture, and functional connectivity.

### 4.5 Vogts classification schema of the cortical myeloarchitecture

Enthusiastic as a collector of bumblebees and interested in genetic variability, it is no surprise that Vogts [89] combination and arrangement of cortical characteristics is extremely categorical and detailed. In the Supplementary Materials (and tables from Lungwitz and Hopf therein), we provide examples that illustrate the rational of this classification scheme (and are reminiscent of Raimondo Llull’s combinatorial principles [90]). It seems worth considering whether a contemporary version of this classification concept could serve as a basis for an expanded terminology of the cerebral cortex.

## Supplementary Material

Supplementary material

## Figures and Tables

**Figure A1:**
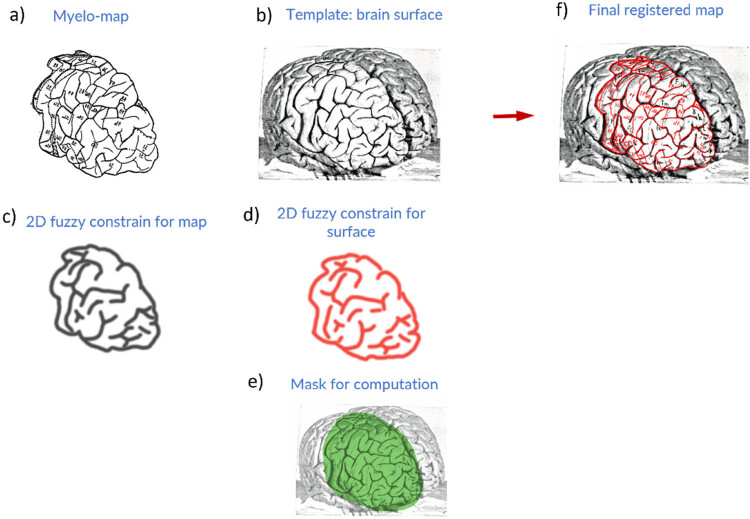
The 2D fuzzy image registration inputs and the final registered myelo-map. (a) the myeloarchitectonic map that will be registered to the corresponding brain surface (b). The filtered distance lines for significant sulci severs as 2D fuzzy constrains for map (c) and surface (d) during the registration and exert additional forces to align the map with the surface. The computation minimized the distance measures only in the masked green area (e). The final registered map overlayed onto the brain surface (f).

**Figure A2:**
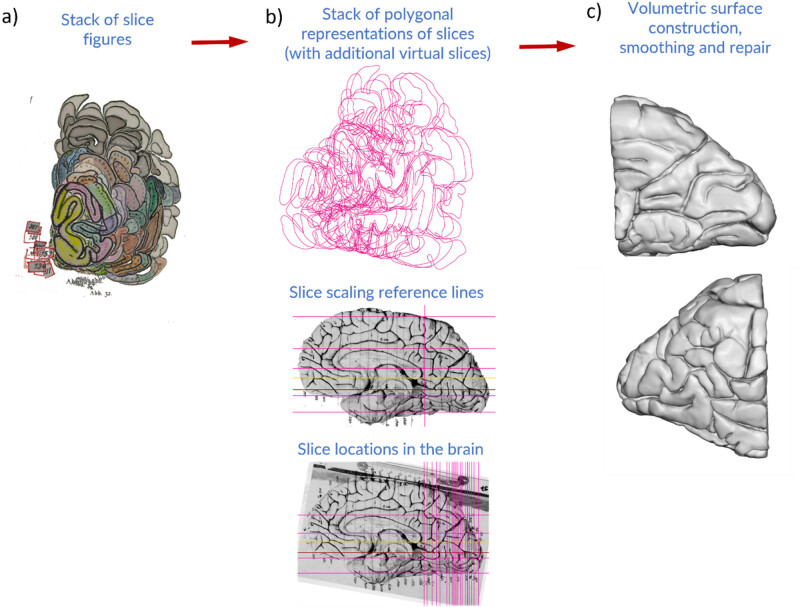
The 3D reconstruction of brain volume from stack of figures. (a) The stack of figures is extracted and vectorized. The polygonal representation of the slices (b, top) is scaled according to the reference lines (middle) at the slice position on the surface photograph (c, bottom). (c) The final surface reconstruction is smoothed and repaired.

**Figure A3:**
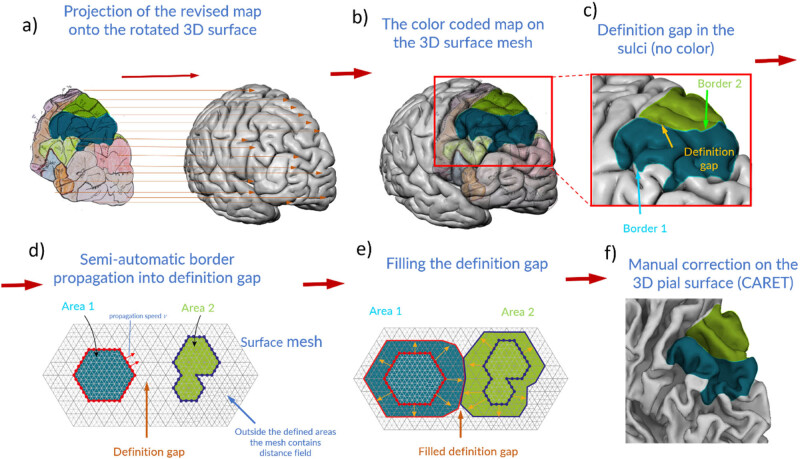
The border propagation algorithm uses the projected maps on the triangular brain surface (a). The areas are represented as colored (numbered) triangles in the mesh (b). The deep parts of the brain are out of the map projection and contain no area definition: definition gap (c). The void (undefined) part of the surface mesh (d, gray triangles) is filled by the expansion of the area borders into the void (e). The final area borders can be manually edited and revised in the CARET software [58] on the skeletonized or inflated surface mesh (f).

## Appendix 1 Resources for creating the composite myeloarchitectonic map

**Table A1:** Resources for the generation of the myeloarchitectonic map

Frontal lobe	Brains used	m	l	b	o	d,dl	op	#
Knauer [91] *(Broca)*	9 hemispheres							
Vogt [15]	**A18R**	**+**	**+**		**+**	**+**		
Riegele [92] *(Broca)*								
Ngowyang [53,60] *cy*	A49L, **A61L**	**+**	**+**	**+**	**+**	**+**	**+**	
Kreht [81] *cy*	**A58L, A61L,** A64L**, A65L**		**+**	**+**				
Kreht [82] *(Broca) cy*	**A58, A61,** A64**, A65**, A118, A126(L)							
Strasburger [41,93]	**A39R**	**+**	**+**		**+**	**+**		**#**
Strasburger [42] *(Broca)*	**A20, A22, A27, A34, A38, A39 (L,R)**		**+**					
Beck [80] E (1949) *cy*	9 hemispheres; 7 brains				**+**			
Braitenberg [20]	not specified	**+**	**+**		**+**			
Hopf [27,54]	**A18, A39 R**	**+**	**+**	**+**		**+**		
Sanides [44,45]	**A58, A43, A63**	**+**	**+**		**+**	**+**		
**Parietal lobe**								
Vogt [62]	**A18R, A20L,R**	**+**	**+**			**+**	**+**	
Gerhardt [43]	**A61L**		**+**			**dl**		
Batsch [94]	**A37L, A61L**, + multiple hem	**+**	**+**			**dl**	**+**	**#**
Hopf and Vitzthum [55]	**A37L**	**+**	**+**			**+**	**+**	
Schulze [95] iPL *cy*	**A58, A65, EL13**							
Hopf [28,29]	8 hemispheres, **A58**							
**Temporal lobe**								
Beck [96] sdTL	**A27L,** MB54, 50, 55 (Kleist Collection)					**dl**		
Beck [56,97] sdTL.	**A37LR**					**dl**		
Sgοninα [98]	**A61L, A66 R,** Th5, **A20 L, A58, A43**							
Hopf [26,46,47]	MB59L (Kleist Collection), **A58**		**+**	**+**		**sf TL**		**#**
**Occipital lobe**								
Vogt [99] *cy*								
Beck [97] *cy*								
Ngowyang [60] *cy*	**A61 L**	**+**				**+**		**#**
Lungwitz [40]	**A37L,R**	**+**	**+**	**+**				**#**
Heinze [100,101] *cy*	**A58LR,A49LR,EL4,7,8,9,10,11**							
**Insula**								
Brockhaus [102]	**A18L,A39L,A40L,A61L,A65L,A66L**							**#**

The first column provides the studies, separate for each lobe with the authors sorted according publication time. The studies from [97] Beck (1930) and [92] Riegele (1931) were not available. (Broca = analysis of the wider Broca area; *cy* = cytoarchitectonic analysis; F = field). The alphanumeric designations in the second column designate the brains used in the listed articles. Bold characters indicate those brains where the surface views of the formalin fixed brains were available (L = left hemisphere; R = right hemisphere). The right sided columns indicate the angle of view of the graphic representation of the individual brain maps in the corresponding articles (m = medial; l = lateral; b = basal; o = orbital; d = dorsal; dl = dorsolateral; op = opercular view; iPL = inferior parietal lobule; sdTL = superodorsal temporal lobe), + graphic representations available. In the outermost column is indicated whether serial sections with field maps (#) are available. The cyto- and myeloarchitecture of the cortex has been reviewed by Rose [103,104].

## A1 Myeloarchitectonic Terms

Criteria for assessing the myeloarchitectural cortex fields are essentially the width of cortex layers, the intensity of staining, and the contrast between the layers and sublayers. Each field characteristic comprises the content, caliber, character, and orientation of myelinated fibers (Bf/Gf = basic or ground fibers; Ff = fiber plexus; Ef singular fibers; Hf = horizontally oriented fibers; If = individual fibers; Tf = tangential fiber). Special attention is paid (1) to the different behavior of the two Baillarger stripes (B1: outer = Gennari stripe) (B2: inner stripe); (2) to the presence or absence of a Kaes-Bechterew stripe, and (3) to the average length of the radial fibers.

As a template for the judgment of the field characteristics serves Vogt’s [15] basic scheme, which is reproduced in its original version (Figure 1).

### A1.1 Basic scheme

In this scheme, the **1st layer**, *lamina tangentialis*, can be divided into four sub-layers or zones (1°, 1a, 1b, 1c); when fully developed: quadrizonal type.

1-1°, the outermost *sublamina superficialis* forms a narrow border zone with sparse, very fine fibers, which are only visible under high magnification.

1-1a, the subsequent pars externa *sublaminae intermediae* shows fairly densely arranged, horizontally directed, delicate fibers (basic or ground fibers, Bf).

1-1b, the pars interna *sublaminae intermediae* shows almost as many basic fibers as la, but also has a larger number of denser individual fibers (If).

1-1c, the *sublamina profunda* has significantly fewer fibers than 1b and often consists of only bundles of basic fibers. (The usually evenly built 1 c sometimes shows a loosening of the fibers in its outer part (biprofundo-zonal form)).

**L-2**, *lamina dysfibrosa* usually lacks any single fibers and this scarcity renders this layer indistinct.

**L-3**, the *lamina suprastriata* is divided into 3 sublayers, similar to the cytoarchitectonic layer III.

In the uppermost sub-layer (3-1) there can be a large number of individual fibers that make this sub-layer visible as a stripe by the naked eye (stria Kaes-Bechterewi).

The *sublamina intermedia* (3-2) has both more horizontal fibers and more terminal splitting of the radii than 3-1.

The *sublamina profunda* (3-3) is richest in fibers. It contains many horizontal fibers and also more terminal branches of radii than 3-1.

The **4th–6th layers** are discussed together due to many common structural modifications. In the basic scheme, both bands (“stripes” [105]) of Baillarger are pronounced and clearly separated from each other by a light intermediate layer. This case corresponds in Vogts basic scheme to the *bistriate* type. The layer between both stripes (layer 5a) is called *intrastriate* lamina, and the layer below the inner stripe (layer 6a) is called *substriate* lamina. Layers 1, 2, and 3 lying above the striae have no special names. Only in those few cases where a horizontal fiber accumulation stands out at the upper edge of L-3, the Kaes-Bechterew-stripe is described.

**L-4** (*stria Baillargeri externa*) forms a very dark stripe due to a dense accumulation of fine basic fibers and the appearance of thicker individual fibers.

**L-5a** (*lamina intrastriata*) corresponds to the outer sublayer of the lamina ganglionaris (Va). It is significantly lighter than L-4 due to a reduction in the GF and a decrease in the Ef.

**L-5b** (*stria Baillargeri interna*).

**L-6aα** (*lamina substriata*) is light-colored due to a reduction in the fiber feltwork. The 6aβ (lamina limitans externa) is very fibrous.

**L-6bα** (lamina limitans interna) and especially the *zona corticalis albi gyrorum* (6bβ) are significantly richer in fibers than 6a. They can hardly be distinguished from the underlying medulla on fiber preparations because of its abundance of fibers, 6bβ no longer at all. They coincide with the lamina infima (VII) of the cell image.

#### A1.1.1 Modifications of the basic scheme

**L-1: (** ***lamina tangentialis***).

Modifications of the basic (quadrizonal) type depend from

- **Contrast**:
  **Trizonal** type: 1a which few Bf and no Sf can fully adopt the architecture of 1b; the sub-layers 1a and 1b can therefore not be separated (1°, 1a + 1b, 1c). The difference between 1a + b and 1c is mainly due to the greater number of either Bf or If or both in 1a + b. The trizonal type is very common. Variations depending from either minor or stronger likeness to the trizonal type are the **subquadrizonal** or **propetrizonal** types, respectively. The latter (propetrizonal) type is also characterized by a 1a with fewer fibers.
  **Bizonal** type: the sublayers 1a + 1b + 1c (= 1a–c) are not distinguished. Individual fibers penetrate into 1a + b so that finally a similar layer 1a + b + c (= 1a–c) arises.
- **Fiber type**:
  **Eufasciate** type: a considerable difference exists between the (typically) greater amount of fiber in 1a + b compared to 1c.
  **Dysfasciate** type: no difference exists between 1a + b compared to 1.
- **Width** of the sublayers:

**Tenui**-, **medio**-, **latofasciate** type signify that 1a + b can either be narrower than L-1c, or just as wide or wider.

**L-2 (** ***lamina dysfibrosa***).

If the individual fibers in L-2 are absent, one speaks of the eutangential type; if they are present, of the ultratangential type. Usually, there is a very great scarcity in basic fibers in L-2 (eucingulate type), and only rarely is L-2 so rich in fibers that it cannot be distinguished from L-1c and L-3 (dyscingular type).

**L-3 (** ***lamina suprastriata***).

**L-4-6.**

The behavior of the Baillarger (B)-stripes to each other is valued with respect to:

- **fiber density**
  - *Aequodensus* B1 equal to B2,
  - *Externodensior* B1 to B2 denser (more fibers are present in the outer layers, L-4), and
  - *Internodensior* B1 to B2 less dense (fiber content is greater in the inner layer (L-5-2); and
- **Width** of each stripe
  - *Externolatior*: the outer stripe is significantly wider than the other without differing in fiber content,
  - *Internolatior*: the outer stripe is significantly wider,
- Differences in **fiber** characteristics in the Baillarger’s stripes,
  - *Tenuifibrosus* type, and
  - *Grossofibrosus* type.

**Unitostriate** type: the fiber density in both stripes can be particularly strong, but the intermediate layer (Lamina intrastriata, L-5a) also contains relatively many medullary fibers, so that the two stripes appear more or less fused.

**Propeastriate** type: the two B-stripes become uniformly dense and indistinct.

**Astriate** type: the two stripes and eventually are not distinguishable altogether.

**Aequodense** type: the fiber content of layers 4 and 5-2 is of the same level.

**Subconjuncto**- or **conjuncto-striate** type: the fiber content increases only in 5-1.

**Propeunistriate** type: the fiber content increases only in L-6-1.

**Unistriate** type: the substriate layer is significantly richer in fibers than the intrastriate.

In the basic scheme [15], the **radial fibers** (radii) end in the middle of the 3rd cortical layer. This situation was described as euradiate type in contrast to the supraradiate type where the terminals of the radii extend to the 1st layer and the infraradiate type, in which the radial bundles end in the middle of the 5th layer.

The radii differ not only in length but also in width, number, and caliber:

lato-, medio-, and tenui-radial types describe the **width** of the radii,

denso, modico, and sparso radiates refer to the numbers of radii,

grosso-, aliquanto and finoradiate types denote the fiber caliber.

## Appendix 3 Concordance Measure

An optimal area *level concordance measure* should be one if two areas from different maps are perfectly mutually predictable and zero if there is no predictability between the area clusters. We analyze these group-level correspondences with measures based on adjusted Wallace index [106,107]. The Wallace index *W*_A→B_ quantifies directional correspondence A→B between two clusters of areas without penalizing areas that have pure subset configuration. Given two area groups A and B, Wallace index *W*_A→B_ between group A and group B is the probability that two voxels are classified together in one area in group B knowing that they were classified together in one area in group A. The Wallace coefficient (*W*) directly indicates the agreement between partitions and therefore can be easily interpreted.

From the two directional Wallace values, we derive the adjusted maximal Wallace index *W*_max_ = max(*W*_B→A_, *W*_A→B)_. The scalar-valued maximal Wallace index *W*_max_ for two parcellations takes values between 0 and 1 and emphasizes the highest mutual predictability of two areas, with 1 denoting perfect mutual predictability of one area group from the other.

## Appendix 4 2D-Constrained Fuzzy Registration

The 2D-constrained fuzzy registration is based on the nonlinear multigrid registration for multi-modal images [108,109]. It uses an elastic multigrid method to register an image or a volume to a reference template and allows for the introduction of structural constrains. We adapted the method to the registration of surface maps (Figure A1a) by introducing fuzzy representation of the cortical folding as filtered distance lines (Figure A1c and d). These constrains serve as additional force during the registration and the algorithm minimizes the distance between the constrains in the moving image (the map) and the template (brain surface). These additional forces ensure that the selected sulci lines almost perfectly fit between the map and the cortical surface image. The algorithm uses not the whole image of the surface but only a portion that corresponds to the extent of the myelo-map.

The algorithm works in two stages:

First, the myelo-map is rigid and affine registered to the target brain surface with standard 0.5 mm resolution. We use the standard resolution (0.5 mm) registration with increasing degrees of freedom (DOF), rigid (6 DOF), and affine (12 DOF). Each registration type uses a coarse-to-fine approach and smooth-to-sharp refinement. The distance measure used for minimization is a weighted combination of the mutual information [110] and parallelity of gradients [109].

The first stage is followed by a non-linear registration (second stage) between the myelo-map and the brain surface. This stage uses the myelo-map and the constrains as an input.

The final output of the algorithm is a map that matches the important sulci of the target brain surface (Figure A1f).

## Appendix 5 3D-Constrained Volumetric Registration

The 3D-constrained registration procedure [50] is a multi-stage process that is based on the nonlinear multigrid registration for multi-modal images developed by [108,109] and extended with a diffeomorphic registration method [111].

During the preprocessing stage, the image intensities are bias field corrected [112] and normalized [113].

The 3D representation of the brain (moving brain) is rigid and affine registered to the target brain (template, volume of A58) with standard 1 mm resolution. We use the standard resolution (1 mm) atlas registration with increasing DOF, rigid (6 DOF), and affine (12 DOF). Each registration type uses a threefold coarse-to-fine approach and four-fold smooth-to-sharp refinement. The extend of the computation is constrained to the size of the moving brain with some extra space around in the target space.

The rigid and affine registration procedure is followed by a non-linear diffeomorphic registration between the moving brain and the A58.

- The algorithms applied shall provide optimal registration accuracy and robustness for the registration of the variable topography and the small-sized components moving brain. Because the description details of the deformation fields cannot sometimes sufficiently adjust small structures at standard 1 mm MR resolution, this high-resolution stage with the 0.25 resolution is executed. The high-resolution stage is computed locally in an ROI around the moving brain.
- In this high-resolution stage, the results of stage 1 are locally optimized with a sequence of multi-modal distance measures to match control volumes of the moving brain-related control volumes between the age group templates and the patient brain.

## Appendix 6 3D Reconstruction Method

For the 3D reconstruction of the AHB, we employed our geometric shape-constrained 3D reconstruction algorithm for serial sections [49,50]. This algorithm creates a specific reconstruction such that it corresponds to the surface of the target brain. A single geometric shape constrain defines a homologous structure in the source slices and in the photograph of the source brain that will be perfectly matched during the nonlinear part of the reconstruction process. The geometric constrains can be defined as a set of one-, two-, or three-dimensional structures (1D, 2D, or 3D constrains). Here, we use positions of the sulci and the outer shape of the brain as constrains. During the reconstruction, the slice will be deformed in a way that will match the form of the sulci in the photograph.

The stack of brain section figures (Figure A2a) was vectorized into polygonal representation (Figure A2b). When the gaps between the slices were too large, we inserted virtual slices that followed the form of the neighboring slices. The corresponding positions on the source brain photo were determined and the corresponding locations of the sulci and the outer borders of the brain were stored as a small slice ribbon along the section (Figure A2b bottom). From the polygonal representation, we created a volume stack with coronal slices representing the white and gray matter of the brain and the ventricles.

The slices in the volume underwent further optimization by slice-to-slice alignment of the stack. The alignment process was performed in two stages: the linear and the nonlinear stage. During the first stage, we created a rigid slice-to-slice registered slice stack with the control of the slice extension by a small ribbon extracted from the surfaces of the corresponding brain lateral, medial, and top or bottom view of the target brain. In this step, 1D and 2D geometric constrains were defined. The set of 1D landmarks consisted of the sulcal crossing of the corresponding slice line and for the 2D volume constrains, we used the overall 2D shape of the brain, and the flow of the gyri.

The slice-to-slice registration was followed by a global nonlinear stage. Each slice was nonlinearly registered to the neighboring slices. Simultaneously, the geometric constrains for each slice were aligned. After the initial match, a high-resolution iterative optimization procedure was applied to ensure a perfect match between the constraining structures and the structures of the target brain. We used an optimized sequence of two multimodal measures during this high-resolution step (mutual information and the parallelism of the gradients).

The surfaces of the cortex, the white matter, and ventricles were created from the aligned stack by the MATLAB isosurface function. The final surface meshes were smoothed and possible defects were repaired (Figure A2c).

## Appendix 7 Boundary Propagation

The area/field boundary propagation is a semiautomatic algorithm that expands defined areas on a triangulated mesh into undefined portions of the mesh. To bring the area definition from 2D to 3D we use a projection of the 2D map onto the 3D brain surface mesh (Figure A3a). The projected maps in this work contain undefined parts predominantly in the sulci (Figure A3c, the definition gap). To fill the gyri in a controlled and continuous way, we compute a distance field in the undefined part of the mesh. The distance field assigns each vertex in the mesh the distance to the closest defined area. The gradient of this field points in the direction perpendicular to the border of the area and is used as the direction of the border propagation (Figure A3d). The expansion of the area is performed by the movement of the area border over the triangular mesh. The border is defined as a polygonal line on the mesh. Each vertex is contained in a triangle or on the edge of a triangle. The vertices are moved along the distance field gradient with the velocity \[\overrightarrow{v}]\] (Figure A3d). In each step, the new location of the vertex border is tested if it is in the void triangle or if it is inside of the defined area. In the second case, the vertex is positioned border of the neighboring area and its velocity is set to zero.

This algorithm can also be used for a 2D flat mesh to expand the core areas after the LF processing of the maps. In this case, each triangle in the void part of the mesh contains additional information on which areas were presented in the overlayed maps and how many maps contained the selected areas. For this application, the movement of the area borders is defined by the gradient of the distance field and the stopping criteria are the border of the other area and/or the probability of the area in the new location is not the majority in the array map. After this automatic part of the algorithm, the not filled parts and the resulting mesh can be manually corrected and revised in mesh editing software.

## References

[1] Flechsig P. Einige Bemerkungen über die Untersuchungsmethoden der Grosshirnrinde, insbesondere des Menschen. Abdruck aus den Berichten der math-phys Klasse der Königl Sächs Gesellschaft der Wissensch zu Leipzig (Sitzung vom 11 Januar 1904). Berlin: Walter De Gruyter; 1904. 10.1515/9783112432464.

[2] Paquola C, Hong S-J. The Potential of myelin-sensitive imaging: Redefining spatiotemporal patterns of myeloarchitecture. Biol Psychiatry. 2022;93:442–54. 10.1016/j.biopsych.2022.08.031. 36481065

[3] Kleist K. Sensorische Aphasien und Amusien auf myeloarchitektonischer Grundlage. Drei Vorträge. Stuttgart: G. Thieme Verlag; 1959.

[4] Kleist K. Myeloarchitektonik und Pathologie des Scheitellappens. Arch für Psychiatrie und Z f d ges Neurologie. 1960;200:329–38. 10.1007/BF00345281. 14409775

[5] Brodmann K. Vergleichende Lokalisationslehre der Grosshirnrinde in ihren Prinzipien dargestellt auf Grund des Zellenbaues. Leipzig: Barth; 1909.

[6] Flechsig P. Gehirn und Seele. Rede, gehalten am 31. Okt. 1894 in der Universitätskirche zu Leipzig. 2. Aufl. Leipzig, Veit & Co. ; 1896.

[7] Flechsig P. Developmental (myelogenetic) localisation of the cerebral cortex in the human subject. Lancet. 1901;158(4077):1027–1030. 10.1016/S0140-6736(01)01429-5. https://www.sciencedirect.com/science/article/pii/S0140673601014295.

[8] Campbell AW. Histological studies on the localisation of cerebral function. J Ment Sci. 1904;50(211):651–62. 10.1192/bjp.50.211.651.

[9] Campbell AW. Histological studies an the localization of cerebral function. Cambridge: University Press; 1905.

[10] Clarke E, O’Malley CD. The human brain and spinal cord. a historical study illustrated by writings from antiquity to the twentieth century. Berkeley: Univ. California Press; 1968.

[11] Smith GE. A new topographical survey of the human cerebral cortex, being an account of the distribution of the anatomically distinct cortical areas and their relationship to the cerebral sulci. J Anat Physiol. 1907;41(4):237–54. PMID: 17232738; PMCID: PMC1289123. PMC1289123 17232738

[12] Vogt O. Zur anatomischen Gliederung des Cortex cerebri. J Psychol Neurol (Lpz). 1903;2:160–80.

[13] Judaš M, Cepanec M. Oskar Vogt: The first myeloarchitectoic map of the human frontal cortex. Transl Neurosci. 2010;1(1):72–94.

[14] Nieuwenhuys R. The myeloarchitectonic studies on the human cerebral cortex of the Vogt–Vogt school, and their significance for the interpretation of functional neuroimaging data. Brain Struct Funct. 2013;218:303–52. 10.1007/s00429-012-0460-z. 23076375

[15] Vogt O. Die myeloarchitektonische Felderung des menschlichen Stirnhirns. J Psychol Neurol (Lpz). 1910;15:221–32. http://www.thehumanbrain.info/database/db_references/vogt_stirnhirn_1910.pdf.

[16] Vogt C, Vogt O. Allgemeine Ergebnisse unserer Hirnforschung. J Psychol Neurol (Lpz) Ergänzungsheft 1, JA Barth. 1919;25:273–462.

[17] Vogt C, Vogt O. Die vergleichend-architektonische und die vergleichend-reizphysiologische Felderung der Großhirnrinde unter besonderer Berücksichtigung der menschlichen. Naturwissenschaften. 1926;14:1190–4.

[18] Vogt O. Der Wert der myelogenetischen Felder der Großhirnrinde (Cortex pallii). Anat Anz. 1906;29(11,12):273–87.

[19] Vogt O. Über strukturelle Hirnzentra mit besonderer Berücksichtigung der strukturellen Felder des Cortex pallii. Anat Anz. 1906b;29:74–114.

[20] Braitenberg V. Die Gliederung der Stirnhirnrinde auf Grund ihres Markfaserbaus (Myeloarchitektonik). In: Rehwald E, editor. Das Hirntrauma. Stuttgart: Thieme; 1956. p. 183–203.

[21] Nieuwenhuys R, Broere CA, Cerliani L. A new myeloarchitectonic map of the human neocortex based on data from the Vogt-Vogt school. Brain Struct Funct. 2015;220(5):2551–73. 10.1007/s00429-014-0806-9 Erratum in: Brain Struct Funct. 2015;220(6):3753–5. PMID: 24924165. 24924165

[22] Nieuwenhuys R, Broere CA. A map of the human neocortex showing the estimated overall myelin content of the individual architectonic areas based on the studies of Adolf Hopf. Brain Struct Funct. 2017;222(1):465–80. 10.1007/s00429-016-1228-7, E pub 2016 May 2. PMID: 27138385. PMC5225164 27138385

[23] Nieuwenhuys R, Broere CAJ. A detailed comparison of the cytoarchitectonic and myeloarchitectonic maps of the human neocortex produced by the Vogt-Vogt school. Brain Struct Funct. 2020;225(9):2717–33. 10.1007/s00429-020-02150-2. 33141295

[24] Braitenberg V. A note on myeloarchitectonics. J Comp Neurol. 1962;118:141–56. 10.1002/cne.901180202. PMID: 13872421. 13872421

[25] Hopf A. Über eine Methode zur objektiven Registrierung der Myeloarchitektonik der Hirnrinde [On a method for the objective registration of the myeloarchitectonics of the cerebral cortex]. J Hirnforsch. 1966;8(4):301–13. PMID: 4861030. 4861030

[26] Hopf A. Photometric studies on the myeloarchitecture of the human temporal lobe. J Hirnforsch. 1968;10(4):285–97. 5732948

[27] Hopf A. Registration of the myeloarchitecture of the human frontal lobe with an extinction method. J Hirnforsch. 1968;10:259–69. 4895364

[28] Hopf A. Photometric studies on the myeloarchitecture of the human parietal lobe. I Parietal region. J Hirnforsch. 1969;11:253–65. 5383239

[29] Hopf A. Photometric studies on the myeloarchitecture of the human parietal lobe. II Postcentral region. J Hirnforsch. 1970;12:135–41. 5499693

[30] Kinney HC, Brody BA, Kloman AS, Gilles FH. Sequence of central nervous system myelination in human infancy. II. Patterns of myelination in autopsied infants. J Neuropathol Exp Neurol. 1988;47:217–34. 10.1097/00005072-198805000-00003. 3367155

[31] Peters G. Neuropathologie und Psychiatrie. Psychiatrie der Gegenwart. Vol. I/1A. Grundlagenforschung zur Psychiatrie Teil A. Berlin-Heidelberg-New York: Springer; 1967. p. 286–324.

[32] Lashley KS, Clark G. The cytoarchitecture of the cerebral cortex of Ateles; a critical examination of architectonic studies. J Comp Neurol. 1946;85(2):223–305. 10.1002/cne.900850207 PMID: 21002789. 21002789

[33] Bailey P, von Bonin G. The isocortex of man. Urbana: Univ. of Illinois Press; 1951.

[34] Hopf A. Zur architektonischen Gliederung der menschlichen Hirnrinde. Kritische Bemerkungen zu modernen Strömungen in der Architektonik. J Hirnforsch. 1954b;1:442–95.

[35] Hopf A. Zur Frage der Konstanz und Abgrenzbarkeit myeloarchitektonischer Rindenfelder. [Uniformity and definability of myeloarchitectonic cortical areas]. Dtsch Z Nervenheilkd.1954:172(2);188–200. PMID: 13220256. 10.1007/BF00244203. 13220256

[36] Annese J, Pitiot A, Dinov ID, Toga AW. A myelo-architectonic method for the structural classification of cortical areas. Neuroimage. 2004;21(1):15–26. 10.1016/j.neuroimage.2003.08.024. PMID: 14741638. 14741638

[37] Piredda GF, Hilbert T, Thiran JP, Kober T. Probing myelin content of the human brain with MRI: A review. Magn Reson Med. 2021;85(2):627–52. 10.1002/mrm.28509. Epub 2020 Sep 16. PMID: 32936494. 32936494

[38] Geyer S, Weiss M, Reimann K, Lohmann G, Turner R. Microstructural parcellation of the human cerebral cortex – from Brodmann’s post-mortem map to in vivo mapping with high-field magnetic resonance imaging. Front Hum Neurosci. 2011;5:19. 10.3389/fnhum.2011.00019. PMC3044325 21373360

[39] Glasser MF, Van Essen DC. Mapping human cortical areas in vivo based on myelin content as revealed by T1- and T2-weighted MRI. J Neurosci. 2011;31(32):11597–616. 10.1523/JNEUROSCI.2180-11.2011. PMID: 21832190; PMCID: PMC3167149. PMC3167149 21832190

[40] Lungwitz W. Zur myeloarchitektonischen Untergliederung der menschlichen Area praeoccipitalis (Area 19 Brodmann). J Psychol Neurol (Lpz). 1937;47:607–38.

[41] Strasburger EH. Die myeloarchitektonische Gliederung des Stirnhirns beim Menschen und Schimpansen. I. Teil. Myeloarchitektonische Gliederung des menschlichen Stirnhirns. J Psychol Neurol (Lpz). 1937a;47(5):461–91.

[42] Strasburger EH. Vergleichende myeloarchitektonische Studien an der erweiterten Brocaschen Region des Menschen. J Psychol Neurol (Lpz). 1938;48(5, 6):477–511.

[43] Gerhardt E. Der Isocortex parietalis beim Menschen. J Psychol Neurol. 1940;49:367–419.

[44] Sanides F. Die Architektonik des menschlichen Stirnhirns: zugleich eine Darstellung der Prinzipien seiner Gestaltung als Spiegel der stammesgeschichtlichen Differenzierung der Großhirnrinde [Architectonics of the human frontal lobe of the brain. Monogr Gesamtgeb Neurol Psychiatr. Hrsg. M. Müller-Bern, H. Spatz, Frankfurt, P. Vogel]. Berlin-Göttingen-Heidelberg: Springer; 1962. PMID: 13976313. http://www.thehumanbrain.info/brain/db_monographs/sanides_1962_txt_hires.pdf.

[45] Sanides F. The cyto-myeloarchitecture of the human frontal lobe and its relation to phylogenetic differentiation of the cerebral cortex. J Hirnforsch. 1964;6(5):269–82. PMID: 14227452. 14227452

[46] Hopf A. Die Myeloarchitektonik des Isocortex temporalis beim Menschen. J Hirnforsch. 1954;1:208–79.

[47] Hopf A. Über die Verteilung myeloarchitektonischer Merkmale in der isokortikalen Schläfenlappenrinde beim Menschen. J Hirnforsch. 1955;2:36–54.

[48] Henneberg R. Messung der Oberflächenausdehnung der Großhirnrinde. J Psychol Neurol (Lpz). 1910;17:144–58.

[49] Mai JK, Majtanik M, Paxinos G. Atlas of the Human Brain. San Diego: Academic Press/Elsevier; 2016.

[50] Mai JK, Majtanik M. Architectonic and functional cortical areas registered to the “Atlas of the Human Brain” in MNI/ICBM space. Wahington, TT57 187.08: Soc Neusrosc; 2014.

[51] Mai JK. Brain Collection of the C. and O. Vogt Institute Duesseldorf. 3 Vols. (unpublished); 1985.

[52] Ngowyang G. Die Zytoarchitektonik der Felder des Gyrus rectus. J Psychol Neurol (Lpz). 1932;44:475–84.

[53] Sanides F. Vorläufige Darstellung eines histologischen Phänomens an cytoarchitektonischen Feldergrenzen. J Hirnforsch. 1958;4(4):273–313. 13673160

[54] Hopf A. Über die Verteilung myeloarchitektonischer Merkmale in der Stirnhirnrinde beim Menschen. J Hirnforsch. 1956;2:311–33. https://www.thehumanbrain.info/database/db_literature_structures/HopfAdolf3.pdf. 13376888

[55] Hopf A, Vitzthum H. Über die Verteilung myeloarchitektonischer Merkmale in der Scheitellappenrinde beim Menschen. J Hirnforsch. 1957;3:79–104. 10.1515/9783112522387/html. https://thehumanbrain.info/brain/db_literature/HopfAdolf2.pdf. 13475763

[56] Beck E. Die myeloarchitektonische Felderung des in der Sylvischen Furche gelegenen Teiles des menschlichen Schläfenlappens. J Psychol Neurol (Lpz). 1928;36:1–12.

[57] Vitzthum H, Sanides F. Entwicklungsprinzipien der menschlichen Sehrinde. In: Hassler H, Stephan H, editors. Evolution of the forebrain. Stuttgart: Thieme Verlag; 1966.

[58] Van Essen DC. Cortical cartography and Caret software. NeuroImage. 2012;62:757–64. 10.1016/j.neuroimage.2011.10.077 PMC3288593 22062192

[59] Mai JK, Majtanik M. Mapping of Cortex Areas from historic and recent Studies to the “Atlas of the Human Brain” in standard (MNI-) space for direct comparison in a novel multidimensional data representation. Washington, 2016-S-6296-SfN: Soc Neurosci; 2016.

[60] Ngowyang G. Die Cytoarchitektonik des menschlichen Stirnhirns, I. Teil. Cytoarchitektonische Felderung der Regio granularis und Regio dysgranularis. Natl Res Inst Psychol Acad Sin. 1934;934(7):1–68.

[61] Wang H, Yushkevich PA. Multi-atlas segmentation with joint label fusion and corrective learning-an open source implementation. Front Neuroinformatics. 2013;7:27. 10.3389/fninf.2013.00027 PMC3837555 24319427

[62] Vogt O. Die Myeloarchitektonik des Isocortex parietalis. J Psychol Neurol (Lpz). 1911;18:379–90.

[63] von Economo CF, Koskinas GN. Die Cytoarchitektonik der Hirnrinde des erwachsenen Menschen. Berlin: Springer; 1925.

[64] Vogt BA. in Mai JK, Majtanik M, Paxinos G. Atlas of the Human Brain. San Diego: Academic Press/Elsevier; 2016.

[65] Sarkisov SA, Filimonoff IN, Kononowa EP, Preobraschenskaja IS, Kukuew LA. Atlas of the cytoarchitectonics of the human cerebral cortex. Moscow: Medgiz [Atlas citoarhitektoniki: kory bolʹšogo mozga čeloveka. Akademija Medicinskih Nauk SSSR (Moskva)]; 1955.

[66] Yeo BT, Krienen FM, Sepulcre J, Sabuncu MR, Lashkari D, Hollinshead M, et al. The organization of the human cerebral cortex estimated by intrinsic functional connectivity. J Neurophysiol. 2011;106(3):1125–65. 10.1152/jn.00338.2011. Epub 2011 Jun 8. PMID: 21653723. PMCID: PMC3174820. PMC3174820 21653723

[67] Yarkoni T, Poldrack RA, Nichols TE, Van Essen DC, Wager TD. Large-scale automated synthesis of human functional neuroimaging data. Nat Methods. 2011;268(8):665–70. 10.1038/nmeth.1635. PMID: 21706013. PMCID: PMC3146590. PMC3146590 21706013

[68] Nitschke JB, Sarinopoulos I, Mackiewicz KL, Schaefer HS, Davidson RJ. Functional neuroanatomy of aversion and its anticipation. Neuroimage. 2006;29(1):106–16. 10.1016/j.neuroimage.2005.06.068. Epub 2005 Sep 21. PMID: 16181793. 16181793

[69] Maltby N, Tolin DF, Worhunsky P, O’Keefe TM, Kiehl KA. Dysfunctional action monitoring hyperactivates frontal-striatal circuits in obsessive-compulsive disorder: an event-related fMRI study. Neuroimage. 2004;1524(2):495–503. 10.1016/j.neuroimage.2004.08.041, PMID: 15627591. 15627591

[70] Leppä M, Korvenoja A, Carlson S, Timonen P, Martinkauppi S, Ahonen J, et al. Acute opioid effects on human brain as revealed by functional magnetic resonance imaging. Neuroimage. 2006 Jun;31(2):661–9. 10.1016/j.neuroimage.2005;12.019. PMID: 16459107. 16459107

[71] Vogt BA, editor. Cingulate Neurobiology and Disease. New York: Oxford University Press Inc.; 2009.

[72] Mars RB, Coles MG, Grol MJ, Holroyd CB, Nieuwenhuis S, Hulstijn W, et al. Neural dynamics of error processing in medial frontal cortex. Neuroimage. 2005;28(4):1007–13. 10.1016/j.neuroimage.2005.06.041. Epub 2005 Aug 1. PMID: 16055352. 16055352

[73] Amodio DM, Frith CD. Meeting of minds: the medial frontal cortex and social cognition. Nat Rev Neurosci. 2006;7(4):268–77. 10.1038/nrn1884. PMID: 16552413. 16552413

[74] Bermpohl F, Pascual-Leone A, Amedi A, Merabet LB, Fregni F, Gaab N, et al. Dissociable networks for the expectancy and perception of emotional stimuli in the human brain. Neuroimage. 2006;30(2):588–600. 10.1016/j.neuroimage.2005.09.040. Epub 2005 Nov 7. PMID: 16275018. 16275018

[75] Jackson PL, Meltzoff AN, Decety J. How do we perceive the pain of others? A window into the neural processes involved in empathy. Neuroimage. 2005;24(3):771–9. 10.1016/j.neuroimage.2004.09.006. PMID: 15652312. 15652312

[76] Zilles K, Palomero-Gallagher N, Amunts K. Myeloarchitecture and maps of the cerebral cortex. In: Toga AW, editor. Brain mapping: an encyclopedic reference. San Diego: Elsevier Academic.; 2015. p. 137–56.

[77] Glasser MF, Goyal MS, Preuss TM, Raichle ME, Van Essen DC. Trends and properties of human cerebral cortex: Correlations with cortical myelin content. Neuroimage. 2014;93:165–75. 10.1016/j.neuroimage.2013.03.060 PMC3795824 23567887

[78] Sanides F. Grenzerscheinungen an myeloarchitektonischen Feldergrenzen. Zugleich ein Beitrag zur Morphokinese der menschlichen Grosshirnrinde [Margin phenomena on the myeloarchitectonic field margins. With a contribution to the morphokinesis of the cerebral cortex]. Dtsch Z Nervenheilkd. 1960;180:381–405, PMID: 14441512. 14441512

[79] Mai JK, Majtanik M. MRX Atlas of the Human Brain (unpublished), representing the left hemisphere of the “Atlas of the Human Brain. San Diego additionally integrating information from histological and histochemical sections of other brains from the Dept. of Anatomy I, Duesseldorf: Academic Press; 2010.

[80] Beck E. A cytoarchitectural investigation into the boundaries of cortical areas 13 and 14 in the human brain. J Anat. 1949;83(2):147–57. PMID: 17105075; PMCID: PMC1273153 . https://pubmed.ncbi.nlm.nih.gov/17105075/. PMC1273153 17105075

[81] Kreht H. Cytoarchitektonik und motorisches Sprachzentrum. Z mikr-anat Forsch (Lpz). 1936;39:331–31.

[82] Kreht H. Zur Volumengröße der architektonischen Felder 55-56 einiger menschlicher Gehirne im Vergleich zu der des Schimpansen und Orang-Utan. Z mikrosk-anat Forschg (Lpz). 1936b;39:409–14.

[83] Busch K-T. Individuelle architektonische Differenzen der Area striata. J Hirnforsch. 1960;4(6):535–52.

[84] Foit NA, Yung S, Lee HM, Bernasconi A, Bernasconi N, Hong SJ. A whole-brain 3D myeloarchitectonic atlas: Mapping the Vogt-Vogt legacy to the cortical surface. Neuroimage. 2022;263:119617. 10.1016/j.neuroimage.2022.119617. PMID: 36084859. 36084859

[85] Zachlod D, Kedo O, Amunts K. Anatomy of the temporal lobe: From macro to micro. Handb Clin Neurol. 2022;187:17–51. 10.1016/B978-0-12-823493-8.00009-2. PMID: 35964970. 35964970

[86] Sewards TV. Adolf Hopf’s 1954 myeloarchitectonic parcellation of the human temporal lobe: a review and assessment. Brain Res Bull. 2011;86(5–6):298–313. 10.1016/j.brainresbull.2011.08.010. PMID: 21888952. 21888952

[87] Catani M, Thiebaut de Schotten M. A diffusion tensor imaging tractography atlas for virtual in vivo dissections. Cortex. 2008;44(8):1105–32. 10.1016/j.cortex.2008.05.004, E pub 2008 May 23. PMID: 18619589. 18619589

[88] Glasser MF, Coalson TS, Robinson EC, Hacker CD, Harwell J, Yacoub E, et al. A multi-modal parcellation of human cerebral cortex. Nature2016 536((7615):171–8. 10.1038/nature18933 Epub 2016 Jul 20. PMID: 27437579; PMCID: PMC4990127. PMC4990127 27437579

[89] Vogt O, Vogt C. Sitz und Wesen der Krankheiten im Lichte der topistischen Hirnforschung und des Variierens der Tiere. Erster Teil: Befunde der topistischen Hirnforschung als Beitrag zur Lehre vom Krankheitssitz. Leipzig Barth; 1937.

[90] Llull R. Raimundi Lulli Ars generalis ultima. Lyon-Pisa. p. 1305–8. https://monoskop.org/Ramon_Llull.

[91] Knauer A. Die Myeloarchitektonik der Brocaschen Region. Neurol Centralbl. 1909;28:1240–3.

[92] Riegele L. Die Cytoarchitektonik der Felder der Broca’schen Region. J Psychol Neurol (Lpz). 1931;42:496–515.

[93] Strasburger EH. Die myeloarchitektonische Gliederung des Stirnhirns beim Menschen und Schimpansen. II. Teil. Der Faserbau des Stirnhirns beim Schimpansen. J Psychol Neurol (Lpz). 1937b;47(6):565–606.

[94] Batsch E-G. Die myeloarchitektonische Untergliederung des Isocortex parietalis beim Menschen. [Myeloarchitectonic sub-classification of isocortex parietalis in man]. J Hirnforsch. 1956;2(2/3):225–58. PMID: 13376885. 13376885

[95] Schulze HA. Zur individuellen cytoarchitektonischen Gestaltung der linken und rechten Hemisphäre des Lobulus parietalis inferior. J Hirnforsch. 1960;4(6):486–534.

[96] Beck E. Zur Exaktheit der myeloarchitektonischen Felderung des Cortex cerebri. J Psychol Neurol. 1925;31:281–8.

[97] Beck E. Die Myeloarchitektonik der dorsalen Schläfenlappenrinde beim Menschen. J Psychol Neurol (Lpz). 1930;41:129–262.

[98] Sgοninα K. Zur vergleichenden Anatomie der Entorhinal- und Präsubikularregion. J Psych Neurol (Lpz). 1937(1–2):56–163.

[99] Vogt M. Über fokale Besonderheiten der Area occipitalis im cytoarchitektonischen Bilde. J Psychol Neurol (Lpz). 1929;39:506–10.

[100] Heinze G. Die Regio occipitalis bei “Ausnahmegehirnen” dargestellt an 12 Hemisphären. Dissertation Freiburg/Brsg; 1952.

[101] Heinze G. Zytoarchitektonische Untergliederung der Area occipitalis. J Hirnforsch. 1954;1:173–98.

[102] Brockhaus H. Die Cyto- und Myeloarchitektonik des Cortex claustralis und des Claustrum beim Menschen. J Psychol Neurol (Lpz). 1940;49(4–6):249–348.

[103] Rose M. Die Inselrinde des Menschen und der Tiere. J Psychol Neurol (Lpz). 1928;37:467–624.

[104] Rose M. Cytoarchitektonik und Myeloarchitektonik der Großhirnrinde. In: Bumke Ou, Foerster O, editors. Handbuch der Neurologie, Allgemeine Neurologie 1, Anatomie. 1, Berlin: Springer; 1935. p. 588–778. http://www.thehumanbrain.info/brain/db_monographs/rose_m_1935_grosshirnrinde.pdf.

[105] FIPAT Federative International Programme for Anatomical Terminology. 2017. https://ifaa.net/committees/anatomical-terminology-fipat/

[106] Wallace DL. A method for comparing two hierarchical clusterings: comment. J Am Stat Assoc. 1983;78:569–76.

[107] Pinto FR, Melo-Cristino J, Ramirez M. A confidence interval for the Wallace coefficient of concordance and its application to microbial typing methods. PLoS One. 2008;3(11):e3696. 10.1371/journal.pone.0003696. PMC2577298 19002246

[108] Hömke L. A multigrid method for elastic image registration with additional structural constraints. PhD thesis. Düsseldorf, Germany: Heinrich-Heine University; 2006.

[109] Löchel D. Morphological multi-modal image registration (Morphologische multimodale Bildanpassung). Diploma-Thesis. Düsseldorf, Germany: Heinrich-Heine University; 2007.

[110] Chen H, Varshney PK. A pyramid approach for multimodality image registration based on mutual information. Proceedings of the 3rd International Conference on Information Fusion, FUSION 2000. 1. MOD3/9 - MOD315 Vol. 1; 2000. 10.1109/IFIC.2000.862613.

[111] Beg MF, Miller MI, Trouvé A, Younes L. Computing large deformation metric mappings via geodesic flows of diffeomorphisms. Int J Computer Vis. 2005;61(2):139–57.

[112] Tustison NJ, Avants BB, Cook PA, Zheng Y, Egan A, Yushkevich PA, et al. N4ITK: improved N3 bias correction. IEEE Trans Med imaging. 2010;29(6):1310–20. 10.1109/TMI.2010.2046908 PMC3071855 20378467

[113] Shinohara RT, Sweeney EM, Goldsmith J, Shiee N, Mateen FJ, Australian Imaging Biomarkers Lifestyle Flagship Study of Ageing, & Alzheimer’s Disease Neuroimaging Initiative, et al. Statistical normalization techniques for magnetic resonance imaging. NeuroImage Clin. 2014;6:9–19. 10.1016/j.nicl.2014.08.008.

