# Supplementary material for: Myeloarchitectonic maps of the human cerebral cortex registered to surface and sections of a standard atlas brain
Source: Transl Neurosci. 2023 Dec 26;14(1):20220325. doi: 10.1515/tnsci-2022-0325 (PMC10751573; doi:10.1515/tnsci-2022-0325)
Supplement: Supplementary material [file tnsci-2022-0325-sm.pdf]

Supplementary material

SUPPLEMENT 1: Isocortex Frontalis

The first column shows the numbers of the myeloarchitectonic fields for the frontal isocortex (Fields 1–66). We distinguish between myeloarchitectonically characterized “Fields” (F) and cytoarchitectonically characterized “Areas” (A). The second and the third columns separate Subregions (SR) from Divisions (Divisio, Div). The fourth column provides the name for each distinguished myeloarchitectonic field. The right sided column indicates where representations of the myeloarchitectonic cortical structure (drawings or photographs, Ph) can be found that illustrate this field (Br: Braitenberg [20]; H66: Hopf [25]; H68: Hopf [27] (ext = extinction method); R: Rose [113]; S: Sanides [44]; S1: Strasburger [41]; S2: Strasburger [42]; S2F: Strasburger [42]:photographs; V: Vogt [15]; VV: Vogt and Vogt [16]). The last column provides very rough information about the typical and differentiating characteristics of each individual field. An attempt has been made to reproduce the original description as accurately as possible. A list of abbreviations and references not listed in the reference list can be found at the end of this table. References can be found in the main text.

Isocortex frontalis

1. R. UNISTRIATA EURADIATA TENUIFIBROSA		**Figures**	**Comments**
Areae 1 – 14 *(≙ F1 recta)*			
**Area**	***Subregio eutangentialis***		
	Div. trizonalis	L-1° is marked by a very narrow width.	
**1**	A. pauper	VV	Unistriate with slightly paler sublayer L-6aα, many many fine basic fibers and only slightly thicker individual fibers. Strasburger [41: A39 R] bizonal (to light subquadrizonal). F1 has significantly fewer fibers than F2.<br>**unistriate**, belongs to the **poor fiber group, slightly euradiate, L-4 = 5b**
**2**	A. dives	S1, VV	Area dives is well defined, located where the fields F1d, F1 recta, F2d, F2v abut (Braitenberg [20]. Gennari (B1) type (only the upper layer is visible; very fibrous). L-1° narrow; L-1a+b with numerous Ef: therefore slightly trizonal. L-4-6 is considerably richer in Ef and Gf than F50 (5b is relatively prominent in the caudal part). L-6aαless prominent. The individual Gf (basic fibers) and Ef in all layers are of a somewhat coarse type.<br>**Unistriate**, belongs to the **fiber rich group, euradiate**
	Div. subquadrizonalis	The L-1° is thicker, and the L-1a is always clearly distinguished from L-1b due to its paucity in Gf and especially Ef.	
**3**	A. propetrizonalis		L-1a is still relatively rich in fibres. All layers contain fewer Gf than F2 or F1. Ef in layers 4-6 are thinner as compared with F2, but thicker compared with F1.<br>F3 cannot be distinguished from 4 with certainty.<br>**unistriate, poor fiber category, slightly euradiate, L-4 = 5b**
**4**	A. subquadrizonalis pauper	VV	This field shows L-1 as bright subquadrizonal; L-2-6 show more Gf and in L-3-6 thinner Ef than in F3. It differs from the F3 because it has fewer fibers in L-1a, thicker Ef in the L-1b, and thinner ones in L-3-6. On the other hand, it has more basic Gf in layers 2-6bα and thinner Ef in layers 3-6. The radii are more developed than in F-10 and in F-11. In comparison with F1, F4 contains significantly less fibers in layers 1b-6bα than F-50 and F-33 but these fibers are coarser.<br>**unistriate, poor fiber category, slightly euradiate, L-4 = 5b**

(Continued)

**5**	A. subquadrizonalis dives	H68 (ext)	F5 is characterized by a greater abundance of fibers in all layers compared with F4. L-1 lucid, **bi-** to **subquadrizonal** (fewer fibers than in F8, F9); Gf more abundant in L-3-6 than in L-1 and 4. F5 is similar to F52; contains generally less fibers than F52.<br>**unistriate, poor fiber category, slightly euradiate, L-4 = 5b**
	Div.quadrizonalis		Sublayer 1° is even thicker, and the L-1b contains thicker Ef than the *Divisio subquadrizonalis*
**6**	A. propeultratangentialis	S2, VV	Difficult to separate from F4. F 6 contains more fibres in all layers and more developed radii if compared with F4.<br>F 6 contains numerous thick Ef in the L-1c and therefore resembles the *ultratangential* subregion. In comparison with F4, F6 contains less fibres in layers 2-6.<br>In L-1c numerous thick individual fibers appear, resulting in an approximation of the *Subregio ultratangentialis*. S2: euradiate.<br>**unistriate, poor fiber category, medioradiate, L-4 = 5b;**
**7**	A. quadrizonalis pauper.	VV	In L-1 fewer and thinner Gf than are present than in the adjacent areas F4 and F6. In L-2-6 there are also fewer and thinner fibers. In comparison with F4 and F6 this field (7) has a smaller number of thin Gf in the L-1 as well as smaller numbers of thin Ef in layers 2-6.<br>Strasburger [93] could not prove this field, like F29, with certainty)
**8**	A. quadrizonalis intermedia		F8 contains more fibers than F7. L-1 is clearly more fibrous than in F4 and F11, but significantly less fibrous than in F62 and F66. S2: mediodiate.<br>**unistriate, poor fiber category, medio- to euradiate, L-4 = 5b**
**9**	A. quadrizonalis dives	S2, VV	F9 is located at the transition to the *Regio unitostriata*, shows a greater abundance of fibers than the other areas of the *Divisio quadrizonalis*, whereby it represents a transition towards the unitostriate region (especially towards its F62).<br>F9 is subquadrizonal; similar to F8 but with coarser Ef in the sublayer 1b and in L6. Generally less fibrous than F62.<br>**unistriate, fibrous part, slightly euradiate, 4 = 5b**
**10-14**	***Subregio ultratangentialis***		
	Div.euradiata		
**10**	A. subultratangentialis	H68-ext; S2; VV	F10 is quadrizonal, euradiary with isolated ultratangential fibers (like F10,11,12) in L2. Its Ef in layers 3-6 are thinner compared to the F3, but coarser than in F4 and F6.<br>**unistriate, poor fiber category, medioradiate, L-4 = 5b**
**11**	A. ultratangentialis quadrizonalis		L-1 bright bi- to quadrizonal; a greater amount of coarse *ultratangential* fibers is found in layers 2+3a compared with F10 and F6. The layers 3-6, particularly L-6, contain a lesser amount of fibres. In L-4 they are coarser than in F21 and F12.<br>Compared to F13 L-1 has fewer but L-4 shows more fibers.<br>**unistriate, poor fiber category, medioradiate, L-4 = 5b**
**12**	A. ultratangentialis propetrizonalis	VV	Ef are missing in L-1a, but plenty in L-1b; L-1c contains relatively many Gf. L-2 shows numerous thick individual fibers. The other layers are characterized by a very low fiber content, but L-4 stands out quite clearly from L-3 and 5a.<br>F12 compares to F21 but shows more ultratangential fibers in L-1c and 2.<br>**unistriate, poor fiber category, infraradiate, L-4 = less intense than L-5b**

(Continued)

	Div.propesupraradiata	Trizonal, with very few fibers in the 3rd to 6th layer, but more radial fibers in L-2 and L-3a than in F12.	
**13**	A. propesupraradiata	S1	Trizonal. L3-6 contain very few (E-, G-) fibres, but increased numbers of radial fibres in L-3a and in L-2.<br>Compared with F15 L-1a+b has somewhat fewer fibers, L-1c more (ultratangential) fibers. Radii are as poorly developed as in F15.<br>**unistriate, poor fiber category, infraradiate, L-4 = less intense than 5b**
	Div.supraradiata	This *Divisio* has very many horizontal Gf in L-1c and L-2, very thick Ef in L-1c-5a. L-3b-6aα are rudimentary so that L-5a does not stand out at all; in L-3a thin radial fibers are seen.	
**14**	A. supraradiata	S2,<br>14b: VV	F14 is not uniform. Strasburger [41] regards this area a part of the allocortex.<br>L-1c contains numerous Gf; the layers 1-5a (caudally also sublayer 1a+b) exhibit many very thick (ultratangential) Ef. Layers 3b to 6aα are rudimentary, making it difficult to delineate L-5a. This area contains even more radial fibers in L-2 and even thinner radial bundles in L-3a.<br>At the bottom of the sulcus corporis callosi, the Area 14 is present in its rudimentary form and continues around the genu corporis callosi in the caudal direction. At the caudal border of the *Pars frontalis* it undergoes a characteristic architectonic transformation - the lateral Lancisi's stria appears here.
2. R. UNISTRIATA EURADIATA INFRARADIATA<br>Areae 15- 32		This region coincides with the cytoarchitectural region of the same name.	
**15-16**	***Subregio extrema***	Ultratangential fibres in L-1c and in the normally fiber poor L-2 (Lamina dysfibrosa) extend to L-4; layers 3a and 3b are very narrow; layers 4+5a cannot be separated. Layers 5a+4 contain only rudimentary radii.	
**15**	A. pauper		F15 is exceptionally quadrizonal (Rose [113]; Strasburger [41]: trizonal) with L-1a containing fewer horizontal fibers than F12-14; L-5 is almost missing. In comparison to F12-14 it contains less horizontal fibres in sublayer 1a, but more of these fibres in L-4-5b; compared to F17 and F21 it shows more numerous fibers.<br>**unistriate, infraradiate** (L-4= less intense than L-5b)
**16**	A. dives	S2	F16 contains numerous Ef in L-1a, and therefore assumes trizonal or at least the propetrizonal appearance. L-5b-6 contain more and coarser fibres than F-15 but has significantly less fibers than F-14<br>**unistriate, infraradiate** (L-4= less intense than 5b)
**17-24**	***Subregio typica***	Characteristic is the appearance of very strong individual fibers in L-1c, which also occur in the normally very fiber-poor L-2 (lamina dysfibrosa). These so-called ultratangential fibers are typical in parts of the allocortex and at their transitions to the socortex [114, 45].	
	Div.infraradiata	The division shows only very rudimentary radii in L-5a+4 and is very poor in fibers in the outer layers, especially in L-3b and 4.	
	Subdivisio quadrizonalis		Both fields are characterized by hardly recognizable individual fibers in L-3b-6.
**17**	A. tenuifibrosa	S1, VV	F17 is *trizonal*, in places *subquadrizonal*.<br>In L-1 four layers are distinguished, L-1c, like L-2, is characterized by the presence of individual fibers. L-3 has extraordinarily few fibers. Also, Ef in layers 3b-5a are not be observed and L-4 and L-5a are barely visible due to the lack of horizontal fibers. However, L-5b stands out more due to the occurrence of coarser horizontal fibers. 6aα shows a decrease of fibers, whereas increasing numbers of fibers are present in 6bα. The radii end at the boundary of L-5a and L-5b.<br>No Ef are observed in layers 3b-5a.

(Continued)

			L-1 is more fibrous than in F21 and F25; slightly more fibrous than in F18. L-4 is much less fibrous than in F25. L-4-6 and radii are less developed than in F18 and F25. L-4-6 shows fewer fibers than in F16, the radii are somewhat weaker than in F21. **unistriate, poor fiber category, infraradiate, L-4 = less intense than L-5b**
**18**	A. intermedia	S2	F18 shows more fibers in the sublayer 1a, but less ultratangential fibres; distinct Ef in L-3b-5a and more Gf in L-3b-6. **unistriate, poor fiber category, infraradiate, L-4= less intense than L-5b**
	Subdivisio propetrizonalis		
**19**	A. grossofibrosa	S1, VV	F19 is slightly trizonal. It contains numerous coarse Gf in layers 1a, 1b and 3-6; coarser Ef in layers 1a, 1b and 3b-6, but almost no ultratangential fibres. The radii are considerably thicker than in F-17, but weaker than in the dorsal neighboring area and than in F25. L-4-6 (especially L-4) contain fewer fibers than the dorsal neighboring area and than in F25, but exhibit more coarse fibers that F17. **unistriate, poor fiber category, infraradiate, L-4 = less intense than L-5b**
**20**	A. reuniens		F20 contains fewer thick Ef in L-5a whereas the radii in this layer are somewhat better developed; sublayer 6aα is clearly paler. This area represents a transition to the adjacent (caudal) area of the *Pars parietalis*. F20 is very similar to F24, but L-4 is significantly richer in fibers. It forms a transitional field to the parietal area **unistriate, poor fiber category, infra- to medioadiate, F-4 = less intense than F-5b**
	Div. propemedioradiata	Layers 3b+4 contain a slightly larger amount of fibers and layers 5a+4 contain a larger amount of radial fiber bundles.	
	Subdivisio quadrizonalis		
**21**	A. tenuifibrosa	S1, S2	L-1 is very bright and *quadrizonal*. In the posterior region isolated *ultratangential* fibers stand out in L-1c and L-2. L-4 is significantly less intense than in F25, but somewhat stronger than in F13, 15, 17 (F15 and F17 are trizonal); L-4 contains more fibers than F13. **unistriate, poor fiber category, infraradiate, L-4 = less intense than L-5b**
	Subdivisio propetrizonalis		
**22**	A. intermedia		Bi- to slightly quadrizonal. L-4 is barely visible. In L-4-6 the radii are considerably weaker than in F26. F22 shows a structure that is similar to F18, but L-4 and L-5a are richer in fibers. Radii in L-4-6 are weaker than in F25. **unistriate, poor fiber category, infraradiate, L-4 = less intense than L-5b**
**23**	A. grossofibrosa	H68:ext, S2, VV	Trizonal; appears like F19 and F22. In L-4-6 the radii are much weaker than in F27. **unistriate, poor fiber category, infraradiate, 4 L-4 = less intense than L-5b**
**24**	A. reuniens		Bizonal; F24 appears similar to F20. L-1 shows less fibers than F23. L-4-5b (esp. L-4) contain significantly more fibers than F23. Medioradiate (radii stronger than in F23). In L-4-6 radii are, however, significantly weaker than in F27 and F28. F24 represents a transition field to the parietal area, and therefore falls somewhat out of the structural plan of this group. **unistriate, poor fiber category, infraradiate, L-4 = less intense than L-5b**

(Continued)

**25-32**	***Subregio medioradiata***		This subregion occupies the most dorsal part of the *Gyrus limbicus*. One finds an increase in the horizontal fibers in the 3rd and 4th layers, a clear separation of layers 4 and 5a, and more radii in L-4 and L-5a.
	Div.medioradiata		
	Subdivisio quadrizonalis (γαα of Vogt, 1910)		Compared to the *Subdivisio subtrizonalis*, this subdivision has fewer Gf throughout (with possibly some increase in sublayers 3a and 1a.
**25**	A. pauper	S1	Quadrizonal (F17 is trizonal); L-2 still displays few ultratangential fibres. L-4 (which in F17, 21, 18 is hardly visible) clearly stands out; in L-4-6 marked, rather coarse horizontal fibers. Radii are less developed than in F11 (L-3 and 4); radii and horizontal fibers are obvious in L-4-6 than in F30, 26, 33, 50. L25 probably also includes Vogt's F29. **unistriate, poor fiber category, medioradiate (like F-27,28), 4= 5b**
**26**	A. intermedia	S1	More Gf, thick Ef in sublayers 3b to 6bα, no more ultratangential fibres in the L-2. **unistriate, poor fiber category, medioradiate, L-4 = L-5b**
	Subdivisio subtrizonalis		L-1 is bright quadrizonal. Especially L-3a, but also L-1a. contain a significantly high amount of Gf; thick individual fibers in L-3b-6bα. L-4-6, radii are less developed than in F30, but better than in F-25 and F-22.
**27**	A. dives	H68-ext, S2	Trizonal; similar to F26. Contains more fibres than F28. Radii and L-4 are less developed than in F31. **unistriate, poor fiber category, medioradiate, L-4 = L-5b**
**28**	A. reuniens	S2	Bizonal, significantly less fibers than in F27. Radii and L-4 are less developed than in F32. **unistriate, poor fiber category, medioradiate, L-4 = L-5b**
	Div.propeeuradiata		Occupies mainly in the ventral lip of the callosomarginal sulcus. About half of the Radii in the layer 4 are equally wide as Radii in the L-5b
	Subdivisio quadrizonalis (γβα of Vogt, 1910)		
**29**	A. pauper		F29 differs from F25 by its thick Ef in L-3b-6bα and its denser feltwork in L-4.
**30**	A. intermedia	S1	L-1 quadrizonal. L-3-6 have fewer fibers in F25 and L-26 but more and coarser Gf than the F29. The radii are significantly thinner than in F33-36, but better developed than in F25 and F26.(Strasburger [41] distinguishes between F-29a and b). **unistriate poor fiber category, slightly euradiate, L-4 = L-5b**
	Subdivisio trizonalis		
**31**	A. dives	H68-ext, S2	Ttrizonal, displays the same general characteristics as F27 and F30 (a). **unistriate poor fiber category, slightly euradiate, L-4 = L-5b**
**32**	A. reuniens	S2	L-1 appears extrardinary bright, bizonal, otherwise like F28, 30a, 31. Strasburger [41] also includes a caudally adjoining field with fewer fibers and somewhat fainter radii, which in this respect resembles the adjacent parietal field. **Unistriate, poor fiber category, slightly euradiate, L-4 = L-5b**
3. R. UNISTRIATA EURADIATA GROSSOFIBROSA Areae 33-41 *(≙ R F1 dorsalis)*			Distinctive attributes of this region are richness and caliber of fibers; width of cortex [20]. **Trizonal**, except of F33. It shows at most in F40, F41 and F43 the indication of a brightening in L-5a. All subregions contain thick Ef in L-3b-6.

(Continued)

**33-37**	***Subregio unistriata progrediens***		L-5a is well separated from L-4. The number of fibres, caliber and width of L-1c and L-4 as well as the eufasciate character was described by Vogt as progressively advancing.
	Div.pauper		Among the 3 fields of this division, L-1c progressively widens from F33 to F35; in the same order rises the amount and caliber of the Ef.
**33**	A. latofasciculata	S2	This field displays the smallest amount of fibres and the thinnest Ef of the *Divisio pauper*.<br>L-1 is similar to F50; L-4-6 exhibit fewer Ef (and Gf) than in F34. Radii are weaker and sparser than in F34.<br>**Unistriate, fiber -rich category, euradiate, L-4 = L-5**
**34**	A. sublatofasciculata	S1, S2	This field contains a higher number of thicker Ef. L-1 is very bright, bizonal. Radii and L-3-6 appear somewhat less developed than in F35. In the oral part L-5b appears relatively distinct from 6α.<br>The subfield F-34a [41] is very similar to F34 and F35. It stands between the two with regard to the strength of radii and in general fiber content.<br>**Unistriate, fiber -rich category, euradiate, L-4 = L-5**
**35**	A. aequofasciculata		Slightly trizonal to bizonal (L-1 as in F36). The amount and thickness of the fibers increases even more compared to F33, 34. Compared to F36, F35 has weaker and sparser radii and in L-4-6 fewer Ef.<br>**Unistriate, fiber -rich category, euradiate, L-4 = L-5**
	Div.dives		Compared with the previous division the content and thickness of Ef is increasing, especially in L-3b.
**36**	A. subtenuistriata	H66-ext, S2, VV	Aeqofascicular. L-1a+b are poorly delineated from L-1c; the width of L-4 is about two thirds of the width of L-3b.<br>Bizonal to very gradually trizonal L-1a+b is much brighter than in F37, therefore, L-4-6 appear with somewhat fewer and finer fibers, L-4 is smaller than in F37, but otherwise very similar to it.<br>**Unistriate, fiber -rich category, euradiate, L-4 = L-5**
**37**	A. aequostriata		F37 contains somewhat more fibers and thicker Ef, L-4 and L-3b are of equal width and L-1a+b is much better delineated from L-1c as the Ef of 1a+b are much coarser.<br>Trizonal (L-1 like in F38). L-4-6 contain less fibres, L-5a is somewhat more discernable than in F38. Very similar to F38.<br>**Unistriate, fiber -rich category, euradiate, L-4 = L-5**
**38-39**	***Subregio propeastriata***		The subregion exhibits generally more and thicker fibres with F38 showing somewhat fewer and thinner fibers and F39 thicker fibers. Low contrast between L-5a and L4. L-1c is at least twice as wide as L-1a+b.<br>Fields 38 to 43 differ from the adjacent parietal area in that in the latter, L-5b differs very clearly from L-6α in terms of fiber abundance.
**38**	A. subunistriata	S1; S3	Trizonal, L-1a+b richer in Ef than in F41a, L-2 scarcely brighter than L-1c and L-3a. L-4-6 (especially L-5a) significantly richer in Ef than in F-41a. Low contrasts between layers 5a to 6α. (Strasburger [41] refers to the conflict to allocate the ventral half to either F38 or F40.<br>**Unistriate, fiber -rich category, euradiate, L-4 = L-5**
**39**	A. propeastriata	S2	Trizonal, with more and thicker Ef; L-1a+b darker and L-4-6 much richer in fibers than in F38. L-2 and L-5a poorly demarcated (relatively rich in fibers). F39a is a relatively fiber-poor division of F39, but it shows more fibers than F38.<br>A part of this field (39^{I}) exhibits less Gf and thinner Ef .<br>**Unistriate, fiber -rich category, euradiate, L-4 = L-5**

(Continued)

**40-41**	***Subregio unistriata degrediens***		In this subregion the abundance and coarseness of fibers, the eufasciate character and the width of L-4 and L-1c are progressively decreasing.
**40**	A. dives		Strasburger [41] and Hopf [48] considered F40 of Vogt and Sanides [44] as being an inferior part of area F38.
**41**	A. pauper	S1; S2, VV	Trizonal (L-1 as in F41). Strasburger distinguishes F41a from F41b: F41a is positioned between F38 and F41b regarding structure and fiber content. That means: L-3-6 (and especially L-5a) contain more fibers (esp. Ef) than F41b. F41b is trizonal to slightly subquadrizonal; L-5a is poor in fibers, almost without Ef. F41b is neighboring the Insular fields (see [41].<br>**Unistriate, fiber -rich category, euradiate, L-4** = **L-5**
**42-43**	***Subregio astriata***		This subregion is well delimited. It corresponds to the area gigantopyramidalis (F4 of Brodmann). Is very fibrous and absolutely astriate.
**42**	A. typica	H66/68-ext,<br>S2; VV	F42 is astriate* and very rich in fibers which is reflected by the dark appearance. L-1 3 are especially thin, and L-4-6 are especially wide. L-1a b is noticeably narrower than in F39, 38. L-4 and L-5b are almost astriate. L-4-6 has significantly more fibers than F39. Strasburger [41] distinguished F42a as a fronto-parietal transition area. In it, L-5b stands out more clearly from L-5a and 6α than in F42, but less clearly than in the subsequent parietal area. Otherwise it coincides with F42.<br>**Unistriate, fiber -rich category, euradiate, L-4** = **L-5 *** The astriate character distinguishes F42 from the orally located F39 with a clearly visible B1 stripe and from the caudally situated F67 where B2 is well developed (Braitenberg, 1956).
**43**	A. atypica		F-43, hidden in most parts in the central sulcus (and exposed as a narrow strip only) has fewer fibers than F42, resulting in improving recognition of L-5a and L-6aα. The amount of fibers is, however, higher than in the adjacent parietal region.<br>**Unistriate, fiber -rich category, euradiate, L-4** = **L-5**
4. R. PROPEUNISTRIATA Areae 44-50			In this region, L-5b is more fibrous and therefore more distinct from 6aα than in the *Regio unistriata euradiata grossofibrosa*. L-6aα contains more fibers than the sublayer 5a.
**44-48**	***Subregio grossofibrosa***		Differentiation between F45 from F46 is problematic (Strasburger, 1937 and Hopf, 1956).
	Div. eufasciculata		L-3b-6βα contain coarser Ef.
**44**	A. tenuifasciculata	H68-ext	F44 is somewhat less fibrous and contains thinner Ef than in the neighboring F38. The width of L-1a+b is about half of L-1c.<br>L-1-6 show more fibers than in F45.<br>**Proprunistriate, euradiate, L-4** = **L-5b**
**45**	A. aequofasciculata	H68-ext<br>S1; S2	Bi- to slightly trizonal. This field generally exhibits fewer fibers with thinner Ef. The width of L-1a+b equals L-1c and contains a significant lesser amount of fibres, resulting in uncertain distinction. L-5a appears darker than in F47. Strasburger (1937) regarded Vogt's F45 and F46 as one common field.<br>**Proprunistriate, euradiate, L-4** = **L-5b**
**46**	A. sublatofasciculata		L-1la+b are (about 3/5 times) wider than L-1c. Ef are more delicate than in F44 and F45. Braitenberg [20, p.192] classifies F46-55 an **bistriate**.
	Div. dysfasciculata		
**47**	A. dives	S1; S2	Fiber-rich, bizonal. L-1 is rather light; L-3 contains (in contrast to F54b) numerous Ef. L-4-6 are more fibrous than in F35and F34.<br>**Proprunistriate, euradiate, L-4** = **L-5b**

(Continued)

**48**	A. pauper	S1; S2	Bizonal. Ef even more delicate and sparce in L-3b; L-1 with light appearance; L-3-6 somewhat richer in fibers than in F53, fewer fibers (Ef) than in F47. Radii finer, Gf-feltwork denser than in F34.<br>**Proprunistriate, euradiate, L-4 = L-5b**
**49-50**	***Subregio tenuifibrosa***	As fiber-rich field F49 contrasts with the fiber-poor F50.	
**49**	A. dives	H68-ext	F49 exhibits a light L-1 and a dark (bi- to propeunistriste) L-6a. The overall fiber content and -caliber correspond to F34.<br>**Bistriate, euradiate, 4 less intense than 5b**
**50**	A. pauper	S1	F50 shows less fibers; Ef are of finer caliber. L-1 is bright, bizonal; L-4-6 (esp. L-5a) show less Ef.<br>**Unistriate, fiber-rich category, euradiate, L-4 = L-5**
5. R. BISTRIATA Areae 51-56		Three subregions: bistriate, sub(bi)striate, propeunistriate	
**51-53**	***Subregio bistriata***		
	Div. tenuifibrosa		
**51**	A. pauper	H66,68-ext; S1	Quite similar to the adjacent F-1 (there L-4 = L-5b). L-1 is somewhat brighter, radii in L-4-6 are less distinct than in F52 and F53. L-5b is less evident against L-6α than in F53. Generally fewer fibers than in F2.<br>**Bistriate, euradiate, 4 more tenuous than 5b**
**52**	A. dives	S1	Rather rich in fibers with coarser Ef at the anterior part of the middle frontal gyrus. Very similar to F61, but F52 has fewer fibers (especially Ef) in all layers. Slightly subquadrizonal. F52 is similar to F61 but in all layers somewhat more fibers (esp. Ef) are noted.<br>**Bistriate, euradiate, 4=5b**
	Div. grossofibrosa		
**53**	A. grossofibrosa	B; H68-ext; S1	This field is positioned in terms of structure and general fiber content between F59 and F52. Trizonal, L-1a+b is as fiber-rich as in F59. Difference in fiber content between L4 and 5b is greater than in F59. L-4, 5b appear with less fibers, L-5a has a much lower fiber density than in F59 (approximately as in F52)<br>**Bistriate, euradiate, 4=5b**
**54-55**	***Subregio subbistriata***	The two fields are distinguished by their relative fiber density esp. in L-5a where F55 shows more coarse fibers.	
**54**	A. tenuifibrosa	H66-ext	Trizonal. The overall fiber content (especially of L-5a) between F59a and F53 is very low. L-1a+ b shows somewhat fewer fibers than in F53.<br>**Bistriate, euradiate, L-4 less intense than L-5b**
**55**	A. grossofibrosa	H66, H68-ext, S1	Trizonal, L-1a+b ahows almost as few fibers as in F54. In L-1-3 hardly any Ef. L-5b is distinguished from L-6α; L-4-6 has fewer fibers than in F44.<br>**Bistriate, euradiate, L-4 less intense than L-5b**
	Subregio propeunistriata	This SR shows a relatively wide and fiber-poor L-5a and a significantly darker L-6aα.	
**56**	A. propeunistriata (44 BA)	B; H68-ext; VV	Trizonal. L-4-6, especially L-5a,b display few fibers. L-1a+b and L-3-5b (particularly L-5a) have fewer fibers than in F59. In L-4-5b significantly less fibers are noted than in F55a and F58.<br>**Bistriate, euradiate, L-4 less intense than L-5b**
6. R. UNITOSTRIATA Areae 57-66		Of the two SR the *Subregio subunitostriata* shows significantly fewer fibers in L-5a. (F56-66: Broca-Region; see Kreht, 1937a: cytoarchitecture).	

(Continued)

**57-62**	***Subregio subunitostriata***		Trizonal. In this subregion L-5a shows less contrast. The Ef are coarser in character.
	Div.propebistriata		In the *Divisio probebistriata* L-4 and L-5b are separated by a light L-5a; L-6aα is brighter than L-5b.
**57**	A. propebistriata (≙ BA44b)	S1; S2Ph; VV	By the much lighter substriate L-6a (compared with F46) this field is distinguishable from the surrounding fields. Braitenberg (1956 p. 193) localizes this field on the *Pars ascendens* in the inferior frontal gyrus. L-1a+b is somewhat richer in coarse Ef, L-6α shows fewer Gf than F56. **Bistriate, euradiate, L-4 less intense than L-5b**
	Div.subunitostriata		The division appears dark due to the many Ef. The higher fiber content of the intrastriate lamina compared to the substriate lamina shows the L-4-5 as a uniform wide stripe.
**58**	A. dives	Br; H66-ext S2Ph; VV	L1a+b shows much fewer fibers than in F59. L-4 is weaker than L-5b. Gf and coarse Ef in L4-6 are more numerous than in F59. F58 is richer in Ef in L-4-5b (especially 5a) than F55. **unitostriate, euradiate**
**59**	A. pauper	Br; H68-ext; S1; S2Ph	Trizonal. Very similar to F65, on average probably somewhat poorer in Ef (therefore brighter) than F65. L-5b has somewhat fewer fiber and is narrower than in F65. L-4 is significantly less developed than L-5b and thus weaker than L-4 in F65. F59a contains significantly more Ef in L-5a than F54a,b and F53, but fewer than L-5a in F58 and F59. L-4 is somewhat weaker than L-5b in F59a. **unitostriate, euradiate**
	Div.multostriata		L-3 is very rich in fibers, but in the upper part of L-3a there is even a special accumulation of stripes (including of Kaes-Bechterew).
**60**	A. multostriata	Br; S1; S2Ph	The most fibrous field of the whole inferior frontal gyrus (both horizontal stripes almost completely merged). Trizonal. L-1a+b are very rich in fibers with numerous relatively thick Ef. L-1c, 2 are rich in fibers but with only few Ef; L-2 is well defined. L-3-6 are highly fibrous; L-4 = 5b. L-5a contains only slightly fewer fibers than L-4, 5b, 6α. **unitostriate, euradiate**
	Div.propeunistriata		L-6aα contains more fibers than all other divisions of the *Subregio subunitostriata*, thus heralding the transition to the neighboring unistriate type of the fields 5 and 9.
**61**	A. dives	S1; S2Ph	F61 differs from the F5 by the presence of coarse Ef in L-4 and 5a. F61 closely resembles F62 with respect to structure and fiber content. Subquadrizonal; L-1 here has much less fibers than in F62. In L-2,3 hardly any Ef are noted. **bistriate, euradiate, *L-4* = *L-5b***
**62**	A. pauper	S1; S2Ph	Differs from the F-61 by the more numerous Ef, but a smaller amount of Gf, especially in layers 4 and 5b. Trizonal (1a+b about as fibrous as in F-59. L-5b has fewer fibers than in F-63, and is therefore somewhat less distinct from 6α, but is by no means uniform. Weakly unitostriate. but L-5a fewer fibers than in F-63. Transition the unitostriate area and F-9 and 5. **bistriate, euradiate, *L-4* = *L-5b***
**63-66**	***Subregio unitostriata***		Stepwise increase in the overall fiber abundance from 63→64→65. Increasingly stronger unitostriate character. L-5a hardly stands out.
	Div.trizonalis		L-5a can not be defined in any of the three fields. F63 is very poor in Gf, so that the If predominate in L-4-5b. F-64 shows a augmentation of the If. F-65. further increase in the number of If as compared to F63 and F64.

(Continued)

**63**	A. pauper	S2Ph; 63	Trizonal. L-1a+b as rich in fibers and its Ef are as coarse and numerous as in L1a+b in F60 and F65. L-2 and 3 are like in F65. L-4 is hardly distinct from L-5a. L-4-6 has fewer fibers (especially Ef) than in F65. L-5b is clearly richer in fibers than L-4 and L-5a.<br>**unitostriate, euradiate**
**64**	A. intermedia)	Br; H68-ext, S1; S2; S2Ph, Str	Increase in numbers of Ef. Very similar to F63 and F65. It has more and somewhat coarser Ef in L-4-6 than F63, fewer Ef in L-4-6 than in F65. Otherwise (1-3) as in F63 and F65.<br>**unitostriate, euradiate**
**65**	A. dives	Br; S1; S2; S2Ph	Trizonal. F65 is fibrous and shows an additional increase in Ef. In contrast to F58 (and thus also in difference to F57 and F59), coarser and more numerous Ef are found in layers 1a+b and 4-6, but also some Gf. Compared with F60 this field shows somewhat fewer fibers in L-1a+b, particularly in L-3-6. L-5a has fewer fibers than in the lateral part of F65 .<br>**unitostriate, euradiate**
	Div.propebizonalis		
**66**	A. propebizonalis	Br; S2Ph; VV	Structurally, F66 forms a transition between F63 and the allocortex (between the euradiate and the supraradiate cortex). It exhibits a different lamination pattern (Braitenberg, 1956 p. 195) with strong radial fibers reaching into the upper layers of the cortex (up to L-1c). Trizonal. L-1a+b is very rich with respect to coarse Ef; even richer in this respect than F63. In L-1c numerous Ef that stand narrower than in F-65. L-4-5b forms a uniform band of the fibers. 6α hardly has fewer fibers than L-4-5b.<br>**unitostriate, euradiate**

Annotation: Hopf [40] could not define the fields 3,7,29,40,46 with certainty.

Abbreviations: aequilatus=equilateral (with equal sides); B1=outer Baillarger stripe (Gennari stripe; IV. Lamina granularis); BA=Brodmann area; B2=inner Baillarger stripe (layer Vb); BA=Brodmann area; Ef= individual / single fibers (*Einzelfasern*); mostly horizontally oriented; F=field; Fb=f*iber bundle*; Ff=ground fibers (*Faserfilz*); Gf=ground fibers; basic fiber network (*Grundfasern*); Gr=Griseum; Hf=horizontally oriented fibers (*Horizontalfasern*); L=layer, sublayer; Pre = Lamina principalis externa; Pri=Lamina principalis interna; Rb=radially oriented fiber bundle (Radiärfaserbündel); Rf=radially oriented fibers (*Radiärfasern; Radiäreinzelfasern*); Sf=oblique fibers; (*Schrägfasern*); Srf=supraradiary fibers (*Supraradiärfasern*); Tf=tangential fibers (*Tangentialfasern*).

References: see text

SUPPLEMENT 2: Isocortex Parietalis and Cortex Insularis

The first column shows the numbers of the myeloarchitectonic fields distinguished in the parietal (Fields 67–96) and insular (Fields 97-102) cortex. We differentiate between myeloarchitectonic “Fields” (F) and cytoarchitectonic “Areas” (A). The next three columns show the hierarchical order from Subregio to Area (Field). The next column indicates where drawings or photographs (Ph) of the myeloarchitectonic cortical structure can be found (Ba: Batsch [94]; Br: Brockhaus [103]; H69: Hopf [28]; H70:Hopf [29]; V11: Vogt [62]; VV: Vogt and Vogt [16]). The last column only provides rough information about the typical and differentiating features of the individual field. A list of abbreviations can be found at the end of this table. References can be found in the main text.

I. Regio euradiata		Sub-Areae	Figures	Comments
**Area**	***α) Subregio eucingulata .***		The most important distinguishing features relate first, to the changing abundance of medullary fibers and second, the length of the Rf as well as the number and ratio of the Hf (Rose [113]). According to varying degrees of the eucingulate character this subregion can be divided into an **eucingulate** and a **subeucingulate** division.	
	**αα) Divisio eucingulata**		The *Subdivisio reuniens* mediates the transition between the parietal isocortex and the frontal Isocortex. It can be distinguished from the caudal *Subdivisio typica by* is less sharply distintion of L-5b from L-6a.	
	ααα) Subdivisio reuniens			The anterior slope of the posterior central gyrus is to be thought of folded up to the front. In this way, fields F67 and F69, which are otherwise hidden in the central furrow, are shown on the surface map. Only on the medial side do both fields reach the surface.
**67**	A. dives	67 I-IV	Ba_Ph, H70, V11,	Compared to F42, this area shows thicker Ef in L-4-6, but at the same time much fewer Gf in L-2, L-5a and L-6aα and smaller numbers of radii. F67 is therefore well distinguished from F42 by a eucingulate and bistriate character.<br>Batsch [94] divided this field into four subareas, of which F67-1 is bistriate, aequodense and externolatior; F67-2 is **bistriate** and aequodense; F67-3 can be described as bistriate, externodensior and externolatior and F67-4 is bistriate, aequodense and externolatior.
**68**	A. pauper	68 I-III	H70; V11,	This field is part of the parietal operculum (F68, F73, F74) following the prefrontal F41 (Strasburger) but regarded as part of the central posterior region, since these fields represent the continuation of the postcentral fields downwards.<br>F68 is one of the most fibrous fields in the entire parietal area. At the lateral part it forms a transition to the field F72. Batsch [94] divided F68 into three subfields: F68-1 is propeunistriate, merges at the superior marginal sulcus into F100 of the insula, which has fewer fibers; F68-, lateral to 68-1, forms the main part of F68. It is richer in fibers than F68-1, also propeunistriate and aequodense. F68-3 is still propeunistriate and aequodense (Vogt: aequifascial, dysfascial (-fasciate), equilateral). It is more fibrous than the other subfields but contains less Gf than F67; the radial bundles are particularly striking; these are intense, but thin and mixtoradiate. with only slightly narrower L-5a, with many but relatively thin individual fibers in L-4-6. The two Baillarger stripes stand out somewhat more clearly

(Continued)

				expressing the transitional character of this subarea to the bistriate fields is. H70: **propeunistriate**
	ααβ) Subdivisio typica		The opercular section contains- like the *Divisio reuniens* - thinner individual fibers than the more dorsal parts. Vogt distinguishes the tenuifibrose opercular fields (F72-74) from the parietal grossofibrose fields (F69-71).	
	aaßa) Areae grossofibrosae (Vogt, 1911)		Three well-defined fields can be distinguished here in the oral-caudal direction; the most oral field L-5b is much richer in fibers, the middle L-4 and 5b are equally rich, in the most caudal F4 is somewhat richer than L-5b.	
**69**	A. paradoxa (internedensior)		Ba; H70; VV, V11,	F69 is one of the most prominent fields of the posterior central gyrus. It occupies the oral wall and adjoins F67-2 in the base of the sulcus.
	A. grossofibrosa paradoxa (Rose)			The considerable increase in caliber and the number of thick individual fibers distinguish F69 from F67; from all other areas of the *Regio euradiata* it differs by the particularly fiber-rich L-5b. **bistriate, internedensiate and eucingulate**
**70**	A. aequidensa	70 I, II, med	Ba; V11,	F70 occupies the angulus and a small portion of the surface of the postcentral gyrus. It exhibits a considerable reduction in the very thick individual fibers. L-5b is now not darker than L-4.
	A. grossofibrosa aequidensa (Rose)			**bistriate, aequodensiate and *externolatior* with noticable KB**
**71**	A. externedensior	71-I, II, med	Ba; H70; V11,	The very thick individual fibers have completely disappeared. L-4 is slightly darker than L-5b. L-5a is significantly brighter and wider than in F70.
	A. grossofibrosa externidensior (Rose)			***bistriate, externodensiate and externolatior with noticable KB***
	ααββ) Areae tenuifibrosae (Vogt, 1911)		Two relatively different areas are distinguished: The outer corresponds to F71 but with fewer fibers. The internal area, adjacent to the *insula* has a very different structure. It is much more eufascial and tenuifascial and also has a delicate but well-characterized Kaes-Bechterew stripe.<br>This area thus approaches the neighboring fields of the temporal isocortex , but can be distinguished quite easily from the latter by a clearly fewer fibers in L-1a+b.	
**72**	A. (tenuifibrosa) eucingulata		Ba; H70; V11,	F72 occupies the lower third of the postcentral gyrus (Cp), adjoining caudally F71 1 and forming a transition from both postcentral to inferior parietal parts, including the parietal operculum. It has fewer fibers than the post-central fields and has about the same fiber density as F68-2, which is close to the opercule.<br>Compared to the adjacent F68, it is more tenuifascial and eufascial and is richer in fibers with L-5b clearly contrasting L-5a and 6aα. Compared to F71, this field has fewer basic fibers.<br>***Still bistriate, externodensiate and externolatior***

(Continued)

**73**	A. (tenuifibrosa) multstriata dives	73 I-III	Ba; H70;	Well separated from F68 and F72 by the multostriate character. L-3a breaks down here into a loosely built L-3aα, formed largely of relatively thin individual fibers that are sufficiently well characterized by their caliber, and a denser L-3aβ, consisting predominantly of basic fibers. Pronounced eu- and tenuifascial. At the *S. marginalis superior* it merges into F101 of the insular isocortex, which has a much smaller number of fibers. **propeunistriate and slightly externolatiate**
**74**	A. (tenuifibrosa) multstriata pauper	74 I,II		Significantly fewer fibers, otherwise structurally similar to F73. At the *S. marginalis superior* it merges into F103 of the temporal isocortex, which can be distinguished immediately by a more fibrous L-1a+b. Batsch [94] divides F74: 74-1 that has less fibers and is almost bistriate, while 74-2 has fewer fibers and shows almost no lamination. **propebistriate and aequodense**
	**αβ) Divisio subeucingulata**		L-2 is less distinct from L-1c and L-3a than in the parietal types described so far. Also different from F7 due to much fewer Gf and Ef.	
**75**	A. subeucingulata (Vogt, 1911)	75-if, sup,	Ba; H70; V11,	F75 is located in the dorsal area at the transition from the Cp to the intermediate and superior-medial parietal subregions. Batsch [94] distinguishes a lower section, F75-if, that is pronounced bistriate and aequodense and an upper section, 75-sup, being beautifully **bistriate** and aequodense. .
	ß) Subregio dyscingulata		Vogt [62] distinguishes five division of unequal size according to the behavior of L-5a and 6aa.	
	**βα) Divisio unistriata**		This solitary field is surrounded by decidedly bistriate types and is particularly striking at this point.	
**76**	A. unistriata	76-if, sup,	Ba; H60; H69; V11,	F76 was described by Vogt as different and falling out of the surroundings. It is the only field in the parietal lobe that has a unistriate cortex. It lies between the *Subregio parietalis cingularis* and F84, which is between F76 and 85. F76 is divisible into a lower and upper section. F76-if borders on the calcarine fissure and forward to allocortical retrosplenial fields. F76-if is unistriate with a quite prominent outer Baillarger stripe. F76-sup, which adjoins dorsally and does not reach the calcarine fissure, contains more fibers. F76-sup is unistriate, the outer Baillarger stripe is not as prominent as in F76-if. **unistriate**
	**ββ) Div. propeunistriata**		This division forms a transition area between the frontal isocortex and the parietal isocortex in the cingulate gyrus. It is already **bistriate**. L-5b, however, differs relatively little from L-6aα. It is also characterized by the fact that L-1a+b	

(Continued)

			does not differ from L-1c with respect to the fiber content, i.e. the division is **bizonal**. It consists of two easily distinguishable fields.
**77**	A. aequidensa		F77 is a small, fibrous field. The upper layers are not well developed, but the radii reach up to L-3. It is distinguished from F39 by the **bizonal** character, as well as by much fewer and thinner fibers, especially in L-4 6, and differing from F28 due to a generally greater abundance of fibers (dyscingulate character), by the significantly more radial bundles extending in L-3b and by the more horizontal fibers in L-4 and in L-5b. ***propeunistrite and*** **slightly internodensiate**
**78**	A. internedensior	Ba; VV,	It differs from F77 in that L-4 has fewer fibers than L-5b and that there are fewer Gf and thinner Ef. Compared with F20 and F24 this field shows more radial fibers in L-3 and more horizontal fibers in L4 and 5b. **propeunistrite and internodensiate**
	**ßγ) Div. bistriata**		Vogt divided the region into one field on the median side between the area formed by F76-78 together with the *Regio propesupraradiata* and the main section of the *Divisio bistriata* (therefore *Subdivisio intermedia)*. It is then followed by the distinctly bistriate *Subdivisio bistriata*. This occupies the main part of the praecuneus and the dorsal part of the superior parietal lobule.
	ßγα) Subdivisio intermedia		The intermediate character of this subdivision is revealed in the oral half by the fact that a narrow L-5aα is reminiscent of the propeunitostriate character of the adjoining *Regio propesupraradiata*, while a caudal half is decidedly bistriate. The oral half is less eu- and tenuifascial than the caudal .
	ßγ**ββ**) Areae subbistriatae		An oral field with few fibers may be distinguished from a caudal field with more fibers.
**79**	A. bistriata pauper	Ba; H60, H69	F79 connects caudally to F77 and 78. The two Baillarger stripes stand out a somewhat better than in F77 and 78. This field is, compared with F78, less dysfascial, equidense, with narrower L-5a, generally richer in fibers particularly in thicker individual fibers. Compared to F77 it is less dysfascial. L-5a shows more fibers whereas L- 6aα is less fibrous.
	Subdivisio intermedia (Rose, 1935)		**F79 is still propunistriate and slightly internodensior.**
**80**	A. subbistriata dives	Ba; V11,	F80 is significantly richer in fibers than the previously reported fields in this subregion. Also more eu- and tenuifasciar than F79, although the difference between 1a+b and 1c is still not great. **propeunistriate and aequodens**
	ßγαß) Area bistriata		The caudal half of the *Subdivisio intermedia* is formed by a single area.
**81**	A. bistriata	Ba; H60,H69	F81 is bistriate, pronounced eu- and tenuifascial. In L-1 and in L-4-6 numerous individual fibers stand out. It forms a transition to the adjacent fields. It is considerably more

(Continued)

				fibrous than F76 and shows some resemblance to the unitriate (H69) type.<br>**propeunistriate and aequodens**
	ßyß) Subdivisio bistriata .		This subdivision also falls into two well-characterized halves. The caudal half is not only characterized by a generally greater abundance of fibers. L-3-3b is so rich that 3b is only slightly behind L-4 in this respect. Accordingly, caudal areas subconjuncta is distinguished from oral areas sejunctae.	
	ßyßα) Areae sejunctae .		A small oral field can be distinguished from a large caudal one. The former mediates the transition to F80 due to the less trizonal character of its L-1 and the less fibrous L-4 and L-5b.	
**82**	A. sejuncta subtrizonalis		Ba	F82 lies on the medial side between fields 80 and 83 and forms a transition to the fiber-poor and propunistriate *Subregio par*acingularis *oralis*. L-1a+b is half as wide as L-1c, but only slightly different from L-1c, even if the difference between the two layers is somewhat larger than in F80. Compared to the F80, L-5a also has fewer fibers and is wider. F82 is immediately distinguishable from F75 by its dyscingulate character, but above all by the absence of the thick individual fibers in L-1-6.<br>**pronounced bistriate**
**83**	A. sejuncta trizonalis	83 I-IV	Ba, H60, V11,	F83 lies mainly medial; here it borders caudally on F82 and only extends with a narrow strip over the “Mantelkante” (*margo superior cerebri*). Well distinguished from F81 by the absence of thicker Ef in L-1 and L-4-6. A small field 83 1 contains more basic fibers in L-4-6 and thus approaches F75, but remains distinguished from this by its dyscingulate character and, above all, the absence of thicker individual fibers in L-4-6.<br>Batsch (1956) has distinguished four subfields: all bistriate, but 83-1 slightly internodense, 83-2 aequodense and slightly externolatior, 83-3 aequodens and 83-4 internodens.
	ßyßß) Areae subconjunctae		A small ventro-oral area can be distinguished from a large dorsocaudal area. The former is less bistriate, thus forming a transition to F76.	
**84**	A. subconjuncta propeunistriata		Ba	F84 is a small transition field on the medial side between the unistriate F76 and the bistriate F85. From F76 it is well separated by generally greater abundance of fibers, by greater prominence of L5b, and by particular abundance of fibers in L-3.<br>**propeunistriate**
**85**	A. subconjuncta bistriata	85 I-IV	Ba, H60, H69; V11,	F85 is the largest in the entire parietal area; it lies caudal to F83 and extends dorsally and medially to the parieto-occipital sulcus. F85 is fibrous throughout, **bistriate**, and has a fairly dark L-3-3. F85 differs from F84 by even more

(Continued)

				prominence of L-5b. Batsch (1956) divides F85 into four subfields, just like F83:<br>F85-1 is medially adjacent to F81, 83, 76, and 84. It is fibrous, bistriate, **externolatior**, slightly externodense and subconjunct .F85-2 borders F83 dorsally and medially, it is fibrous but appears somewhat lighter than F85 1. It is bistriate, **slightly internodense and subconjunct**. F85-3 forms the medio-caudal border field to the occipital lobe. L-4 is here not as clearly developed as in F85-1 and 85-2. L-5-2 has the same fiber density as L-4. F85-3 is bistriate and **aequodense**. F85-4 occupies the main part of the dorsal surface and the Mantelkante (superior cerebral margin) and is bordered by the parieto-occipital sulcus. It is more fibrous than the subfields previously described. F85-4 is bistriate and **aequodense**.
	**ßδ) Div. unistriata**		This division is divided into two fields: a large one, which only hints at the unitostriate character (a lamina intrastriata, L-5a, is still detectable, albeit narrow and fibrous), and a small one, which clearly stands out by its unitostriate character.	
**86**	A. propebistriata	86 I-II	Ba, H60, H69	F86 forms the transition of the Cp fields to the conjunctostriate F87. Well different from F83, 85 and 75 by the narrowness and abundance of fibers of the lamina intrastriata (L-5a). F75 is also eucingulate, containing thicker individual fibers in L-4-6. F83 contains thinner individual fibers and fewer fibers in L-3. F85 is richer in fiber, especially in L-3.<br>Batsch [94] distinguished the smaller propebistriate and internodense subfields wit fewer fibers and the larger and darker subfield that is propebistriate, possibly propeconjunctostriate and internodensior. H69: **bistriate**
**87**	A. unitostriata		H60, H69	F87 is almost completely surrounded by F86 caudally and is a small field that is not always easy to discriminate. F87 stands out from its environment due to its high fiber content and its **unitostriate** character. H70: **conjunctostriate**
	**βε) Divisio propeastriata**		This divisions differs from the last two ones in that L-6aα is barely visible. The pattern comes close to the astriate type. In an oral region, L-5a is as dark as L-6aα. The propeastriate character is thus more pronounced here. In the caudal region, L-5a is brighter than L-6aα. This division is therefore more **propeunistriate**.	
	βεα) Subdivisio typica		The subdivision contains 2 fields: F88 differs from F71, 72 and 73 by its discingular character and much thinner individual fibers in L-4-6. F89 contains fewer Gf and much fewer and thinner Ef than F86.	
**88**	A. dives	88a, 88p	Ba; V11,	F88 is the most oral and smallest area of the inferior parietal lobe. It extends to the parietal operculum and can be

(Continued)

				divided into the frontal F88a and the F88p, which is located somewhat above and on top of the operculum.<br>F88a has fewer fibers than F88p and slightly more fibers than the adjacent F73-3 in opercular direction. F88a is **propeastriate**.<br>F88p connects caudally to 88a and is generally richer in fibers. As the sublayers of the L-1 can be so well developed that one could speak of a **quadrizonal** type.<br>F88 is well distinguished from F72, 73 and 71 by its **dyscingulate** character and much thinner individual fibers in L-4-6. **propeastriate.** Batsch [94]: **conjunctostriate**
**89**	A. pauper	89a, m, p, i, e	Ba, H60,	F89 in the inferior parietal subregion, located in the middle of the lower parietal lobe, shows the least fibers. F89 connects caudally to F88 and is usually somewhat more extensive than F90. Like F88, it is propeastriate and on the whole has fewer fibers than the two adjacent fields F88 and 90.<br>F89 is more eufascial compared to F88 . It shows fewer Gf and, above all, much fewer and thinner Ef than F86. In a dorsal half, L-1a+b is somewhat wider and has fewer fibers. F89 can be broken down into a varying number of subfields (Batsch, 1956), but the subfields ventrally to the temporal lobe and dorsally to the intraparietal sulcus are quite constant. **propeastriate**
	βαβ) Subdivisio atypica		In the only field of this subdivision, L-5a is slightly brighter than L-6aα. The F90 differs from the F85 and 86 in that it has fewer fibers in L-6, much thinner individual fibers and a stronger tenuifascial structure.	
**90**	A. atypica	90a, m, p, Ip, t, o	Ba, H60, H69; V11,	F90 is the most caudal field in the inferior parietal lobe; it does not extend beyond the intraparietal sulcus. It differs from F86 and F85 because of its lack of fibers in L-3-6, much thinner individual fibers and a stronger tenuifascial structure.<br>In this field, too, a number of transition areas can be distinguished: to the intraparietal sulcus, to the temporal lobe and caudally to the occipital lobe (Batsch, 1956).<br>**propeunistriate to propeastriate**
**2. Regio (prope-) supraradiata** (Rose, 1935, p. 756)			This region is characterized by a relatively large number of thick Ef in L-1 and a relatively large number of radial bundles and few horizontal fibers in L-3. It is divided into two sub-regions, from which eusfascial and a dysfascial section is distinguished in the caudo-oral part.	
α) Subregio typica			Fields 91 to 93 scarcely come to the surface, while fields 94 to 96 occupy the surface of the cingulate gyrus and F94 can reach a quite considerable extent here.	

(Continued)

		The whole subregion is **propesupraradiate** (the fibers reach beyond L-3-3, partly into L-3-1). The proximity to the allocortex is evident by the distinctiveness of L-1.	
	**αα) Div. eufasciata**		
**91**	A. eufasciata	Ba, H60, H69; V11,	F91 wraps around the end of the corpus callosum and borders on the retrosplenial region, which belongs to the allocortex and is not counted as part of the parietal area. F91 presents in L-1a+b, but also in L-1c, a great number of coarse Ef. It is a type dyscingulatus and conjunctus. L-5a is clearly lighter than L-4 and also L-5b ,due to the narrowing of the radii and decreasing numbers of Gf, as well as of Ef. On the other hand L-5a is such dark that the area must be described as **subunitostriate**. L-6aa is significantly brighter than L-5a. **propesupraradiate, bistriate, internodense and subconjunct**
	**αβ) Div. dysfasciata**	The division can be divided into a trizonal and a bizonal field. The former exhibits less Gf in the 1st layer and especially L-1a+b than F91; in the bizonal field the difference between L-1a+b and L-1c is completely eliminated.	
**92**	A. trizonalis	Ba, H60,H69++	Most parts of F92 are buried. F92 is generally very rich in fibers but L-1a+b is less rich in Gf than in F91. **propesupraradiate, propeconjunctostriate, slightly internodensior and subconjuncta**.
**93**	A. bizonalis.	Ba	F93 is a small, narrow field on the cingulate gyrus that barely reaches the surface. It is very fibrous and propesupraradiate. The difference between L-1a+b and L-1c is completely eliminated. All L-1 sublayers have the same number (absolutely few) of Gf and also of Ef . **propesupraradiate, propeconjunctostriate, internodensior and subconjuncta**.
	ß) Subregio atypica		
	**βα) Div. eufasciata**	The only field of this division differs from F91 by a general lack of fibers and by a decrease in the number of fibers in L-1 and L-5a.	
**94**	A. eufasciata	Ba; V11,	F94 is located dorsocaudally to F91. In places it appears to be much less fibrous than the other fields in this group and also shows a bistriate character more clearly. F94 differs from F91 not only by the general lack of fibers, but also by the specific decrease in the fibers in L-1 and L-5a. **propesupraradiate, bistriate and aequodense**.
	**ββ) Div. dysfasciata**		
**95**	A. trizonalis	Ba	F95 is dorsal to F92 and of about the same size as F96. Batsch (1956) describes it as **propresupraradiate** and still **propeconjunctostradiate**, although at times it looks almost bistriate.
**96**	A. bizonalis	Ba	F96 lies dorsal to F93 and is a fibrous, small field. **propesupraradiate, propeconjunctostriate, internodensior and subconjuncta** .

(Continued)

**Insula**	Vogt and Vogt (1919) designated the isocortical portion of the insula as areas i1–i6 or areas 97–102. Brockhaus [16] assessed the parcellation results of C. and O. Vogt [16] (myeloarchitecture) and Rose [102] (cytoarchitecture) as fundamentally different and produced a comparative analysis, also taking into account the studies from Brodmann and from von Economo and Koskinas. His account is extremely detailed: https://www.thehumanbrain.info/brain/db_literature/BrockhausCortexclaustralis.pdf).			
	Insula anterior			
**97**	**I 1**	*G. brevis primus*	*Br*	**Eufasciate, trizonal, dyscingulate, poorly bistriate, internodensior, medioradite**
**98**	**I 2**		*Br*	**Generally lower in fiber than I-1; somewhat stronger eufasciate, dyscingulate, propeunistriate, nearly aequodense..**
**99**	**I 3**	*G. brevis secundus*	*Br*	**Dysfasciate, trizonal, unistriate, from denso- to medio- and tenuiradiate.**
**100**	**I 4**	*G. centralis anterior*	*Br*	The propeunistriate F68-1 merges at the superior marginal sulcus into F100, which has fewer fibers Batsch, 1956). **Eufasciate; trizonal, uni- striate, internodensior, tenuifibrate, medioradiate .**
	Insula posterior			
**101**	**I 5**	*G. centralis posterior primus*	*Br*	**Eufasciate, trizonal, unistriate, tenuifibrate, medio- and finoradiate .**
**102**	**I 6**	*G. centralis posterior secundus*	*Br*	F102 is adjacent to F103 which lies in the ventral lip of the posterior Sylvian sulcus of T1. Very poor in fibers. **Eufasciate, trizonal, pauper, unistriate, markedly tenuifibrate, tenui- and finoradiate.**

Annotation 1: Batsch [94] (p. 230) divided the parietal lobe into two regions, the *Regio centralis posterior* that includes the posterior central gyrus, the parietal operculum and some fields lying medially on the paracentral lobule and the larger *Regio parietalis* that includes the actual parietal lobe, including the part of the cingulate gyrus that is considered to be the transition to the allocortex. These two large areas are divided into eight subregions, two in the postcentral region (*Subregio postcentralis* [Area 67, 69, 70-72,75]and *Subregio opercularis* [Area 68, 73, 74]) and six in the parietal lobe proper (*Subregio parietalis inferior* [Area 86, 87, 88-90], *Subregio parietalis superior-medialis* [Area 82-85], *Subregio parietalis paracingularis oralis* [Area 77-80], *Subregio parietalis paracingularis caudalis*[Area 76, 81, 91-96]).

Annotation 2: C. and O. Vogt [16] depicted (Fig. 7) also allocortical insular divisions: *Insula ventralis* (ai1, ai6); *Insula olfactoria* (ai2-ai4); *Area praepiriformis* (ai5). Rose [102] used this terminology in his cytoarchitectonical study of the human insula.

Abbreviations: aequilatus=equilateral (with equal sides); B1=outer Baillarger stripe (Gennari stripe; IV. Lamina granularis); BA=Brodmann area; B2=inner Baillarger stripe (layer Vb); BA=Brodmann area; Ef= individual / single fibers (*Einzelfasern*); mostly horizontally oriented; F=field; Fb=fiber bundle (*Faserbündel)*; Ff=ground fibers (*Faserfilz*); Gf=ground fibers; basic fiber network (*Grundfasern*); Gr=Griseum; Hf=horizontally oriented fibers (*Horizontalfasern*); L=layer, sublayer; Pre = Lamina principalis externa; Pri=Lamina principalis interna; Rb=radially oriented fiber bundle (Radiärfaserbündel); Rf=radially oriented fibers (*Radiärfasern; Radiäreinzelfasern*); Sf=oblique fibers; (*Schrägfasern*); Srf=supraradiary fibers (*Supraradiärfasern*); Tf=tangential fibers (*Tangentialfasern*).

SUPPLEMENT 3: Isocortex Occipitalis

The first two column show the consecutive numbers of the parceling scheme proposed by Nieuwenhuys and Broere [22] and the field names by Lungwitz [40] for the pre-occipital region (A 18). The next column indicates where to find figures characterizing the myeloarchitectonic cortical structure of each relevant field (EK: von Economo and Koskinas [63]; L=Lungwitz [40] [figure number]; VV: Vogt and Vogt [16] [figure number]). This numbering system leaves no room for the occipital (A 18) and striatal (A17) subregions. We have therefore interposed numbers starting at 200. The list is followed by the (modified) table with the classification of the pre-occipital fields according to Lungwitz [40]. A list of abbreviations can be found at the end of this supplement. References can be found in the main text.

**Isocortex occipitalis** Fields 103-119			The radii are medium wide and have thicker Ef, L-1a+b is very narrow and shows a much greater abundance of basic fibers than L-1c. The lamina dysfibrosa (L-2) is notable for its lack of fibers. Otherwise it is a subconjunctostriate, externodenseior and externolatior cortex.
Subregio* (*A.) prae occipitalis (Pro)		VV[23, 32], EK137	*Area 19 (Area praeoccipitalis Brodmann, 1909), Area peristriata OA (von Economo and Koskinas, 1925).*<br>The radii are medium wide and also contain thicker Ef; L-1a+b are very narrow and exhibit much more Bf than L-1c. The lamina dysfibrosa (L-2) is notable for its lack of fibers (Rose [113]).<br>Vogt and Vogt [16] published two diagrams with somewhat differing classifications of “Area 19”. Fig. 23: “Infra-, barely lato-, modico- and mixto-radial. Propetrizonal, weakly ultratangential, internodyscingular. Pauper, unisingulo-fibrous, separatus; tenui-intrastriatus, intimolatior, tenui¬limitatus”. Fig. 32: “Eu-, medio-, reasonably denso- and mixtoradial. Trizonal, eu- and strongly tenuifascial, multo- and slightly crassofibrous, euiangential and eucingulär. Weakly trisingulofibrous, separatus. Weakly grossofibrous, subconjunctostriate, externodensior, externolatior, tenui-intrastriate, intimolatior, tenuilimitatus” (see also von Economo and Koskinas [63], p. 622). Rose [113] emphasized the subconjunctostriate, externodensior and externolatior character.
**103**	**edl**	L[13]	Location: base. Medial to S.l1 and calcarine fissure; bounded orally by temporal fields, laterally by S.ot (**edt**), caudally by S.l2 (**eda**).<br>L-1° fiberless. L-1a+b dense; strong Tf; as wide as L-1°. L-1c wider than L-1° to 1b. Ff denser than in **edt**. Fine caliber. Single Hf of stronger caliber. Wider than L-1° to 1b.<br>L-2 narrow; well demarcated against L-3aα and 1c<br>L-3aα slightly denser Ff than in L-1c. As wide as L-1c. Contains fine processes of the radii. L-3aβ somewhat denser with splitting radii. Fine Hf. Almost of the same width. L-3b only slightly denser than L-3aβ. Hardly wider than L-3aα.<br>L-4 slightly wider than L-3b. Very dense Ff with Hf. Only few and very short Sf.<br>L-5a brighter than L-4. Ff quite dense, well marginated. Long Hf of stronger caliber. Occasionally Sf. L-5b quite dense, hardly different from L-5a.<br>L-6α to 7ß slowly merge in terms of the density of their Ff. L-6α is barely brighter than L-5a. In the last two laminae long Sf of thick caliber extending over several layers; also Hf. Radii predominantly bundled; fewer Ef.<br>**edl** is distinguish from:<br>1). Area occipitalis (**O)** by pronounced L-4 and brighter L-5a and L-7α.<br>2). **edt** by darker Ff; less pronounced L-4; denser L-5b.<br>3). **eda** by lighter Ff; less sharply defined L-4; denser arrangement of the radii.
**104**	**eda**	L[12]	Location: base. Bounded medial and caudal by **O** and S.l2 (**edl**), medio-oral by ot (**edt**), lateral by ot (**elt**).<br>L-1° few fibers. L-1a+b dark; as wide as L-1°. Occasionally Tf.<br>L-1c as wide as all previous strata. Dense fiber plexus.

(Continued)

			L-2 stands out well; few fibers. As broad as L-1° to 1b.<br>L-3a slightly wider than L-1° to 1c. Ff about the same density as in L-1c. Ample Hf of stronger caliber. L-3b about as wide as L-3a but darker. More and stronger Hf.<br>L-4 slightly wider than L-3a. Very dense Ff with Hf. Poor contrast against L-3b but quite noticeable against L-5a. L-5a bright Ff with Hf. Half as wide as L-4. L-5b is poorly demarcated; only slightly darker than L-5a; strong Hf; quite wide.<br>L-6α and L-6ß can hardly be separated. The former as bright as L-5a.<br>7 α and 7ß with increasing Ff density. Hf with widened caliber. Some Sf.<br>Differs from<br>1) **O** by slightly lighter Ff; the low number of Hf; by the separate L-4.<br>2) **edt** by denser Ff; separate and dense L-4; poorly visible L-5b.<br>3) **elt** by sharply defined L-4 and L-5a; less wide L-1.
**105**	**edt**	L[11]	Location: base. Bounded orally by temporo-basal fields, medial by S.ot (**edl**), lateral by S.if1 (**eld**), caudal by S.ol (**eda**).<br>L-1° fiberless; narrow. L-1a+b not very dark; few Tf; wider than L-1°. L-1c wider than L-1° to 1b, more dense; fine fiber feltwork (Ff)<br>L-2 less dense ground fibers (Gf). No Hf; fairly wide.<br>L-3a contains occasional endings of radial fibers with very fine caliber. Ff like in L-1c. About as wide as in L-2. L-3b darker Ff. Few Hf. As wide as L-3a. Splitting of the radii.<br>L-4 marginally wider than L-5a. Ff more dense. Few and longer Hf of medium caliber. Stands out well from the adjacent laminae.<br>L-5a slightly narrower than L-4, lighter Ff. Very few Hf. L-5b nearly as wide as L-4. Ff are hardly denser in than in L-5a.<br>L-6α as bright as L-5a; just as wide. 6β only slightly darker; wider.<br>7α denser Ff. Not very wide; hardly any single fibers (Ef). No Sf<br>Radii narrow sized; some already ending in the L-4.<br>**edt** differs from:<br>1) **eld** by narrower and darker L-4; brighter L-5b; overall darker Ff. Radii stand closer together;<br>2) **elt** by significantly narrower L-4; wider L-5a and 5b; overall darker Ff.
**106**	**elt**	L[9]	Location: base. Bounded orally by S.f_1s (**eld**), caudally by f_2s (**elsc** and **edpc**), medially by S.ot (**eda**).<br>L-1° fiberless, narrow. 1a+b dense; with Tf; as wide as L-1°. L-1c brighter Ff, broader than L-1° to 1b; few Tf.<br>L-2 slightly narrower than L-1c; few fibers.<br>L-3a hardly brighter than L-1c; broad. L-3b only slightly darker; occasionally Hf. Slightly narrower than 3a. splitting of the radii,<br>L-4 darker than L-3b stands out enough; contains longer and thick caliber Hf; as wide as L-3a.<br>L-5a narrower than L-4; bright Ff; hardly Hf. Not as wide as L-3b. L-5b stands out only slightly from L-5a; slightly darker; rather long and rather dense Hf.<br>L-6α only slightly brighter than L-5a; narrower. Isolated Hf and short Sf. L-6β darker Ff; wider than L-6α.<br>L-7α increasingly dense Ff; increasing amount of Sf.<br>Radii extend up to L-4 and 3b. Ef end in L-4.
**107**	**eld**	L[10r]	Location: base. Medial of S.if_1 (**edt**); oral: partly bounded by f_1s (**elt**), lateral by **pt** and on the convexity bounded ventrally by S.otl (**sct**).

(Continued)

			L-1° fiberless; narrow. 1a+b brighter than in adjacent fields; narrow; long Tf. L-1c scarcely wider than L-1° to 1b. Isolated Hf. Medium density. Twice as wide. L-1c marginally broader than L-1a + b .Some Hf. Slightly darker than L-3a.<br>L-2 very poor in fibers; well demarcated; narrow.<br>L-3aα Ff about as dense as in L-1c; , wider than L-1c. sporadic Sf. L-3aß delicate endings of radii; Ff marginally less dense. Ff in L-3b appear slightly darker than L-3a; more Hf. very few Sf Not as wide.<br>4 narrow; fairly dense Ff. Some medium-caliber Hf. Stands out well against L-5a.<br>L-5a much lighter than L-3b; not as wide as L-4. Some Hf; hardly some Sf. L-5b almost the same appearanse as L-4; slightly darker.<br>L-6α as bright as in L-5a, broader; sporadically Hf. L-6ß slightly darker.<br>L-7ß can still be defined.<br>Radii are clustered; few radiary Ef that splinter in the L-3b to 3a.<br>**eld** differs from **elt** by slightly darker Ff; slightly denser L-1; less well defined L-5b.
**108**	**pt**	L[10l]	Location: Base extending into the convexity. Bounded orally by the descending S.otl from the temporobasal and temporal fields; caudally from **eld** and dorsally on the convexity from S.otl (**sct**); in direction from the calcarine fissure by if_1 (**edt**).<br>L-1° fiberless. Wider than in the adjacent fields. 1a+b plenty of Tf of stronger caliber. Almost as wide as L-1°. L-1c wider than L-1° to 1b. Hf of smaller caliber. Somewhat lighter.<br>L-2 wider than in **eld**. Very few fibers.<br>L-3aα rarely any Ff. Marginally darker than in L-1c. 3aß delicate apical processes of Rf. Ff as in L-3aα. L-3b more Ff. Not very dense. Fine-fibrous radii.<br>L-4 nearly as wide as 3b. Dense Ff. Clearly distinguished from L-3b and L-5a. Thin Hf. Single short Sf. L-5a well contrasted. Lightish Ff. L-5b not as dense as L-4. Contrasts well with L-5a and 6α. As wide as L-4.<br>L-6α brighter than L-5a. Very small. L-6ß denser than L-4. Few Ef and Sf.<br>Radii not very dense. Hardly any Ef. Mostly bundled.<br>**Pt** differs from:<br>**eld** in having a smaller layer width; less pronounced L-4; somewhat denser L-5b; brighter L-5a. Approaches the temporal type.
**109**	**elsc**	L[(15]	Location: base. Bounded caudally by **O**, laterally by **sct**, orally by $S.f_2s$ (**elf**), medially by $S.if_2$ (**edpc**).<br>L-1° fiberless. As wide as L-1a+b. L-1a+b cannot be separated. Dense, short Tf of strong caliber. L-1c can be clearly distinguished from L-1a+b; nearly twice as wide as L-1° to 1b. Fairly dense, contains Hf of only slightly greater caliber than Ff.<br>L-2 not much wider than L-1° to 1b; few fibers.<br>L-3a is divided into 3aα and 3aß. L-3aα as wide as L-1c. Ff somewhat denser than in L-1c with strong caliber 1 Hf with a. Mainly horizontally oriented. No radiary Ef.<br>L-3aß slightly lighter Ff than 3aα; broader; contains the fine splitting of radii. L-3b dense Ff with brush-like splintering radii; fewer Hf than in L-3aα. These of lesser length. Same caliber as 3aß.<br>L-4 stands out well with its dense Ff. Slightly wider than L-1° to L-1c. Richer in Hf than L-3b. Stronger caliber. Termination of most radial Ef of thicker caliber.<br>L-5a sharply defined against L-4, much brighter. Hf almost of the same caliber as L-4; lower number. Narrower than L-4. L-5b slightly brighter than L-4. Slightly darker than L-5a; narrower; fine caliber Hf. Ff in L-6α almost as dense as in L-5a. Hardly be separable from L-5b and 6ß. Hf more plentiful of variable thick caliber. Sf coming from L-7ß of the same caliber.

(Continued)

			L-7α differs from L-6ß.<br>Radii not very dense. Bundled up until L-4. In addition, coarse and fine caliber radial Ef. These usually end in L-4.<br>**elsc** is distinguishable from<br>1) **elt** by more pronounced L-4; less dense and wide L-5b; not so dark Ff.<br>2) **edpc** by lighter Ff; few Tf and Hf in L-1 and 3; better recognizable L-5a; less distinct L-2.<br>3) **eld** due to broader and brighter L-3; significantly narrower and denser L-4 and L-5b; increased Sf.
**110**	**edpc**	L[14]	Location: base. Bounded caudally by **O**, laterally by S.if_2 (**elsc**), orally by S.f_2S (**elt**), medially by S.ol (**O**).<br>L-1° very narrow; fiberless. L-1a+b very dark; slightly wider than 1°. Tf with rather thick caliber. L-1c very broad; dark Ff; few Hf.<br>L-2 few fibers; half as wide as L-1c.<br>L-3aα broader and more dense than L-1c. Predominantly horizontally oriented. No apical processes of the radii. L-3aß with thick caliber Hf, rather long. Apical processes of the radii, intense Ff. L-3b not as wide as in L-3aß. Fewer Hf. Equally dense Ff as in L-3aß.<br>L-4 very dense Ff with Hf. Also Sf of stronger caliber.<br>L-5a darker than L-3b. Still stands out from L-4. Few quite long Hf. Half as wide as L-4. L-5b only slightly denser; slightly wider than L-5a few Hf.<br>L-6α coarse Ff; only slightly brighter than L-5a, short Hf; not as wide as L-3b. L-6ß darker Ff; darker than L-5b. Narrower than in L-6a.<br>L-7a of increasingly dense Ff.<br>L-6α to 7ß contain Sf of larger caliber extending over several laminae.<br>Radii stand very close together; thick caliber. Ef up to L-5b, diminished after L-5a, hardly any Ef in L-4.<br>**edpc** be distinguished from<br>1) **elt** by stronger Ff; broader and denser L-3; darker L-4; increased Hf.<br>2) **O** by broader, only slightly brighter L-1 and 3; apparent L-5a.
**111**	**dpp**	L[17]	Location: medial side. Almost only in the S.po, on the dorsal margin of the hemisphere encroaching on the convexity, caudally bounded by **del**, at the medial and convex surface by the S.os (**scet**); ob the concexity delimited from S.ip (parietal fields).<br>L-1° fiberless. L-1a+b narrow. Very dense. Plenty of thick caliber Tf of greater length. L-1c wide. Slightly brighter Ff. Abundant horizontal Ef and Sf of strong caliber.<br>L-2 as wide as L-1c. Ff somewhat loosened.<br>L-3aα predominantly Hf of considerable length. Some Hf of very thick caliber. Darker than L-1c.<br>L-3aß lighter and less coarse Ff. Hf recede compared to L-3aα. Fine caliber. No wider than L-3aα. Contains the radii ends. L-3b about as wide as L-3aß. Considerably denser Ff. 1 Hf and Sf almost like in L-3aα. Splitting of the radii.<br>L-4 wider. Well definable. Very dense. Many Hf.<br>L-5a less clearly defined. Fairly dark Ff. Little pronounced. narrower. L-5b quite dark. Almost as dark as L-4. As wide as L-5a.<br>L-6α as bright as L-5a. narrower. L-6ß and 7ß of increasing density of the Ff. Frequently Sf of large caliber.<br>Radii of thin caliber predominantly bundled.<br>Differs from **del** by being strongly crassofibrous L-3; lighter Ff; the better distinguished L-4 and brighter L-5a.

(Continued)

**112**	**del**	L[16]	Location: medial side. Bounded orally by S.po (**dpp**), posteriorly towards the S.calc by S,poc (**O**) caudally by **sct**.<br>L-1° fiberless. Narrow. L-1a+b narrow. Fewer and finer Tf. L-1c brighter than 1a+b. Significantly wider. Fine Ff<br>L-2 wide. Few fibers. Brighter than in **dpp**.<br>L-3aα weakly horizontally oriented. No radii endings. L-3aß splittings of radii. Some Hf. L-3b Ff more dense than in the two previous sublaminae. Brush-like splintering radii. Few Hf and Sf.<br>L-4 wide. Dense Ff. Occasionally Sf with thicker caliber than in **dpp**. Well distinguished from adjacent laminae.<br>L-5a clearly separated. Relatively dense Ff but less dense than in L-4. Scattered Sf Difficult to differentiate against L-5b. L-5b can be clearly distinguished from L-6α. Narrower than L-4. Ff slightly brighter than in L-4.<br>L-6α slightly brighter than in L-5a. Not wider. Contains Sf and Hf. L-6ß slightly darker. Contains Sf and Hf.<br>L-7α and 7ß of increasingly dense Ff.<br>Radii predominantly bundled. Fibers in L-3b. Thin caliber. Thicker radial Ef. Few Sf<br>Differs from **O** by its greater density of Ff; by its obvious L-4 and the light L-1 and 3; by the well-demarcated L-5a.
**113**	**pc**	L[20r]	Location: convexity. Defined dorsally and caudally by S.o1 (**scel** and **o**), orally by S.o2s and Gyr. O2 (**sced**), basally by S.o2 (**scd**) and S.oi (**sct**).<br>L-1° fiberless. L-1a+b dark. Heavily horizontally oriented fibers. Wider than L-1°. Poorly defined against L-1c. L-1c fine Ff. Approximately three times as wide as L-1° to 1b.<br>L-2 well demarcated. Few fibers. Not as wide as L-1c.<br>L-3aα darker than L-1c. Dense Ff. Small Hf with fine caliber. Sf of thicker caliber. Wider than L-1a+b. Contains the final branchings of the radii. Very thin caliber. L-3a ß almost as dense Ff. Hf somewhat longer and thicker. Very small. L-3b significantly denser. Hf and Sf less visible.<br>L-4 Hf thick and in larger numbers. Less clearly distinguishable from L-3b than against L-5a. As wide as L-3aα.<br>L-5a brighter Ff. Hf as strong as in L-L-4, but finer caliber. Narrow. L-5b slightly darker than L-5a. Densely packed, heavy-caliber Hf of greater length. Difficult to delimit.<br>L-6α slightly brighter than L-5a. Ff less dense. Narrow. L-6ß Ff denser. Otherwise same structure.<br>L-7α darker than L-4. Sf increase in caliber.<br>Radii densely arranged. Bundled. Stronger Ef. Partially ending in L-5b. Most fiber bundles terminate in L-3b and 3aß.<br>**Pc** differs from<br>1. **sced** by lighter Ff; low number of Hf and Sf; less dense radii.<br>2. **scd** by very bright Ff; loosened, weak and wide L-4.<br>3. **sct** by lighter Ff and denser L-4; Hf and Sf; rather small radii.<br>4. **O** by its extremo- and conjunctostriate fiber organization; density of the Ff.
**114**	**scet**	L[21]	Location: convexity. defined orally by the S.os (dpp) and the angular gyrus, caudally by $S.o_1s$ (**scel**), basally by $S.o_1$ (**sced**). Reaches over the superior margin of the hemisphere to the medial side to pm (O).<br>L-1° fiberless. As wide as L-1a+b. L-1a+b well defined. Narrow. Tf of small caliber. Not very tightly packed. L-1c slightly wider than L-1° to 1b. Well visible Ff. Hf and Sf.<br>L-2 nearly as wide as L-1c. Slightly brighter. Well represented Ff. Hf somewhat longer and of stronger caliber than Ff.

(Continued)

			L-3a darker and denser Ff. Darker than L-1c. Ample Hf of the caliber of the Ff and stronger. Very wide. L-3b dense Ff. Not as wide.<br>L-4 very wide. Dense Ff. Larger number of Hf with strong caliber.<br>L-5a very narrow. Stands out well from L-4 with bright Ff. Very fine Hf. L-5b equally dense Ff as L-4. Narrow. Slightly wider than L-5a. Thicker Hf and Sf.<br>L-6α and 6ß difficult to separate. Slightly brighter than L-5b.<br>L-7α and 7ß show increasing density. Hf and Sf of fairly large caliber.<br>Radii bundled. Ef. Tightly positioned. Splitting of radii in L-3b or 3a.<br>**scet** differs from<br>1. **dpp** by its very strong Ff; the strongly crasso-fibrous, horizontally oriented fibers in L-1.<br>2. **sced** by its overall lighter Ff; its wider radii; its less visible L-5b; due to lesser Hf.<br>3. **del** by its greater density; stronger Sf; denser and broader radii; broader L-4.<br>4. **pc** by lighter Ff; lighter, narrower L-5a; narrower L-5b; brighter and slightly broader L-4; as well as a significantly brighter L-3.
**115**	**sced**	L[20l]	Location: dorsally on the angular gyrus bounded by **scet**, orally by parietal area, basally by $S.o_2$ (**scd**, **pp**), caudally by o_2s (**pc**).<br>L-1° fiberless. Very small. L-1a+b not very dense Tf. Individual Tf with stronger caliber. Twice as wide as L-1°. L-1c not very dense Ff. Considerably wide.<br>L-2 Ff only slightly brighter than in L-1c. Narrower than L-1c.<br>L-3a equally dense Ff. Narrower than in L-3b; but somewhat brighter. L-3b dense Ff. Abundant Hf of short length.<br>L-4 as wide as L-3b. Significantly denser Ff. More Hf than in L-3b. Hf of greater caliber and medium length. Singular Sf of the same caliber in connection with L-5a.<br>L-5a well demarcated. Light Ff. Not as wide as L-4. Hf and Sf. L-5b only slightly darker. Only small Hf. Not very wide. Poorly distinguished against L-6α.<br>L-6α very narrow and bright. Hf and short Sf. Ff somewhat brighter than in L-3a. L-6ß somewhat darker Ff. Otherwise same structure.<br>L-7α and 7ß of increasing density. Plenty of Hf.<br>Radii are significantly less densely grouped than in **pp**. Radiary Ef with slightly thicker caliber. Fine-fibrous splintering of the bundles in L-3a and in 3b.<br>**sced** differs from<br>1) **pp** by its lighter and looser Ff; fewer Hf, especially in L-1; its denser L-4 and pronounced L-5a;<br>2) **scd** by its dense and sharply defined L-4; wider radii; brighter Ff.
**116**	**scd**	L[18r]	Location: convexity. Bounded dorsally by S.o2 (**pc**, **sced**), orally by to (**pp**), caudally by O_2S (**pc**), basally by S.oi (**sct**).<br>L-1° fiberless. L-1a+b narrow. Brighter than in **sct**. Fine Ff. Very fine Tf. Well demarcated against L-1c. L-1c slightly wider than 1a+b. Brighter than in set. No Hf.<br>L-2 very light. Fewer fibers than in **sct**.<br>L-3aα delicate Ff; same density and width as L-1c. L-3aß hardly darker than L-3aα. Almost the same width. Brush-shaped splitting of the radii. L-3b darker Ff.<br>L-4 approximately the same width as L-5a+b. Thick Ff. Well delineated. Occasionally Sf. No Hf.<br>L-5a wider and more contrasted than in **sct**. L-5b only slightly brighter than L-4. Almost as wide as L-5a. Some Hf of a larger caliber.

(Continued)

			L-6α and 6ß brighter than L-5a. wider than **sct**.<br>L-7α darker. Well demarcated.<br>Radii bundled; reach up to L-3aß. Bundles of varying width. Radially oriented Ef.<br>**scd** differs from<br>1) **sct** due to its somewhat denser Ff; more closely arranged radii; less pronounced L-5a, slightly wider L-3b
**117**	**sct**	L[18l]	Location: convexity. Bounded dorsally by S.oi (**scd**), orally by S.to, basally by S.otl (**pt**, **eld**, **elt**), caudally by **O**.<br>L-1° fiberless. L-1a+b sharply delimited from L-1°. Single Tf of stronger caliber. Slightly denser Ff. Slightly wider than L-1°. L-1c significantly brighter. Slightly dimmer than L-3aα. Broader than L-1° to 1b.<br>L-2 extremely low in fibers. About as wide as L-3 aα.<br>L-3a α narrower than L-1c. Ff like in L-1c. L-3aß slightly darker than L-1c. Narrow. Fine apical processes of the radii. L-3b darker Ff than in L-1c. Poorly demarcated from L-3aß.<br>L-4 sharply demarcated against L-3b. Approximately as wide as L-5a to L-5b. Occasionally Hf. Dense Ff.<br>L-5a narrow. Quite well differentiated from L-4. L-5b slightly wider. Not as dark as L-4. Both sublayers with short Hf.<br>L-6α and 6ß difficult to separate from each other. 6α slightly brighter than L-5a. No Sf. Some Hf of greater length.<br>L-7 α well discriminable. Darker than L-4. Very dense Ff.<br>Radii are tightly packed. Bundled up in L-3b. Few radial Ef which usually only extend to the 4th layer.<br>Differs from<br>1) **pt** by its slightly denser **Ef**; dense L-1 with more Ef; better identifiable L-5a; poorly visible L-5b.<br>2) **eld** by its dense Ff with greater width; better representation of L-5b; denser L-1.<br>3) **elsc** by the wider L-4 and narrower L-3; considerably wider and denser L-5b; overall darker Ff.
**118**	**scel**	L[22]	Location: convexity. Bounded orally by S.os (**scet**) and S.or (**pc**), medio-orally by **del**, caudally by S. occipito-transversalis (**O**)and dorsally by **pm**.<br>L-1° fiberless. L-1a+b very narrow; fibrous. Tf with very fine caliber. As wide as L-1°. L-1c very fine Ff; well demarcated against L-1b. Wider than L-1° to 1b. fine Hf.<br>L-2 narrow; almost as wide as L-1a+b; stands out well. Low fiber density.<br>L-3a slightly wider than L-2; Ff darker than L-1c; fine HF. L-3b denser Ff than in L-3a. More HF with stronger caliber and longer length. Wider than L-3a.<br>L-4 less well defined; Ff only slightly darker than in L-3b. About as wide as L-3b; more Hf with stronger caliber than L-3b but fewer than in **pc**.<br>L-5a very narrow; broader Ff. Single Hf. L-5b only slightly darker than L-5a; contains Hf of stronger caliber, not wider than L-5a.<br>L-6α slightly brighter than L-5a. Sf and Hf of finer caliber than in L-5b. L-6ß difficult to separate from L-6α; differs only by its low Ff density.<br>L-7α much denser Ff than in the substriate and the external limitans lamina. From L-7α to 7ß increasing numbers of Sf.<br>Radii stand close together; great numbers of radial Ef are present up to L-5b-5a which branch in L-3b. L-3a contains only very fine fibers.<br>**scel** differs from<br>1) **scet** by its subconjunctostrial character; lighter L-1 and brighter Ff.<br>2) **pc** due to its higher overall density of the Ff, especially in L-5.

(Continued)

			3) **O** due to its dense L-4 and clearly visible L-5a.<br>4) **del** by its loosened Ff, its barely recognizable L-5b; due to less dense radii.
**119**	**pp**	L[19]	Location: convexity. Bounded dorsally by S.o$_2$ (**sced**), caudally by S.to (**scd**), basally and orally by parietal fields.<br>L-1° fiberless. As wide as in **sced**. L-1a+b dense tangentially oriented fibers. As wide as L-1c. L-1c finer but relatively dense Ff. Hf.<br>L-2 narrow. Finer, somewhat denser Ff than in the adjacent fields.<br>L-3a somewhat denser than L-1c. Ff predominantly horizontally oriented fibers. L-3b denser than L-3a; narrower. Hf of stronger caliber.<br>L-4 rather dense Ff. As wide as L-1a to 1c. Larger numbers of thick-caliber Hf and Sf.<br>L-5a well delimited. Ff somewhat denser than in L-3b. As wide as L-3b. Some of the radial Ef end here. L-5b with Ff almost as dense as L-4. Much narrower than L-4. Hf with same caliber as L-4.<br>L-6α as pale as L-5a. L-6α to 7α denser than in the adjacent parietal fields. merge into each other. Sf and Hf in larger numbers.<br>Radii are more dense than in the parietal fields. Ef more frequent and up to L-3b.<br>**pp** differs from<br>1) **scd** by the narrower and brighter L-5a; denser and distinguished L-4; the slightly narrower L-5b.
Subregio* (*A.) occipitalis		VV[33]	*Area occipitalis (M. Vogt), Area 18 (Brodmann, 1919), Area parastriata, OB (von Economo and Koskinas, 1925).*<br>The medium sized radii occur in this subregion in particularly large numbers. L-1a+b is just as narrow as in the pre-occipital area; it may contain very thick individual fibers. L-2 and the Kaes-Bechterew stripe are clearly visible. L-5a and 5b together form a uniform band that is rich in fibers compared to L-4. The remaining inner layers show an increase in fiber abundance compared to Area 19 (Rose, 1935).<br>Vogt and Vogt (1919) classified this subregion as “eu-, medio-, denso- and mixtoradial. Trizonal, eu- and strongly tenuifasciate, multofibrous with some strong individual fibers, eutangential and eucingulate. Dives, extremostriate, separatus. Conjunctostriate, intimolatior, tenuilimitatus” (see von Economo and Koskinas, 1925, p. 622).
**200 f**			Reports concerning the *area (subregio) occipitalis* by scholars of the Vogt school and on the examination of brains from the Vogt collection exist from M. Vogt [99], Beck E[80], Heinze G [100,101] and Busch K-T [83]. All authors studied the cytoarchitecture of this subregion.
Subregio* (*A.) striata		VV[34]	*Area 17 (Brodmann), Area striata (granulosa] (OC, von Economo and Koskinas, 1925).*<br>The myeloarchitecture of two divisions of this subregion is described by Vogt and Vogt (1919). Fig. 22: “infra-, medio-, modico- und fino-radial. Quadrizonal, crasso- and multo- fibrous, ultratangential, interno-dyscingulate, pauper, asingulofibrous, separatus; tenui-intrastriate, intimoaequalis, tenuilimitatus”; Fig. 34: “eu-, medio-, denso- and mixtoradial. Trizonal, eu- and strongly tenuifascial, multo- and very crassofibrous. Eutangential, interno-dyscingulate”. See also von Economo and Koskinas, [63], p. 651-653).<br>Rose [113] characterized this subregion as typically trizonal which high numbers of basal fibers in L-1 a +b. L-3 is narrow, the radii are closer to the cortical surface than in any other euradiate type of cortex. L-3 is followed by a slightly more fiber-rich L-4a. Next follows an extremely fiber-rich Bechterew (Gennari) stripe. Below the outer Bechterew stripe follows a fiber-poor L-4c, which corresponds to L-IVc in the cell image. L-5a shows more fibers than L-4c and L-5b even more than L-5a. L-6a is characterized by numerous, very thick individual fibers. These individual fibers recede strongly in the sublayers of L-6 which stands out in dark color because of the increase of basic fibers.

(Continued)

**220 f**	Parcellations of Area (Subregio) striata by scholars of the Vogt school and studying brains from the Vogt collection are available from Beck [80] who described 11 subfields [cited in Ngowyang, 54, 61] and Ngowyang G (1934) who distinguished 16 cytoarchitectonic subfields (in the brain A 61l).

Abbreviations: 17=Area striata (= Str, [99]); 18=Area occipitalis (= O, [99]); 19=Area praeoccipitalis (= PrO, [99]); BA = Brodmann area; Bf=basic- or ground-fibers; calc=Fissura calcarina; d=densus; Ef / If=single/ individual fiber; EK=von Economo and Koskinas [63]; el=externolatior; f1s, f2s=S. fusiformis; Ff=fiber plexus; fus=S. fusiformis; Hf=horizontal fiber; if1, if2=S. collateralis; L=Lungwitz [40] (figure nr.]; l1 und l2=S. lingualis; Ng=Ngowyang [60]; O=occipital area; ot=S. occipito-temporalis; otl=S. occipoto-temporalis lateralis; otr=S. occipito-transversalis; otr=S. occipitalis transversus; p= prope / parietal; pl=S. lingualis; pm=S. paramesialis; po=Sulcus parieto-occipitalis; poc=S. paraoccipitalis; p =S. parieto-temporalis; R=Rose M [113] see Suppl. 2; Sf=oblique fiber; t=tenuis; Tf=tangential fiber; to=S. temporo-occipitalis; VV=[16] (figure nr.).

Classification of the pre-occipital fields by Lungwitz [40]

	103	104	105	106	107	108	109	110	111	112	113	114	115	116	117	118	119		
	edl	eda	edt	elt	eld	pt	elsc	edpc	dpp	del	pc	scet	sced	scd	sct	scel	pp		
trizonal	+	+	+	+	+	+	+	+	+	+	+	+	+	+	+	+	+		
eufasciate	+	+	+	+	+	+	+	+	+	+	+	+	−1	+	+	−1	+		
tenuifasciate (medio-)	+	2	−1	+	+	−1	2	3	+	+	+	−1	+	3	3	+	+		
multofasciate	3	3	2	+	−1	+	+	3	3	−1	−1	+	+	−1	−1	−1	2		
crassofibrös	2	+	−1	−1	−1	**−1**	+	2	+	2	−1	2	−1	−1	−1	−1	+		
eutangential	+	+	+	+	+	+	+	+		+	+	+	+	+	+	+	+		
eucingulate	+	+	+	+	+	−1	+	+		+	−1	−1	+	−1	+	−1	+		
externostriate									−1	−1									
trisingulofibrous	3	−1	−1	−1	−1	+	+	3-4		−1	+	−1	−1	−1	−1	+	−1		
propeprofundosuprastriate							−1											4	very intense
dives	+	+							+			−1							
pauper																+		3	intense
separatus	+	+	+	+	+	+	+	+	+	+	−1	+	+	3	+	−1	+		
grossofibrate	−1	−1	−**3**	−1	−1	2	−1	2	3	−1	2	2	−1	−1	2	−1	2	2	strong
externodensior	4	2	−1	+	+	3	+	+	3	−1	+	+	2	+	2	−1	+		
externolatior	3	+	2	+	+	+	+	+	2	+	+	+	2	+	2	+	+	**1**	moderate
subconjunctostriate	+	+	+	+	+	−1	−1	+	−1	−1	+	+	+	+	+	+	−1		
tenuiintrastriate	3	+	+	+	+	−1	+	−1	−1	−1	+	+	+	+	−1	+	+	+	**low**
Intimolatior	+	+	+	+	+	−1	−1	+	−1	+	−1	+	−1	+	−1	+	3		
tenuilimitatus	−1	+	+	−1	+	−1	+	+	+	+	−1	+	−1	+	+	+	+	**0**	**neutral**
aequostriate						−1													
supraradiate		o−1							−1								1	−1	weak
latoradiate	o−2								+								1		
euradiate			+	2	+		+	+	+	+	+	+	+	+	+	+	+	−2	medium weak
medio-, crassoradiate		+	+	2	+	+	+	+	+	+	−1	−1	−1	+	+	+			
densoradiate	3	3	2	−1	2	+	2	3	4	4	−1	2	−1	2	+	2	+	−3	**very weak**
mixtoradiate	+	3	2	+	2	+	+	2	4	4	−1	2	−1	2	+	2	+		

SUPPLEMENT 4: Isocortex Temporalis

For our analysis of the cortex of the temporal isocortex we included the reports from Beck [80], Rose [113], particularly from Hopf [26,46,47] because those are the most detailed and recent studies and because results were at least in part confirmed by photometric analyses.

The first column shows the numbers of the distinguished myeloarchitectural fields for the temporal isocortex (fields 120–182). We differentiate between myeloarchitectonic characterized "fields" (F) and cytoarchitectonic characterized "areas" (A). The second to fourth columns separate subregions (SR) from divisions (Divisio, Div) and individual myeloarchitectural fields. The next column indicates where photos can be found that illustrates this field (H54: Hopf [46]; H68: Hopf [26]. In the last column ("Hopf [46]") the distinguishing features of the individual fields are listed in brief. Hopf's classification system is presented in a table at the end of this supplement.

			Diagrams, Photos	Comments	Hopf, [46]
1 . Regio temporopolaris (tp)			H54. Regio temporopolaris occupies the pole of the temporal lobe and extends on the dorsal surface caudomedially to the limen insulae and the parainsular gyrus and laterally and basally to the T2.<br>Medial, ventral, lateral, and dorsal subregions are distinguished of which the medial shows the lowest fiber density, while the most fibers can be found in the lateral subregion.<br>The entire temporopolar isocortex is characterized by its thickness, its lack of fibers and its singulostriate character. The fiber feltwork is intense only in the lamina tangentialis and in the outer stripe of Baillarger. Coarse Ef do not occur. As a result, no stripe of Kaes-Bechterew is noticeable.		
**Area**	***Subregio temporopolaris medialis tp.m***		H68. The medial subregion is separated from the laterally located fields by a deep sulcus which is constant in all brains.<br>Of the subregions of the temporal pole, the medial one (tp.m) is characterized by its broad cortex and a fiber density that is lower than in the other subregions. The only exception is L-1, which is emphasized here. The fiber content then gradually increases laterally toward tp.l, which has the greatest fiber abundance of all temporopolar fields.		
**120**	tp.m.i	A. interna	H54: I, 1.	**tp.m.i** is the most medial field of **tp**. It has a considerable extent and, with the exception of the caudolateral part, occupies the entire medial temporopolar gyrus. It borders caudolaterally the *Formatio mesocorticalis temporalis* (Brockhaus) of the *Cortex claustralis* and thus ends before the point at which the temporal lobe fuses at the temporal stem.<br>**tp.m.i** is extremely fibrous. L-1 is fairly broad and quadrizonal.	si. p. rr
**121**	tp.m.e	A. externa	H54: I, 2.	**tp.m.e** is a small field located on the medial temporopolar gyrus and essentially forms the medial lip of the medial temporopolar sulcus.<br>L-1 is trizonal; L-4 and Rb are accentuated.<br>**clearly singulostriate** (no stripe of Baillager recognizable)	si.p.rr

(Continued)

**122**	tp.m.p	A. posterior	H54: I, 3.	**tp.m.p** is a relatively small field that adjoins **tp.m.e** caudally and, like this, extends laterally to the base of the medial temporopolar sulcus.<br>L-1 is broad and quadrizonal and contains somewhat coarser Ef than tp.m.i. The field differs from **tp.m.i** and **tp.m.e** notably by the higher content in L-1 and a denser arrangement of the Rb.<br>**tp.m.p** forms a transition zone between the isocortical **tp** and the mesocortical entorhinal area.<br>**singulostriate**	si.p.1d.rr
**123**	tp.m.pt	A. postica (intima)	H54: IV, 1.	**tp.m.pt** at the mediobasal edge of the **tp** borders the entorhinal region. It stands structurally between the isocortical **tp.m.if** and the *Formatio mesocorticalis temporalis* (Brockhaus). Supraradiary fibers - typical for allocortical areas - are missing.<br>L-5a is lighter, L-5b somewhat darker, but an inner Baillarger stripe is not recognizable and this field is therefore classified as **singulostriate**.	si. p. rr
**124**	tp.m.if	A. inferior	H54:III, 5.	Fields 124-126 are divisions of the Area mesocorticalis temporalis medialis.<br>**tp.m.if** occupies the mediobasal area of the temporal pole between **tp.v.if** and **tp.m.i**. The caudal portion is partially covered by the beginning T4. The **euradiary** characteristic is only shown on intensely stained preparations.	si. p. rr
**125**	tp/mti			**tp/mti**, a transition zone between the isocortex and the mesocortical part of the entorhinal area, is essentially still isocortical, but already shows some features of the mesocortical areas.	si.p.1d.rr
**126**	tp/mtm		H54: II, 2.	**tp/mtm** forms the caudal continuation of tp.m.i at the transition between the isocortical pole region to the *Area mesocorticalis temporalis medialis*. Compared to tp/mti, it has significantly fewer fibers. It differs from tp.m.i mainly by a darker L-1 and a wider but less distinct L-4.	si.p.1d.rr
	Subregio temporopolaris ventralis tp.v		H68.		
**127**	tp.v.if			The inferior field extends from the temporal pole to the limen insulae. Its oral section lies mainly on the basal surface and only to a lesser extent on the lateral surface. Caudally this field becomes to lie in t2 so that it is no longer shown on the surface map. **tp .v.if** has very few fibers in all layers. It differs from the medially following **tp.m.if** by a better developed L-4; on the other hand, the its L-1 is less well	si. p. rr

(Continued)

				developed. Compared to the neighboring field **tp .v.s**, it has generally fewer fibers.	
**128**	tp.v.s	A. superior	H54: III, 3.	**tp.v.s** lies on the lateral surface of the temporal lobe pole below **tp.1**. Caudally it becomes overlayed by T2. This field is, like the **tp** fields **singulostriate**.	si. p. rr
	Subregio temporopolaris lateralis tp.l		H68. **tp.l** occupies the dorsolateral aspect of the polar region. It stands out from the neighboring fiber poor **tp** fields by its richeness of fibers and can therefore be well distinguished.		
**129**	tp.l			The caudolateral division is particularly rich in fibers.	si.p/d.Efrr
	Subregio temporopolaris dorsalis tp.d		H68.		
**130**	tp.d.e	A. externa	H54: III, 1.	**tp.d.e** lies on the dorsal surface of the temporal pole, specifically on the lateral portion of the *Gyrus temporopolaris proprius*. Caudally it extends well beyond the point of attachment of the temporal lobe.<br>The structure of **tp.d.e** is similar to **tp.v.s**, but has fewer fibers, especially in L-4-7. It differs from the laterally adjacent fields **tp.l** and **tsep.l.a** in its lesser fiber content, and from the medially adjacent **tp.d.i** in its higher fiber content.	si. p. rr
**131**	tp.d.i	A. interna	H54: II, 1.	**tp.d.i** lies on the medial half of the G*yrus temporopolaris proprius*, while the lateral half of this gyrus is occupied by **tp.d.e** . The field is directly adjacent to the *Sulcus temporopolaris medialis*. Compared with the laterally adjoining **tp.d.**e the field shows less fibers in L-3 and 4 than but more fibers in L-1.	si. p. rr
**132**	tp.d.p	A. posterior	H54: IV, 2.	**tp.d.p** locates on the caudomedial portion of the *Gyrus temporopolaris proprius*. It joins caudally to **tp .d.i** and further caudally reaches the parainsular fields. **tp.d.p** is a field with very few fibers in all layers. It is significantly brighter than the laterally adjoining **tp .d.e**, but darker (with the exception of L-1) than the medial bordering **mtl**.	si. p. r
	Subregio mesocorticalis, mt (Brockhaus)		On the dorsal surface of the temporopolar region is also found a (temporal) mesocortical formation (Brockhaus), considered by O. Vogt as oral sector of the entorhinal region. This subregion consists of 3 areas defined by Brockhaus (1940).<br>All three field are classified as **supraradiate**.		
**133**	mtm	A. mesocorticalis temporalis medialis	Diagram H54: 3		
**134**	mti	A. mesocorticalis temporalis intermedia	Diagram H54: 3		
**135**	mtl	A. mesocorticalis temporalis lateralis	Diagram H54: 3		

(Continued)

2. Regio temporalis separans (tsep) - (Subregio temporalis superior, Beck, 1928)			H54. The *Regio temporalis separans* lies wedge-shaped between the temporopolar region and the *Regio transversa*. With its lateral subregion it continues along the dorsolateral edge and thus occupies the anterior orolateral aspect of the first Heschl's gyrus, while the medial extension forms a narrow corridor between the *Regio transversa* and the *Regio parainsularis* .<br>This region differs significantly from the temporopolar region and is clearly distinguishable by the inner Baillarger stripe (almost not discerable in the temporopolar fields) which becomes increasingly dense and contrasted.<br>Other characteristics common to all fields of the *Regio separans* are increased fiber density, the appearance of the Kaes-Bechterew stripe (in 3^1) and the inner Baillarger's stripe. As the inner stripe reaches the level of the outer one a clearly **bistriate externodensior** or **bistriate aequodense** cortex type is described.		
	Subregio lateralis, tsep.l				
**136**	tsep.l.a	A. anterior	H54: V, 1. Diagr. 3-4; H68.	**tsep .l.a** joins caudally **tp .l** and abuts caudally onto **tsep.l.md**, **ttr.l ol.i**, **ttr.l om.a** and **tsep.m.e**. **tsep.l.a** has some resemblance to **tp .l**, but shows significantly more fibers. Unlike **tpa**, it has a K-B stripe and an inner Baillarger stripe. L-1 is quadrizonal, L-1a very narrow, L-1s is wide. L-2 contains several Gf . L-3-1 is rich in medium caliber Ef, while L-3 2 and 3 3 have few Ef but more Gf. A K-B stripe is clearly present. 4 is dark. L-5a stands out clearly.<br>The field is **propunistriate** and strongly **externodensior** with some resemblance to the singulostriate type. The Rb are of medium width, mixtoradiate and quite dense.	pru e. d.K-B.rr
**137**	tsep.l.md	A. media	H54: IV, 3; H68	**tsep.l.md** lies between **tsep .l.a** and **tsep.l.p.** Structurally it takes an intermediate position between both fields. Compared to **tsep.l.a**, the Rb are stronger and L-4 appears somewhat darker, since it contains more Ef; L-5b is significantly more pronounced, the K-B stripe is not as prominent as in **tsep .l.a**. Compared to **tsep.l.p**, L-5b in particular is less well developed. Overall, **tsep .l.md** has fewer fibers than **ttr .lol i, ttr.lol.md** and **ttr.lol .e**, which especially applies to L-3 and 5b. Layers 4-6 are wider than in **tsep.l.a** and **ttr.lol.e. tsep.l.md** is **propunistriate** to unistriate and externodensior.	pru e. d.K-B.rr
**138**	tsep.l.p	A. posterior	H54: V, 2; H68.	**tsep.l.p** lies on the oral portion of the **ti** and also extends onto the dorsal surface. It shows more fibers than the caudally adjoining **tpartr.a.** Layers 4-6 are wider than in **ttr.lol.e** and **md** . L-1 is quadrizonal with medium fiber content. L-2 shows	pru e. d.(K-B).rr

(Continued)

				only few fibers. There are more Ef in L-3-1 than in 3-2 and 3-3, but hardly any Gf can be recognized. However, from L-3-2 to L-3-3, Gf become increasingly abundant. A K-B stripe is hardly seen. L-4 is rich in Gf and Ef. L-5a stands out well, L-5b is brighter than L-4. L-6a is almost as fibrous as L-5b. **tsep.l.p** is thus **propunistriate** and weakly **externodensior**. The deep layers contain abundant Ef.	
**139**	tsep.l.pf	A. profunda	H54: V, 5. Diagr. 6-7.	**tsep.l.pf** extends along the greaterpart of the ventral surface of T1. Usually it joins tmag.d caudally and can then be seen on the same frontal sections as **tsep.l.md** and **tsep.l.p.** Caudally this field extends to **tpartr.a and -pf**.	pru/u e.p. (K-B).r
	Subregio medialis, tsep.m				
**140**	tsep.m.e			**tsep.m.e** lies immediately medial to the sulcus ttr1, which it accompanies as a narrow band. This field differs considerably from the **ttr 1** fields due to the marked reduction of fibers in all layers, particularly in L-3 and 5b. On the other hand, it has considerably more fibers than its medially adjoining fields. The gradual decrease in the fiber content ranging from the extremely fiber-rich **ttr 1** fields to **tsep.m.e** and -**i, tpari.1** and -**im** to the extremely fiber-poor **tpari.m**, enables the parcellation these fields. L-1 is tenuously quadrizonal. L-2 stands out well. A K-B stripe is present, however, with low contrast. L-3-3 is considerably richer in fibers than L-3-2. L-4 and 5b are of about the same density. L-5a stands out quite well. **tsep.m.e** is **bistriate** and **aequodens**. The Rb are of medium density and mixtoradiate.	bi ä. d.K-B.rr.
**141**	tsep.m.i			**tsep.m.i** extends as a narrow band between **tsep.m.e** and **tpari.l.** It forms a transition area to the parainsular fields, while still belonging to the *regio separans*. Compared to **tsep.m.e**, **tsep.m.i** shows a reduced fiber density in all layers. A quadrizonal pattern of L-1 is hardly recognizable. L-2 is very low in fibers. A K-B stripe is difficult to identify. L-3-2 and 3-3 are quite bright and contain only very few thin Ef. Even within the deep layers, the Ef are not prominent. L-4 is somewhat darker than L-5b. L-5a and 6a are quite bright. The Rb are quite dense, are medium wide and slightly mixtoradiate. This field is of the **bistriate equidense** to slightly **externodense** type.	bi ä.p/d.(K-B) rr

(Continued)

3. Regio temporalis parainsularis (tpari)			H54; H68. The *Regio temporalis parainsularis* is the smallest of the temporal regions. It lies on the *Gyrus parainsularis*, a transition area close to the circular sulcus and the insular cortex, probably corresponding to the field TI between TeI and the insular cortex [84]. This region is characterized by its thin cortex, very poor fiber density. In spite of the low fiber content both Baillarger stripes can be recognized (distinct **bistriate** type).<br>The absence of Gf and even Ef is particularly striking in L-3. A K-B stripe is not seen. Compared to the temporopolar region, this region shows fewer myelinated Gf, especially in L-3.		
Subregio parainsularis					
**142**	tpari.l	A. lateralis	H54: II, 4.	As there is a general increase of fibers from medial to lateral in the parainsular group this lateral field is the most fibrous of them. It clearly stands out against the lateral areas of the *Regio separans* with its much lower fiber density.<br>The lamination is rather apparent. The sublayers of L-1 are not clearly distinguishable; L-3, albeit poor in fibers and without Ef in all sublayers, stands out against the bright L-2. No K-B stripe is distinguishable. L-4 and 5b are about the same density. L-5a stands out well. **tpari .l** is **bistriate** and **aequodense** . The deep layers contain very few Ef. The Rb which stand not very close are weakly mixtoradiate.	bi ä/e.pr.
**143**	tpari.im	A. intermedia		**tpari.im** lies structurally between the neighboring **tpari-**fields. It shows therefore fewer fiber than **tpari.l** and more fibers than **tpari.m**.<br>L-3 hardly stands out against L-2 . An outer and an inner Baillarger stripe can be clearly seen, although both have very few fibers.	bi ä/e.pr
**144**	tpari.m	A. medialis	H54: II, 5.	The most medial parainsular field **tpari.m** is next to the insula. The field has an extraordinarily low fiber content. The individual layers can only be distinguished by means of a microscope.<br>Ef are not present at all. L3-1 does not stand out against L-2 . The Baillarger stripes are extremely hard to recognize. The Rb are exeptionally light and thin.	bi ä/e.pr
4. Regio temporalis transversa (ttr)			H54; H68. The transverse temporal region (ttr) occupies the gyri Ttr1 and Ttr2. Of its 5 subregions, 4 are on the 1st and one on the 2nd transverse gyrus. Beck [80] also distinguished ttr3 as a region, which corresponds in extent to the planum temporale (Wernicke). Hopf [46] includes this area into the temporoparietal region.		

(Continued)

			The region differs significantly from all regions described above - with the exception of a small orolateral portion - as it has the highest fiber content of all temporal lobe regions. With the exception of the orolateral subregion, the inner Baillarger stripe is denser and more myelinated than the outer one. It is therefore described as **internodensior**. In some parts L-5b stands out better than L-6a, so that one can speak of a **bistriate** type. Compared to the *Regio separans (ts*), the following differences can be noted:<br>1. the small cortex thickness;<br>2. the higher degree of myelinated fibers in the lamina tangentialis;<br>3. the K-B stripe and the well discriminated L-3-3;<br>4. the inner Baillarger stripe is darker than the outer one (internidensior type), whereas in the *Regio separans* a externodensior or aequodense type can be seen and in the *Regio temporopolar*is a singulostriate type is typical;<br>5. layers 5-6 also have more fibers;<br>6. the number and thickness of the Ef is enormous;<br>7. the radii become thicker and more dense.		
	1 . Subregio transversa prima orolateralis , ttr .1ol				
**145**	ttr.1ol.i			The internal field lies on the medial slope of the oral part of the gyrus Ttr1 but does not reach the fundus of ttr1 (where **ttr.1om.a** is still located). It shows a well-developed K-M stripe.	pru i. d. rB.rrr.
**146**	ttr.1ol.md			The medial field lies on the crest of the oral part of the gyrus Ttr1. It has an extremely dark appearance due to the significantly higher fiber density compared with **ttr.1ol.i and -e** whereby the difference in L-1 and L-3-1 is greatest.	pru i. d. rB.rrr.
**147**	ttr.1ol.e			**ttr.1ol.e** lies on the lateral slope of the oral portion of Ttr1.Thsi field is the lightest of the orolateral fields of Ttr1 as the content of fibers is reduced in all layers compared to the other fields.	pru i.d. K-B
	2. Subregio transversa prima caudolateralis, ttr.1cl		This subregion is characterized by an intense K-B stripe, dark L-1 and an abundance of coarse Ef in all layers, while Gf are not as significant.		
**148**	ttr.1cl 2			**ttr.1cl** lies on the lateral slope of the middle section of the 1st transverse gyrus. In some brains, the field (rarely) also extends to the adjacent lip of Ttr2. (The field has less Gf but more Ef than **ttr .1cm.a**. The L-1 is somewhat darker than in **ttr .1cm.a**.	pru i. d. rB.rr.
	3. Subregio transversa prima oromedialis, ttr.1om		This subregion has fewer fibers than the two subregions that lie on the crest. The deep layers, which are somewhat narrower, can be better distinguished from one another. The anterior part of the oromedial subregion shows a particularly strong L-3.		
**149**	ttr.1om.a	A. anterior	H54: VI, 4.	**ttr.1om.a** lies on the medial slope of the gyrus Ttr1 and extends to the sulcus ttr1. Orally it starts	bi ä. d. 3Ef rr

(Continued)

				somewhat posterior to **ttr.1ol.i** and reaches well to mid-length of Ttr1. The field is narrower in layers L-4-7 than the lateral adjoining fields. The deep layers also have fewer fibers and can be better differentiated from one another. On the other hand, L-3 shows more Ef than the neighboring **ttr.1ol** fields.	
**150**	ttr.1om.p	A. posterior	H54: VI, 5.	**ttr.1om.p** represents the continuation of **ttr.1.om.a** on the medial slope of the 1st transverse gyrus. It is less fibrous than the laterally adjoining **ttr.1cm.a** and the orolaterally attached **ttr.1ol.i** and **ttr.1om.a.**	pru i/ ä.d.Ef. rr
	4. Subregio transversa prima caudomedialis, ttr.1cm				
**151**	ttr.1cm.a	A. anterior	H54: VII, 1.	**ttr.1cm.a** is usually on the caudal half of Ttr1, but it can also extend orally to the border of the 1st and 2nd third of Ttr1. The darkening of the Ttr1 fields reaches a maximum here.	pru i.d Ef. rrr
**152**	ttr.1cm.ep	A. externoposterior	H54: VII, 2.	**ttr.1cm.ep** lies on the caudal end of Ttr1, namely on its outer half.	pru i.d.Ef. rr
**153**	ttr.1cm.ip	A. internoposterior	H54: VII, 3. Diagr. 10.	**ttr.1cm.ip** lies on the medial half of the caudal end of Ttr1. Overall, it has significantly fewer fibers than the fields **ttr.1cm.a** and **ttr.1cm.ep** which are located laterally and orally.	pru i. d.Ef.rr
	5. Subregio transversa secunda, ttr.2		The fields that make up this subregion are much more different from each other than the fields of the subregions located on the Ttr1.		
**154**	ttr.2.ae	A. anteroexterna	H54: VII, 5.	**ttr.2.ae** forms the caudal continuation of **tsep.l.p** on the dorsal surface (orolateral portion of Ttr2). The structure is comparable to the *Regio separans* although darker than this field, it is significantly lighter, however, than the fields of Ttr1. It is **equidense** to slightly **externodensior**, in contrast to the other fields of the *Regio transversa*, which are all internodensior.	pru i. a/ e.d.rB.rr.
**155**	ttr.2.ai	A. anterointerna	H54: VIII, 1.	**ttr.2.ai** occupies the portion of **Ttr2** adjacent to **Ttr1** and is structurally quite similar to the fields of **Ttr1** (less fibers than **Ttr1**, but more than **ttr.2.ae**, which occupies the lateral portion of Ttr2). The field is significantly darker than **ttr.2.ae**, almost as rich in fibers as the **ttr.1** fields and, like these, **internodensior** . A K-B stripe is perceptible as well as numerous Ef in all layers.	pru i. d.rB.rrr.
**156**	ttr.2.pe	A. posteroexterna	H54: VIII, 2.		

(Continued)

				**ttr.2.pe** covers the middle part of the 2nd transverse gyrus and extends between **ttr.2.ai** and **tpartr.s**. The field has more fibers,is thus darker, especially in the L-4, than **ttr.2.ae**, but does not reach the density shown in **ttr.2.ai**.	pru i. d.rB.rrr.
**157**	ttr.2.pi	A. posterointerna	H54: VIII, 3.	**ttr.2.pi** forms the caudomedial end of the 2nd transverse gyrus. It is more fibrous than the preceding field **ttr.2.pe**, especially in L-3, also somewhat in L-4, but not in L-5b, so that the **internodensior** character slowly disappears.	pru i. d.rB.rrr.
5. Regio temporalis paratransversa, tpartr			H54, H61; H68.	**Tpartr** occupies the caudal two thirds of the lateral T1 and extends to the planum temporale. If a third transverse gyrus is present (Beck, 1928) it is covered by **tpartr**.	
**158**	tpartr.pf	A. profunda		**tpartr.pf** forms the caudal continuation of **tsep.l.pf**. It occupies the fundus of the ventral **T1**. Due to its lower fiber content, it clearly stands out from **tpartr.a**, which covers the lateral and part of the ventral surface of T1 .	pra.p.Ef. r
**159**	tpartr.a	A. anterior		**tpartr.a** is part of the middle third of T1. It covers the dorsal lip and lateral surface of L-1. The field is decidedly brighter than **tpartr.p** and **ttr.2.pe**.	pra.p.Ef. rr
**160**	tpartr.p	A. posterior		**tpartr.p** occupies the lateral surface of the posterior half of the t1 but also encroaches to the dorsal surface. The field is more fibrous than **tpartr.a**. L-3 and 4 are lighter than in **tpartr.s, ttr.2.pe** and **ttr.2.ai** L-5a is darker than in **ttr.2.ae**.	pra.p. Ef. rr
**161**	tpartr.s	A. superior		**tpartr.s** forms the medial portion of the cortex of Ttr3. It has a general lower fiber density than Ttr2. Especially the Ef are reduced. In contrast, the field is significantly richer in fibers, especially in L-3, than the laterally adjoining field **tpartr.p**.	pra.p.Ef. rr
6. Regio temporalis magna (tmag)			H54.	The *Regio temporalis magna (tmag)* occupies the 2nd and 3rd temporal gyri (the dorsal subregion occupies mainly T2, the ventral subregion mainly occupies T3). The border between both subregion runs only partially in the gyral fundus. The two caudal subregions differ from the dorsal and ventral subregions by the greater fiber content. The region is generally poor in fibers but shows some difference between the ventral part with low fiber density in L-3 and the caudal part which has significantly more fibers. The radial fiber bundles are narrow.<br>The *Regio magna* is clearly distinguished from the temporopolar region by the presence of the inner Baillarger stripe. It differs from the *Regio separans* and *Regio paratransversa* by a much lower fiber content, the absence of Ef in L-3 and the slender Rb.	

(Continued)

			Compared to the *Regio limitans*, the outer Baillarger stripe is much less prominent.		
	1. Subregio magna dorsalis, tmag.d		**tmag dorsalis** occupies T2 and extends caudally to T3. Their fields are uniform, orally still **externodensior**, in caudal direction progressing to the **internodensior** type. The fiber content is greatest in the oral and dorsal areas and lowest in the caudal and ventral areas. As a result, **tmag.d.as** has the most fibers and **tmag.d.p** has the least fibers.		
**162**	tmag.d.as	A. anterosuperior	H54:XI, 4.	The anterosuperior field lies at the transition between the temporal pole and T1/T2 joining caudally **tp.v.s**. It can neither be clearly allocated to the *Regio temporopolaris* nor the *Regio separans* or *magna*. Hopf tentatively classifies this field as part of the *Regio temporalis magna*.	ue.p/d.rr
**163**	tmag.d.aif	A. anteroinferior	H54 IX, 1.	The anteroinferior field occupies the oral portion of T2.	ue.p.rr
**164**	tmag.d.md	A. media	H54:XI, 1.	The *Area media* joins caudally to **tmag.d.aif** on T2; caudally it is followed by **tmag.d.p**	uä.p.rr
**165**	tmag.d.s	A. superior	H54:XI, 2.	The superior field lies on the middle third of T2, dorsal to **tmag.d.md**. In dorsal direction it borders on T1 (**tpartr.pf** and **-p**). The field is somewhat richer in fibers than **tmag.d.md**, but contains significantly fewer fibers than the T1 fields at the corresponding levels.	uä.p.rr
**166**	tmag.d.p	A. posterior	H54 XI, 3.	The posterior field occupies together with **tmag.cd.if**, **-s** and **-p** about the caudal third of T2 and usually extends into the T3. It is brighter than **tmag.cd.if**, **-s** and **-p**, but darker than the ventrally adjacent **tmag.v.as**.	ui.p.rr
	2. Subregio magna ventralis., tmag.v		This subregion occupies the oral two-thirds to three-fourths of the T3. The fiber content reaches a minimum with L-3-1 and 3-2 being almost devoid of fibers. Rb are extremely narrow and delicate. The two Baillarger stripes are roughly similarly low in fibers. The -**pif** field has, however, even fewer fibers.		
**167**	tmag.v.as	A. anterosuperior	H54 IX, 2.	The anterosuperior field is the most extensive of the areas located on the T3. It usually occupies the complete oral part of T3; in the middle sector only on the upper (dorsolateral) part and caudally at about the anterior third of T3. The field is lighter than **tmag.d.aif** and -**md** and **tlim.o.a**, but darker than **tmag.v.pif**.	pru ä.p.rr
**168**	tmag.v.pif	A. posteroinferior	H54 IX, 3.	The posteroinferior field is on the middle part of T3 below **tmag.v.as**. It clearly differs from the neighboring **tmag.v.as** in that the Rb are less pronounced.	uä/i .p.rr
	3. Subregio temporalis magna caudodorsalis , tmag .cd		This subregion is richer in fibers than the two previously mentioned. While the two oral fields are still **internodense**, in the two caudal areas the outer Baillarger stripes are prominent thus indicating the transition to the adjoining fields of the occipital lobe.		

(Continued)

**169**	tmag.cd.if	A. inferior	H54 X, 1 .	The inferior field covers the caudal third of T2 between **tmag.d.p** and **tmag.cd.s**. It is usually separated from **tmag.d.p** by a furrow. It is slightly more fibrous than **tmag.d.p** and **tmag.d.s and** only slightly less fibrous than **tmag.cd.s**.	pru i.p.rr.
**170**	tmag.cd.s	A. superior	H54 X, 2.	The superior field adjoins **tmag.d.s** caudally and adjoins **tpartr.p** dorsally. The field is generally somewhat more fibrous than **tmag .cd.if** and somewhat more pronounced, and therefore **internodensior**. The Rb are stronger and more numerous than in the ventrally adjoining fields;, the difference is small, however. **tmag.cd.s** is darker than **tmag.d.p** and **tmag.d.s.**	pru i.p.rr
**171**	tmag.cd.p	A. posterior	H54 X, 3.	This field forms the caudal end of T2, adjacent to T1. It aligns with **tmag.cd.s**, which is quite similar. **tmag.cd.p** is more fibrous and has wider Rb than **tmag.d.p** and **tmag.cd.if**. The field is thus **unitriate** to **propeastriate** and weakly **externodensior**. Ef are found in L-4-6 in moderate amounts. The Rb are medium wide, **mixtoradiate** and are of medium density.	u/pra e.p.rr
**172**	tmag.cd.lim	A. limitans	H54 X, 4.	The structure of tmag.cd.lim (*Area limitans*) translates to the occipital fields. Due to its greater fiber content and the special density of L-4, it can be easily distinguished from the neighboring temporal fields.	si/a e.p/d.rr
	4. Subregio caudoventralis, tmag .cv		The caudoventral subregion, like the last one described, differs from the dorsal and ventral subregions by a higher fiber content. Its oral field clearly shows features that appear evoked by the proximity of the T4 fields, while the caudal field appears influenced by the occipital fields.<br>The outer stripe of Baillarger is less prominent in both fields than in the occipital fields. It is also much less prominent in the overall picture than in the oral T4 fields because the deep layers are more distinct. On the other hand, it is more conspicuous than in the ventral and dorsal subregion because the outer Baillarger stripe is thicker.		
**173**	tmag.cv.a	A. anterior	H54 IX, 4.	The anterior field begins as a small region in initial part of t3 and only caudally reaches the surface. Its characteristic is quite similar to the orally neighboring **tlim.o.md**, but shows an increase in fibers in L-5 and 6. L-4, on the other hand, is less prominent than in **tlim.o.md** and **tlim.o.a**. L-5a does not stand out as much as in **tlim.o.md**, but the difference between L-5a and 5b is larger because L-	si/u e.p.rr

(Continued)

				5b and L-6 are significantly richer in fibers than in **tlim.o.md**. **tmag.cv.a** takes anintermediate position between **singulostriate** and **unistriate**.	
**174**	tmag.cv.p	A. posterior	H54 IX, 5.	The most caudal field on T3 is darker than **tmag.d.p**, but lighter, especially at L-4, than the adjacent fields of the occipital lobe.	u e.p/d.rr
7. Regio temporalis limitans (tlim)			H54.		
Subregio oralis, tlim.o					
**175**	tlim.o.a	A. anterior	H54:XII, 1.	The anterior field occupies the oral portion of T4. It is more fibrous than **tmag.v.as, tp.m.if** and **tp.m.pt,** which is particularly impressing if L-4 is compared.	pru/si. p.rr
**176**	tlim.o.md	A. media	Phot . Taf . XII, Diagr. 7-11.	This field is quite similar to **tlim.o.a**, to which it lies caudally and medially, differing only by its smaller amount of fibers. On the other hand it has more fibers than **tp.m.if** and **tmag.v.as.** Hopf distinguishes two subfields (extrema and intima) depending on the amount of fibers particularly in the deep layers. **tlim.o.md** is **singulostriate**,	u/si. p.rr
**177**	tlim.o.p	A. posterior	H54 XII, 3. Diagr . 12.	The very small posterior field is located caudally to **tlim.o.md** and structurally very similar except for layers L-5b-7, which are distinctly darker. They contain both more Gf and more Ef. L-4 is also somewhat darker, but the difference in this layer is not as clear. The field is **unistriate** and **externodensior** while **tlim.o.md** is singulostriate.	ue. p.rr
**178**	tlim.o.i	A. interna	H54 XII, 6. Diagr . 3-12.	The internal field is for the most part hidden in the rhinal fissure; is therefore not shown on the surface view of the published maps. Only a smaller caudal part reaches the surface. A caudal part of **tlim .o.i** may form part of the lining of the occipitotemporal sulcus. The cortex thickness is low, especially in layers L-4-7. Within this field is a region, still isocortical, that may be regarded as transition zone between tlim.o.i and the entorhinal region. Here L-4 is less well defined than in the rest of the tlim.o.i because the deeper layers are more fibrous and coarser. L-4-7 do not stand out and the Rb contain somewhat coarser fibers than in **tlim.o.i**. L-1 is not as dark as in the entorhinal area and a stratification, typical for the entorhinal region, is not found.	si. p. r
Subregio caudalis, tlim.c					
**179**	tlim.c.e	A. externa	H54:X, 6.	The external field of **tlim** forms the caudal end of the T4. Medial to it lies **tlim.c.i**, caudally the occipital	u/pra äd.rr

(Continued)

				areas. **tlim.c.e** is in L-4, 5b and 6 significantly darker than **tmag.cv.a** and **tlim.o.p**.	
**180**	tlim.c.i	A. interna	H54 X, 5 .	The internal field of **tlim.c.**lies on the caudal end of the T4. It borders laterally **tlim.c.e**, caudally and medially the occipital fields. **tlim.c.i** is quite similar to **tlim.c.**e, but the outer stripe of Baillarger is more prominent. **tlim.c.i** is slightly richer in fibers than **tlim.c.e .at**. Compared with the adjoining occipital fields **tlim.c.i** has fewer fibers. However, this difference is minute and it is therefore quite difficult to separate these fields properly.	ue.d.rr
	Subregio medialis, tlim.m				
**181**	tlim.m.e	A. externa	H54:XII, 5.	The small external field of **tlim.m** is sandwiched between the allocortex and occipital lobes. It forms the caudal continuation of entorhinal *subregio caudolimitans* (**eclimv,** Sgonina, 1937). The occipital fields adjoin this field caudally. The cortex thickness of **tlim.m.e** is small, especially layers 4-7. The field is macroscopically similar to **tlim.o.i** but L-4 and the Rb show higher contrast.	si.p.rr.
**182**	tlim.m.i	A. interna	H54 XII, 4. Diagr . 11-12.	The internal field of **tlim.m** is - like **tlim.m.e** - placed between the allocortex and the occipital fields. It is a small field, medial to **tlim.m.e**, and likewise typical for its narrow cortex width. While **tlim.m.e** is much more similar to the field **tp.m.if**, **tlim m.i** already shows a considerable likeness to the occipital cortex types due to the stronger development of L-4. **tlim.m.i**, like tlim.m.e, is **singulostriate** and **asingulofibrous**.	si.p.rr

Annotation: Beck [80] names the isocortical temporal lobe as follows: **SR temporopolaris (tp) [**P. medialis (tpm), P. lateralis (tpl)]

SR temporalis superior (ts) [P. medialis (tsm), P. lateralis (tsl)]

SR parainsularis (tpar)

SR temporalis transversa prima (ttrl) [P. intima (ttrlin), P. interna (ttrli), P. externa (ttrle), P. extrema (ttrlex), P. lateralis (ttrli)}.

SR temporalis transversa secunda (ttrlI) [P. medialis (ttrll m), P. lateralis (ttrlI 1)]

SR temporalis transversa tertia (ttrlll) [P. medialis (ttrIIIm), P. lateralis (ttrIIIl)]

SR temporo-parietalis (tpt).

Abbreviations: aequilatus=equilateral (with equal sides); B1=outer Baillarger stripe (Gennari stripe; IV. Lamina granularis); BA=Brodmann area; B2=inner Baillarger stripe (layer Vb); BA=Brodmann area; Ef= individual / single fibers (*Einzelfasern*); mostly horizontally oriented; F=field; Fb=*Fiberbündel*; Ff=ground fibers (*Faserfilz*); Gf=ground fibers; basic fiber network (*Grundfasern*); Gr=Griseum; Hf=horizontally oriented fibers (*Horizontalfasern*); L=layer, sublayer; Pre = Lamina principalis externa; Pri=Lamina principalis interna; Rb=radially oriented fiber bundle (Radiärfaserbündel); Rf=radially oriented fibers (*Radiärfasern; Radiäreinzelfasern*); Sf=oblique fibers; (*Schrägfasern*); Srf=supraradiary fibers (*Supraradiärfasern*); Tf=tangential fibers (*Tangentialfasern*).

Classification by Hopf [46]

**120** tp.m.i	**121** tp.m.e	**122** tp.m.p	**123** tp.m.pt	**124** tp.m.if	**125** tp./mti	**126** tp./mtm	**127** tp.v.if	**128** tp.v.s
si. p. rr	si.p.rr	si.p.1d.rr	si. p. rr	si. p. rr	si.p.1d.rr	si.p.1d.rr	si. p. rr	si. p. rr
**129** tp.l	**130** tp.d.e	**131** tp.d.i	**132** tp.d.p	**133 mtm**	**134 mti**	**135 mtl**	**136** tsep.l.a	**137** tsep.l.md
si.p/d.Efrr	si.p.rr	si. p. rr	si. p. r				pru e. d.KB.rr	pru e. d.KB.rr
**138** tsep.l.p	**139** tsep.l.pf	**140** tsep.m.e	**141** tsep.m.i.	**142** tpari. I	**143** tpari.im	**144** tpari. m	**145** ttr.1ol.i.	**146** ttr.1ol.md
pru e. d. (KB).rr	pru/u e.p. (KB).r	bi ä. d. KB.rr.	bi ä.p/d. (KB) rr	bi ä/e.pr.	bi ä/e.pr	bi ä/e.pr	pru i. d. rB.rrr.	pru i. d. rB.rrr.
**147** ttr.1ol.e	**148** ttr. 1cl.	**149** ttr.1om.a	**150** ttr.1om.p	**151** ttr.1cm.a	**152** ttr.1cm.ep	**153** ttr.1cm.ip	**154** ttr. 2.ae	**155** ttr. 2.ai.
pru i.d. KB	pru i. d. rB.rr.	bi ä. d. 3Ef rr	pru i/ä.d. Ef. rr	pru i.d Ef. rrr	pru i.d. Ef. rr	pru i. d. Ef.rr	pru i. a/e.d. rB.rr.	pru i. d. rB.rrr.
**156** ttr.2.pe	**157** ttr. 2.pi.	**158** tpartr.pf	**159** tpartr.a	**160** tpartr.p	**161** tpartr.s	**162** tmag.d.as	**163** tmag.d.aif	**164** tmag.d.md
pru i. d. rB.rrr.	pru i. d. rB.rrr.	pra.p. Ef. r	pra.p. Ef. rr	pra.p. Ef. rr	pra.p. Ef. rr	ue.p/d rr	ue.p.rr	uä.p.rr
**165** tmag.d.s	**166** tmag.d.p	**167** tmag.v.as	**168** tmag.v.pif	**169** tmag.cd.if	**170** tmag.cd.s	**171** tmag.cd.p.	**172** tmag.cd.lim.	**173** tmag.cv.a
uä.p.rr	ui.p.rr	pru ä.p.rr	uä/i .p.rr	pru i.p.rr.	pru i.p.rr	u/pra e.p.rr	si/a e.p/d.rr	si/u e.p.rr
**174** tmag.cv.p	**175** tlim.o.a	**176** tlim.o.md	**177** tlim.o.p	**178** tlim.o.i	**179** tlim.c.e	**180** tlim.c.i	**181** tlim.m.e	**182** tlim.mi
u e.p/d.rr	pru/si. p.rr	u/si. p.rr	ue. p.rr	si. p. r	u/pra äd.rr	ue.d.rr	si.p.rr.	si.p.rr

HOPF [46] **p. 252 ff:** Explanations and abbreviations.

1. Appearance of the stripes of Baillager: si = singulostrate; bi = bistrite; pru = propeunistriate; u = unistriate; pra = propeastriate; a = astriate; e = externodensior; ä = aequodensus; i = internodensior.
2. Overall content of fibers: (p = pauper, d = dives); 3p = extremely few fibers, 2p = very few fibers, 1p = few fibers. p/d = medium level of fibers (from pauper to dives), d = rich in fibers, d2 = high level of fibers, d3 = extreme richness of fibers.
3. Behavior of the fibers in individual layers and of the Ef (singular fibers): 1d = L-1 dives, (KB) = KB-stripe is weakly indicated, KB = KB- stripe is well recognized, KB = KB-stripe is obvious. Ef = Ef clearly stand out in the overall picture of the area.
4. Appearance of the radially aligned fiber bundles and fibers (Rb, Rf): r = Rb are moderately dense, rr = Rb are medium dense, rrr = Rb stand very tight. The thickness of the individual Rf increases at moderate density from r to rr to rrr; at medium density from 1r to 2r etc..
